# Genetic variants near *MLST8* and *DHX57* affect the epigenetic age of the cerebellum

**DOI:** 10.1038/ncomms10561

**Published:** 2016-02-02

**Authors:** Ake T. Lu, Eilis Hannon, Morgan E. Levine, Ke Hao, Eileen M. Crimmins, Katie Lunnon, Alexey Kozlenkov, Jonathan Mill, Stella Dracheva, Steve Horvath

**Affiliations:** 1 Department of Human Genetics, David Geffen School of Medicine, University of California Los Angeles, Los Angeles, California 90095, USA; 2 University of Exeter Medical School, University of Exeter, Exeter EX2 5DW, UK; 3 Department of Genetics and Genomic Sciences, The Friedman Brain Institute, Icahn School of Medicine at Mount Sinai, New York, New York 10029-6574, USA; 4 Davis School of Gerontology, University of Southern California, Ethel Percy Andrus Gerontology Center, 3715 McClintock Avenue, Los Angeles, California 90089-0191, USA; 5 Department of Psychiatry and Friedman Brain Institute, Icahn School of Medicine at Mount Sinai, New York, New York 10029-6574, USA; 6 James J. Peters VA Medical Center, Bronx, New York 10468, USA; 7 Institute of Psychiatry, King's College London, London SE5 8AF, UK; 8 Biostatistics, School of Public Health, University of California Los Angeles, Los Angeles, California 90095, USA

## Abstract

DNA methylation (DNAm) levels lend themselves for defining an epigenetic biomarker of aging known as the ‘epigenetic clock'. Our genome-wide association study (GWAS) of cerebellar epigenetic age acceleration identifies five significant (*P*<5.0 × 10^−8^) SNPs in two loci: 2p22.1 (inside gene *DHX57*) and 16p13.3 near gene *MLST8 (*a subunit of mTOR complex 1 and 2). We find that the SNP in 16p13.3 has a *cis*-acting effect on the expression levels of *MLST8* (*P*=6.9 × 10^−18^) in most brain regions. In cerebellar samples, the SNP in 2p22.1 has a *cis*-effect on *DHX57* (*P*=4.4 × 10^−5^). Gene sets found by our GWAS analysis of cerebellar age acceleration exhibit significant overlap with those of Alzheimer's disease (*P*=4.4 × 10^−15^), age-related macular degeneration (*P*=6.4 × 10^−6^), and Parkinson's disease (*P*=2.6 × 10^−4^). Overall, our results demonstrate the utility of a new paradigm for understanding aging and age-related diseases: it will be fruitful to use epigenetic tissue age as endophenotype in GWAS.

## Abstract (web summary)

This genome-wide association study identifies five significant SNPs in two loci which are associated with the epigenetic age of post-mortem cerebellar tissue according to a DNA methylation based biomarker of human aging.

## Graphical abstract

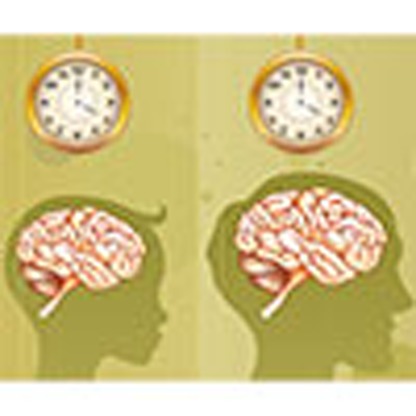

Substantial evidence suggests that lifespan is under genetic control but the decades long quest for human longevity genes has thus far only identified two genome-wide significant loci*: APOE* and a locus on chromosome 5q33.3 (refs 1, 2, 3). A third locus (*FOXO3A*) is probably associated with exceptional longevity^4^,^5^,^6^. These sobering results demonstrate that very large sample sizes will be needed to find the genetic determinants of human longevity. For example, Deelen *et al*^3^ used about 100 thousand subjects to detect the locus on 5q33.3. An alternative strategy for finding longevity genes is to replace the complex trait (age at death) by a molecularly defined ‘endophenotype', that is, a more stable phenotype with a stronger genetic link.

DNA methylation levels are a natural candidate for defining a molecular endophenotype of aging because epigenetic mechanisms probably play a role in modulating lifespan^7^,^8^,^9^ and because chronological age has a profound effect on DNA methylation (DNAm) levels^10^,^11^,^12^,^13^,^14^. We recently developed an epigenetic measure of tissue age by combining the DNAm levels of 353-dinucleotide markers known as Cytosine phosphate Guanines or CpGs^15^. The weighted average of these 353 epigenetic markers gives rise to an estimate of tissue age (in units of years), which is referred to as ‘DNA methylation age' or as ‘epigenetic age'. This epigenetic clock method for estimating age seems to apply to any tissue or cell type that contains DNA (with the exception of sperm) including sorted cell types (helper T cells, neurons and glial cells), complex tissues, and organs (blood, brain, bone, breast, kidney, liver and lung^15^,^16^,^17^) and even prenatal brain samples^18^. The epigenetic clock gives rise to promising molecular endophenotypes of aging because it captures aspects of biological age according to the following recent findings: the epigenetic age of blood has been found to be predictive of all-cause mortality even after adjusting for a variety of known risk factors^19^,^20^. Further, the blood of the offspring of Italian semi-supercentenarians (that is, subjects aged 105 years or older) has a lower epigenetic age than that of age-matched controls^21^. The epigenetic age of blood relates to cognitive and physical fitness in the elderly^22^ and to Parkinson's disease status^23^. The epigenetic age of the frontal lobe relates to neuropathological variables and to Alzheimer's disease related cognitive functioning^24^. The utility of the epigenetic clock method has been demonstrated in applications surrounding obesity^16^, Down syndrome^25^ and HIV infection^26^.

Here we pursue the strategy of using a measure of epigenetic age acceleration (DNAm age adjusted for chronological age) as endophenotype for biological age/mortality in a genome-wide association study (GWAS). We focus on the cerebellum for two reasons: (a) we are interested in studying aging effects in a relatively homogeneous nervous tissue (which is mostly comprised of cerebellar granule cells), and (b) epigenetic age acceleration is highly heritable in this brain region as described below. We identify five single-nucleotide polymorphisms (SNPs) in 2p22.1 and 16p13.3 that are associated with cerebellar age acceleration (*P*<5.0 × 10^−8^). These SNPs are significantly associated with expression levels of *DHX57* (in 2p22.1) and *MLST8* (16p13.3), respectively. Further, genes associated with cerebellar age acceleration also significantly overlap with those identified in other GWAS of various age-related diseases. Our results show that genetic studies of epigenetic age acceleration may not only illuminate the mechanism underlying the epigenetic clock but also identify genes that relate to various age-related diseases.

## Results

### Data sets

We studied post-mortem cerebellar samples from *n*=555 subjects of European ancestry by combining five different studies (studies 1–5 in Table 1). The chronological age at time of death ranged from 1 to 105 years. The study involved slightly more males (63%) than females (37%). Both cerebellar DNAm data and corresponding SNP marker data were available for each subject. Some of the studies included additional brain regions (Table 1) and corresponding transcriptional data (Supplementary Table 1). For example, study 6 involved DNAm, SNP and neuronal gene expression data, which were used in our *cis*-expression quantitative trait locus (QTL) studies. The genomic platforms (for example, Illumina DNAm array) and available SNP data are described in Supplementary Tables 1 and 2, respectively. While our primary GWAS aimed to identify SNPs that are associated with the epigenetic age of the cerebellum, we also related the resulting SNPs to epigenetic age acceleration in other brain regions and to transcriptional data, as described below.

### DNA methylation age and epigenetic age acceleration

The epigenetic clock is defined as a multivariate prediction method of age, based on the linear combination of the DNAm levels of 353 CpGs dinucleotides^15^. The resulting age estimate, referred to as DNAm age, is in units of years. By construction, the epigenetic clock (and software) applies to data generated on the Illumina Infinium DNAm array platform. An online age calculator can be found at our webpage: http://labs.genetics.ucla.edu/horvath/dnamage/.

As expected, the DNAm age of cerebellum is highly correlated with chronological age (*r* ranging from 0.64 to 0.96, Supplementary Fig. 1). The lowest correlation *r*=0.64 was observed in study 1, which involved older subjects (mean age 86 years) and a narrow age range (from 55 to 105 years). The highest correlation (*r*=0.96) was observed in study 5 that involved subjects with the broadest age range (from 1 to 102). In our QTL analysis, we used an age-adjusted measure of DNAm age (referred to as epigenetic age acceleration), which was defined as the residual resulting from a linear model that regresses DNAm age on chronological age. By definition, this measure of epigenetic age acceleration is not correlated (*r*=0) with chronological age. A negative value for age acceleration indicates that the sample is younger than expected based on chronological age.

### Heritability of age acceleration in different brain regions

We used the GCTA software tool^27^,^28^ to estimate the heritability of epigenetic age acceleration in different brain regions collected from the same neurologically normal subjects. The heritability of age acceleration of the cerebellum (*h*^*2*^=69% in study 2, *h*^*2*^=15% in study 5) appears to be higher than that of the frontal cortex (*h*^*2*^<0.1% in study 2, *h*^*2*^=14% in study 5), pons (*h*^*2*^<0.1% in study 2) or temporal cortex (*h*^*2*^=59% in study 2). These results demonstrate that the cerebellum is a particularly promising brain region when it comes to GWAS studies of epigenetic age acceleration.

### GWAS analysis

**Figure 1:**
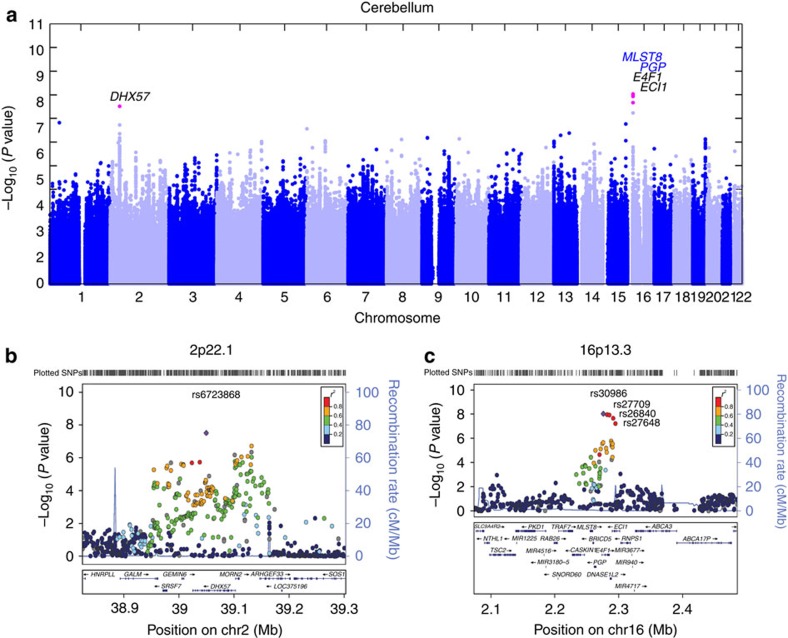
Genome-wide meta-analysis for epigenetic age acceleration in the cerebellum. (**a**) Manhattan plot for the meta-analysis results of 5 studies using cerebellar samples. A total of five SNPs associated with cerebellar age acceleration (*P*<5.0 × 10^−8^) are colour-coded in magenta, with their loci (gene names) listed on top. In additionally, two novel genes identified exclusively by *cis*-eQTL are colour-coded in blue. (**b**,**c**) present regional association plots for GWAS loci in 2p22.1 and 16p13.3, respectively. The association *P* values resulted from the meta-analysis that combined GWAS studies 1–5. (**b**) Region surrounding SNP rs6723868 (coloured in purple) in 2p22.1. The colours visualize linkage disequilibrium (LD) ***r***^2^ between rs6723868 and neighboring SNPs. (c) Region surrounding rs30986 (in purple color) in 16p13.3. The colors visualize the LD ***r***^2^ with respect to the SNP. SNPs rs27709, rs26840, and rs27648 also reach genome-wide significance levels.

To identify SNPs that are associated with cerebellar epigenetic age acceleration, we carried out a GWAS analysis in each of the five data sets. We analysed 5,713,604 genotyped or imputed SNP markers, based on the 1000 genome project reference panel (Methods). The level of genomic inflation was negligible (0.98≤λ_GC_≤1.01) in each individual GWAS. To combine GWAS results from the different data sets, we used fixed-effects meta-analysis, weighted by inverse variance^29^. We removed SNPs that exhibited substantial heterogeneity across the five studies (Cochran's *Q* test *P* value 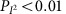). Only a moderate genomic inflation was observed in the meta-analysis *P*-values (*λ*_GC_=1.09; Supplementary Fig. 2). At a genome-wide significant level of *P*<5.0 × 10^−8^, cerebellar age acceleration was significantly associated with five SNPs (Table 2), which were located in two loci (Fig. 1a): locus 2p21.1 inside gene *DHX57* (Fig. 1b, Supplementary Fig. 3) and locus 16p13.3 near genes *MLST8* and *PGP* (Fig. 1c, Supplementary Fig. 4). In the following, we describe these two loci in more detail.

### Locus 2p22.1 near *DHX57*

The SNP rs6723868 is the only SNP in locus 2p22.1 that reaches genome-wide significance (*P*=3.1 × 10^−8^, Table 2) but it is surrounded by 25 SNPs (in high linkage disequilibrium 0.61<*r*^2^<0.83) with suggestive significance levels (*P*<1.0 × 10^−5^). The amount of heterogeneity in the meta-analysis is concerning (*I*^2^=65%, 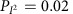; see Table 2 and Supplementary Fig. 3); however, it reflects the insignificant results for study 5 (as can be seen from the fact the heterogeneity *I*^2^ drops to zero after excluding study 5 from the meta-analysis). Since study 4 involved a small sample size (*n*=36 cerebellar samples), we validated its results by carrying out two types of robustness analyses: (a) we removed potential outliers and (b) we carried out a robust correlation analysis (Supplementary Fig. 5).

While rs6723868 is not associated with a protein-coding mutation of *DHX57*, this SNP is located either in chromatin state ‘weakly transcribed' or in state ‘transcriptional elongation' according to the 127 cell/tissue lines from the Roadmap Epigenomics/ENCODE project^30^,^31^,^32^, including 10 brain-related lines (see Methods and Supplementary Fig. 6a). Further, the SNP is associated with the gene expression levels of *DHX57* as described later.

### Locus 16p13.3 near *MLST8* and *PGP*

The most significant GWAS result for cerebellar age acceleration could be observed for rs30986 (*P*=9.3 × 10^−9^), which exhibits zero heterogeneity across studies (*I*^2^=0%, see Table 2 and Supplementary Fig. 4). While three neighbouring SNPs also reached genome-wide significance (Table 2), we believe that they capture the same underlying locus for two reasons. First, the four SNPs are in high linkage disequilibrium (pairwise LD 0.70<*r*^2^<0.98). Second, after conditioning on rs30986, the three remaining SNPs are no longer significantly associated with cerebellar age acceleration (Supplementary Table 3).

For over 99% of cell lines from the Roadmap Epigenomics Consortium, SNP rs30986 is located in a region that is either actively transcribed or plays a role in enhancing gene regulation (Supplementary Fig. 6b). Within 20 kb of rs30986 are six genes: *MLST8, PGP*, *E4F1*, *ECI1*, *DNASE1L2*, and *BRICD5* (Fig. 1c). The gene expression levels of *MLST8* and to a lesser those of *PGP* are associated with the SNP as will be shown in the following.

### *Cis*-expression QTL studies of GWAS hits

To gain a mechanistic understanding of our significant SNPs, we correlated them with messenger RNA levels of neighbouring genes. We used two broad categories of data sets, for which both SNPs and brain gene expression data were available: The first category involved gene expression data from the same subjects that were also used in our GWAS study (expression data from *n*=1224 brain tissue samples listed in Table 1). The second category involved archived eQTL results from the Brain eQTL Almanac (BRAINEAC, see URL)^33^ that used *n*=1231 brain tissue samples, across 10 brain regions, from 134 neurologically normal subjects of European ancestry.

The statistical analysis steps for finding gene transcripts that correlate with our GWAS hits is detailed in the Methods section entitled ‘*Cis*-expression QTL analysis of GWAS hits'. This approach led to the identification of two significant candidate genes (*MLST8* and *PGP)* for locus 16p13.3 and one candidate gene (DHX57) for locus 2p22.1 as described in the following.

### 2p22.1 has a *cis*-effect on the expression levels of *DHX57*

The minor-allele count of SNP rs6723868 (in locus 2p22.1) is negatively correlated with the expression levels of its neighbouring gene *DHX57* in the cerebellum (meta-analysis *P*=1.3 × 10^−5^ across studies 2, 3 and 5, see Supplementary Table 4) and frontal cortex (*P*=5.1 × 10^−3^ in study 5). We also found suggestive *cis*-effect (cerebellum, *P*=0.09, Supplementary Fig. 7a) using BRAINEAC. By combining the cerebellar results from our study with those from BRAINEAC, we obtained a meta-analysis *P*=4.4 × 10^−5^ (Stouffer's *Z* score method).

Interestingly, *DHX57* expression levels are positively correlated with chronological age in the cerebellum (cerebellar meta-analysis *P*=1.3 × 10^−19^, Fig. 3a, Supplementary Fig. 8a), possibly the frontal cortex (study 5, Fig. 3a) and neurons (study 6, Fig. 3a). We did not observe a significant correlation between the expression levels of *DHX57* and epigenetic age acceleration (that is, epigenetic age adjusted for chronological age) in our data (cerebellar meta-analysis *P*=0.66, all brain regions *P*=0.17), which might reflect technical reasons (and low sample size) or biological reasons (higher variability of messenger RNA levels).

### 16p13.3 has a *cis*-effect on *MLST8* and possibly *PGP*

**Figure 2:**
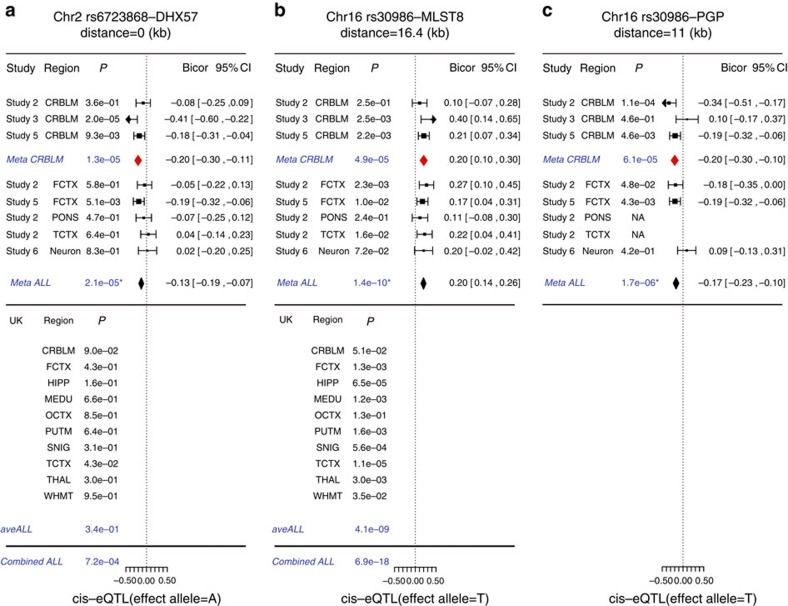
Brain *cis*-eQTL across 12 brain regions for age acceleration associated SNPs. The forest plots display the significant *cis*-eQTL results for pairs of (**a**) rs6723868—*DHX57*, (**b**) rs30986—*MLST8* and (**c**) rs30986—*PGP*, respectively. The top panels report *cis*-eQTL findings for 1224 brain tissues from the subjects used in our GWAS study of cerebellar age. We use the following abbreviations: cerebellum (CRBLM), frontal cortex (FCTX), pons (PONS), temporal cortex (TCTX) and assorted neurons. Each study reports robust correlation coefficients (bicor) with respect to minor-allele counts. Expression levels in CRBLM were available for studies 2, 3 and 5. Meta-analysis was used to combine individual results into a single estimate, *Meta CRBLM*. We combined results from additional brain regions (including the three CRBLM studies) into a single estimate, *Meta ALL*. The lower panel reports the results from the 1231 brain tissues archived in the BRAINEAC database. HIPP, Hippocampus; MEDU, medulla, OCTX, occipital cortex; PUTM, putamen; SNIG, substantia nigra; THAL, thalamus ; WHMT, intralobular white matter; *aveALL*, the average across all available regions. The expression data for the gene *PGP* were not available in BRAINEAC. The *Combined ALL P* value was calculated by combining the *Meta ALL* and *aveALL P* values using Stouffer's *Z* score approach. The footnote reports the *P* values that adjust for intrasubject correlation. Lower panels (BRAINEAC analysis) involved a) transcript ID 2549021, probe ID 2549027 and b) transcript ID 3466593, probe ID=3644621. We verified that the directionality is congruent (same effect alleles) between the upper and lower panels by inspecting the plots resulting from the option ‘stratification expression by SNP' option in BRAINEAC. As described in Methods, the *P* values were not adjusted for intrasubject correlation. After applying a decorrelation analysis, the Meta ALL *P* values become (**a**) 6.1 × 10^−4^, (**b**) 2.4 × 10^−7^ and (**c**) 7.5 × 10^−4^. Using these adjusted *P* values, the Combined ALL *P* values become (**a**) 5.0 × 10^−3^ and (**b**) 1.1 × 10^−14^.

**Figure 3:**
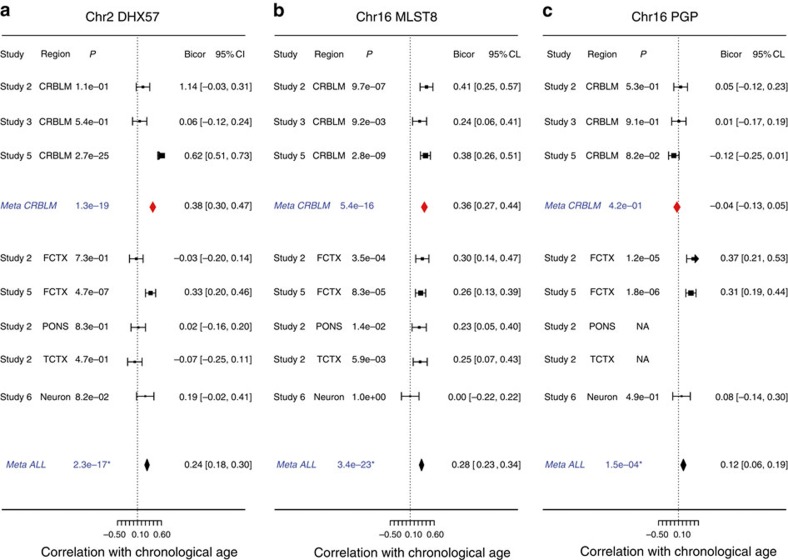
Correlation of potential functional genes with chronological age. The meta-analysis forest plots summarize the correlation between chronological age and the expression levels of (**a**) *DHX57*, (**b**) *MLST8* and (**c**) *PGP*, respectively. Each panel reports robust correlation coefficients based on our samples (up to 1224 brain tissues), as described in Fig. 2. The results from individual cerebellar data sets were combined into a overall estimate, *Meta CRBLM*. Similarly, the result from multiple different brain regions (including three from the CRBLM) were combined into an overall estimate, *Meta ALL*. As described in Methods, the *P* values were not adjusted for intrasubject correlation. After applying a decorrelation analysis, the Meta ALL *P* values become (**a**) 1.6 × 10^−8^, (**b**) 6.9 × 10^−13^ and (**c**) 1.5 × 10^−3^, respectively.

Our *cis*-expression QTL study shows that rs30986 has a highly significant positive correlation with the expression levels of *MLST8* in at least 9 brain regions (meta-analysis *P*=6.9 × 10^−18^, Fig. 2b, Supplementary Fig. 7b) including the cerebellum (meta-analysis *P*=4.9 × 10^−5^, see Fig. 2b and Supplementary Table 4), temporal cortex, hippocampus and substantia nigra. Interestingly, the expression levels of *MLST8* are significantly correlated with age acceleration in the cerebellum (meta-analysis *r*=0.11, *P*=0.030) but not in other brain regions. Further, the expression levels of *MLST8* increase with chronological age across multiple brain regions (robust correlation *r*=0.28 and *P*=3.4 × 10^−23^) especially in the cerebellum (*r*=0.38, *P*=5.4 × 10^−16^, Fig. 3b, Supplementary Fig. 8b). The expression levels of *PGP*, which is adjacent to *MLST8,* are also associated with SNP rs30986: its expression levels have a negative correlation with the minor-allele counts of SNP rs30986 in the cerebellum (meta-analysis *P*=6.1 × 10^−5^) and in the frontal cortex (Fig. 2c). Unfortunately, this gene is not available in the BRAINEAC database. However, unlike *MLST8*, cerebellar expression levels of *PGP* are neither correlated with chronological age (Fig. 3, Supplementary Fig. 8c) nor with cerebellar age acceleration (meta-analysis *P*=0.99). While our above mentioned results were obtained for the most significant SNP, rs30986, we briefly mention that the neighbouring genome-wide significant SNPs (rs27709, rs26840 and rs27648) lead to similar results for *MLST8* and *PGP*.

### Gene-set enrichment analysis for cerebellar age acceleration

In an effort to learn more about the biological processes that cause epigenetic age acceleration in the cerebellum, we applied the MAGENTA software^34^ (Methods) to test whether our meta-analysis GWAS results are enriched with sets of functionally related genes. While five gene sets (including DNA helicase) were nominally significant (4.8 × 10^−3^ ≤*P*≤2.1 × 10^−2^, Supplementary Table 5) these gene sets were not significant after multiple comparison correction (false discovery rate >0.10).

### Overlap with gene sets from other GWAS studies

To rank genes (as opposed to individual SNPs) based on our GWAS results we used the MAGENTA software to assign an overall *P* value per gene based on multiple underlying SNPs. Towards this end, MAGENTA assigns a *P* value to each gene by adjusting the most significant SNP-association *P* value (within the gene boundary ±50 kb) for gene size, number of SNPs in LD per gene, and other potential confounders^34^.

Further, we applied MAGENTA to rank the results from large-scale GWA studies (Supplementary Methods) of age-related macular degeneration (AMD)^35^, Alzheimer's disease^36^, longevity status (living longer than 90 years)^3^, and Parkinson's disease^37^.

We used each of the resulting gene rankings to define a corresponding set of significant autosomal genes by thresholding the MAGENTA *P* values at the 95th percentile. We used a one-sided hypergeometric test to assess the overlap between gene sets related to (1) cerebellar epigenetic aging and (2) those from age-related diseases, respectively. Strikingly, we found that the gene set that relates to cerebellar age acceleration significantly overlaps with that from AMD (hypergeometric test *P*=6.4 × 10^−6^, Table 3, Supplementary Table 6), Alzheimer's disease (*P*=4.4 × 10^−15^), and Parkinson's disease (*P*=2.6 × 10^−4^) but not with longevity status.

### Health and Retirement Study

We also used the same MAGENTA analysis to test whether our cerebellar aging gene set (defined above) overlaps with gene sets related to cognitive functioning in the Health and Retirement Study (HRS), a large-scale, nationally representative, longitudinal study of older adults in the US (*n*=12,452, see Supplementary Table 7). GWAS was performed on dementia status, as well as a longitudinal measure of age-related cognitive decline. We used dementia status from the two consecutive waves when SNP data were collected, conducting the association analysis for each wave separately, yielding a total of three cognitive functioning traits for assessment. For a given cognitive trait, we either restricted the GWAS analysis to a specific ethnic group or used all individuals in multivariate regression models that adjusted for principal components, estimated by identity by state (Supplementary Table 8, Supplementary Figs 9–11). Gene sets for the HRS were defined in the analogous manner for each cognitive functioning measure and study population. Overall, we only observed a marginally significant overlap between our cerebellar aging gene set and those related to cognitive decline and dementia in the HRS. The most significant results can be observed for participants of European or of African ancestry (5.9 × 10^−3^ ≤*P*≤0.048, Table 3, Supplementary Table 6).

## Discussion

To the best of our knowledge, this is the first article that (a) presents genome-wide significant SNPs associated with epigenetic age acceleration, (b) elucidates the underlying mechanism using *cis*-eQTL studies, (c) shows that the expression levels of one of the implicated genes (*MLST8)* increase with chronological age, (d) shows that epigenetic age relates to a subunit (*MLST8*) of both mTOR complexes and (e) shows a significant overlap between genes related to the epigenetic age of nervous tissue (cerebellum) and those implicated in AMD, Alzheimer's disease and Parkinson's disease.

This study has the following limitations. First, the cerebellum has, at best, a weak, indirect relationship with neurodegenerative diseases (such as Alzheimer's disease) or neurocognitive functioning traits. Nevertheless, the fact that we detected a significant overlap between age-related genes in the cerebellum and those of AMD, Alzheimer's disease and Parkinson's disease suggest that the cerebellum lends itself as surrogate tissue for tissues and cell types that are affected by the respective diseases.

The second limitation is that the GWAS study of epigenetic age only involved *n*=555 cerebellar samples. The identification of five significant SNPs is striking in light of the fact that a comparable sample size did not allow us to identify significant loci in blood tissue (unreported findings). The third limitation is that we did not assess whether changes in the expression levels of *MLST8* or *DHX57* cause changes in epigenetic age acceleration or vice versa. We were not able to carry out mechanistic studies in rodents because the epigenetic clock only applies to humans and chimpanzees. Given the rich literature on the role of mTOR in aging and age-related diseases^38^,^39^,^40^,^41^,^42^, it is striking that the expression levels of *MLST8* (a subunit of mammalian target of rapamycin complexes 1 and 2) relate to the SNP in the 16p13.3 locus in at least 9 brain regions (Fig. 2b).

Further, the finding that *DHX57* has a significant *ci*s-eQTL in the cerebellum is noteworthy given the following results from a comparative analysis of different brain regions. In our study, *DHX57* is significantly overexpressed in the cerebellum compared with other brain regions (*P*=1.9 × 10^−94^ in study 2 and 2.2 × 10^−49^ in study 5, Supplementary Fig. 12). Similarly, we previously found that genes involved in helicase activity are significantly overexpressed in the cerebellum compared with other brain regions (Bonferroni corrected *P*=8.5 × 10^−6^)^17^. Since our genetic study suggests that high RNA helicase activity is associated with a low cerebellar age, these results suggest that the cerebellum might age more slowly than other brain regions according to the epigenetic clock. This is indeed the case as we have recently shown using three independent data sets and brain regions from six individual centenarians^17^.

Overall, our results demonstrate the utility of a new paradigm for understanding aging and age-related diseases: instead of relating SNPs to clinical outcomes directly, it will be fruitful to use epigenetic tissue age as endophenotype.

### URLs

1000 genome project, http://www.1000genomes.org/

BRAINEAC, http://www.braineac.org/

DNAm age, http://labs.genetics.ucla.edu/horvath/htdocs/dnamage/

EIGENSTRAT, http://genepath.med.harvard.edu/~reich/Software.htm

HRS, http://hrsonline.isr.umich.edu/

IMPUTE2, https://mathgen.stats.ox.ac.uk/impute/impute_v2.html/

METAL, http://csg.sph.umich.edu/abecasis/Metal/

Locuszoom, http://csg.sph.umich.edu/locuszoom/

MaCH/Minimac, http://www.sph.umich.edu/csg/abecasis/MACH/

MAGENTA, https://www.broadinstitute.org/mpg/magenta/

PLINK, http://pngu.mgh.harvard.edu/~purcell/plink/

R metafor, http://cran.r-project.org/web/packages/metafor/

R WGCNA, http://labs.genetics.ucla.edu/horvath/CoexpressionNetwork/

SHAPEIT, https://mathgen.stats.ox.ac.uk/genetics_software/shapeit/shapeit.html.

## Methods

An overview of our data sets is presented in Table 1. Additional details can be found in Supplementary Tables 1 and 2 and in Supplementary Methods. The patient consent information can be found in the previously published articles. Further, our meta-analysis was approved by the ethics review board at UCLA (IRB#15-001479 and IRB#14-000061). Although our cerebellar DNAm data came from case–control studies of various diseases (Alzheimer's disease, schizophrenia and major depression), we ignored disease status in our analysis, since it was not significantly associated with cerebellar age acceleration. Studies 1–5 involved DNAm and SNP data measured from the same subjects. Furthermore, gene expression data (microarray or RNA-seq) were available for studies 2, 3, 5 and 6.

### Heritability analysis

We estimated the heritability of epigenetic age acceleration using data in different brain regions from neurologically normal subjects of studies 2 and 5 using the GCTA software tool^27^,^28^. In study 2, we focused on genotyped and imputed SNPs that met the following criteria: minor-allele frequency (MAF)>0.05, SNP missing rate<0.15, individual missing rate <0.10, Hardy–Weinberg equilibrium (HWE) test *P*>0.0001, and info measure >0.4 for imputed markers (Supplementary Table 2). The same criteria were applied to SNPs from study 5 but the individual missing rate, SNP missing rate and HWE were disregarded since the analysis was based on the expected allelic dosage. The limited sample size resulted in low power for detecting a significant level of heritability, for example, the power was less than 0.07 for detecting a heritability of *h*^2^=0.5 according to the GCTA-GREML power analysis tool^28^.

### GWAS analysis for epigenetic age acceleration

SNP quality was assessed by estimating MAF, HWE and missingness rate across individuals (Supplementary Table 2). European ancestry of the subjects from study 2 was validated by the authors^43^, which led to the removal of two inconsistent subjects. The reported genetic ancestry of other study subjects was confirmed using principal component analysis plots or multidimensional scaling plots in conjunction with principal component analysis in PLINK^44^ and EIGENSTRAT^45^.

### Imputation

We used IMPUTE2 (refs 46, 47) with haplotypes phased using SHAPEIT^48^ or MACH/Minimac^46^ to impute SNP and INDEL markers based on the 1000 Genome haplotypes from 1,092 individuals (released in December 2013). The quality of imputed markers was assessed by the Info measure >0.4 (in IMPUTE2) or *R*^2^>0.3 (in Minimac).

### Genome-wide meta-analysis

 For association analysis, we regressed the age acceleration trait values on (1) estimated genotype dosage (counts of test alleles) or (2) expected genotype dosage, adjusted for the first two principal components when necessary. Correlation or partial correlation estimates (if adjusted for the principal components) were used as the outcome measures in meta-analysis. More details stratified for each study can be found in Supplementary Table 2. We only analysed common variant markers (MAF>5%) for GWAS, leaving 5,713,604 (genotyped or imputed) markers present in at least 4 study sets for association analysis. We combined single outcome measures from each study by fixed-effects models weighted by inverse variance, as implemented in Metal^29^.

### Linkage disequilibrium analysis

All LD estimates presented in this article were calculated using the 1000 genome individuals with ancestry of European released in December 2013.

### Regional association results

We generated plots for presenting regional association results with LocusZoom^49^. As noted, color coded LD estimates presented in the plots were calculated using the 1000 genome individuals with ancestry of European released in November 2014.

### GWAS-based enrichment analysis with MAGENTA

We used the MAGENTA software^34^ to assess whether our meta-analysis GWAS results are enriched with Ingenuity pathways, KEGG pathways, Gene Ontology (GO) terms and PANTHER (biological processes, molecular functions and pathways) . To assign genes to SNPs, we extended the gene boundary to ±50 kb. For computational reasons, we removed categories that did not contain any genes related to age acceleration at a level of 1.0 × 10^−3^ or that contained fewer than 10 genes. For the GSEA method we chose 95th percentile, with empirical *P* values estimated started with 10,000 permutations then increased to 1 million when *P*<1.0 × 10^−4^. We only report the gene sets whose false discovery rate FDR (calculated under the MAGENTA algorithm) was <0.25.

### Chromatin state annotations for GWAS hits

For each genome-wide SNP, we used the UCSC genome browser to display the 25 chromatin states across 127 cell/tissue lines at 25-bp resolution (Supplementary Fig. 6s) based on imputed histone markers (from ChromImpute^32^). The *n*=127 diverse cell/tissue lines were profiled by the NIH RoadMap Epigenomics^30^ (*n*=111) and ENCODE projects^31^ (*n*=16). Additional annotation analysis results based on the earlier chromatin state analysis from ref. 50 are displayed in Supplementary Fig. 6s as well.

### *Cis*-expression QTL analysis of GWAS hits

In total, 2,455 brain tissue expression samples were available to identify genes whose expression levels were associated with our GWAS hits (Table 2). The expression data came from two broad categories of data. The first category involved our study subjects of 1,224 brain tissues across 5 brain regions and neurons, as listed in Table 1. The number already excludes the potential outliers detected by applying unsupervised hierarchical clustering analysis to the gene expression data from each brain region separately. We only removed a few suspicious samples as detailed in Supplementary Figs 13–16. The second category involved the BRAINEAC database with archived eQTL results evaluated in up to 1,231 brain tissues across 10 regions from 134 neuropathologically normal individuals of European descent (see URL). We evaluated the correlation between SNPs and gene expression levels using a robust correlation estimate known as biweight midcorrelation, which is implemented in the ‘bicor' R function of the WGCNA R package^51^. Our *cis*-eQTL involved all genes located within 500 kb of the test SNP and proceeded along the following three steps. First, we identified (*cis*-acting) SNP–gene pairs by using cerebellar gene expression data from subjects that were used in our cerebellar GWAS analysis. Towards this end, we used *n*=494 samples from studies 2, 3 and 5 for which cerebellar gene expression data were available. To combine the coefficient estimates from the three respective studies into a single estimate, we applied a fixed-effect model weighted by inverse variance (implemented in the ‘metafor' R package) and referred as to *Meta CRBLM* listed in Fig. 2. Genes surpassing *Meta CRBLM P* at 1.0 × 10^−4^ were highlighted for subsequent assessment. Second, we replicated these significant eQTL (identified in the first step) across other brain regions, using up to 730 brain tissues from our study samples. Expression QTL analysis was conducted on the expression data in frontal cortex for the same subjects in studies 2 and 4 plus pons and temporal cortex (for study 2 only), as well as in assorted neurons from 81 independent individuals (study 6 in Table 1). We combined a total of 8 eQTL results (including those from the first step) into a single estimate by the fixed-effect model, referred to as *Meta ALL* in Fig. 2. Third, additional eQTL results came from 1,231 brain tissues archived in the UK brain expression database. The eQTL was evaluated for up to 10 brain regions, including cerebellum, frontal cortex, hippocampus, medulla, occipital cortex, putamen, substantia nigra, temporal cortex, thalamus and intralobular white matter, in addition to the average across all available regions that yielded a single estimate for eQTL, listed as *aveALL* in Fig. 2. The effect allele under the UK database can be visualized in the plots listed under the option ‘stratification expression by SNP' (see Supplementary Fig. 7a,b). To summarize the eQTL results from the two categories of data, we applied Stouffer's *Z* score meta-analysis approach. Specifically, we combined the two *P* values from *Meta ALL* and *aveALL* into a single *P* value referred to as *Combined ALL* in Fig. 2. The resulting *Combined ALL P* value should be considered as descriptive (as opposed to an inferential measure) since it ignores the dependence resulting from the fact that various brain regions came from the same subjects in studies 2 and 4. To account for the intrasubject correlation, we applied a decorrelation analysis to the multiple brain regions in studies 2 and 4, yielding adjusted *Meta ALL* and *Combine ALL P* values (Fig. 2). However, the decorrelation analysis might be overly conservative since it may overcorrect *P* values. Details on the decorrelation analysis can be found in Supplementary Information.

### Overlap with the GWAS results

To yield the GWAS results for cognitive functioning traits, we used 12,500 participants from the HRS—an independent large-scale longitudinal data set with individuals over the age of 50 years collected every 2 years (Supplementary Table 7 and Supplementary Information). GWAS analysis was performed on genotyped and imputed SNPs for testing (1) cognitive slope that indicates the change in cognitive age given the change in chronological age over the fourteen years (1996–2010), (2) dementia binary status at wave 8 (diagnosed in year 2006) and (3) dementia binary status at wave 9 (diagnosed in year 2008), respectively. We used standard quality control for SNPs and assessments for association analysis results (Supplementary Information). For each trait, association analysis was conducted on all participants as well as in individual racial/ethnic strata (European, African American and Hispanic) resulting in a total of 12 GWAS analyses. Supplementary Table 7 lists the summary statistics of cognitive traits for all participants and different racial/ethnic strata. Supplementary Table 8 summarizes the model framework for association analysis and assessments for the GWAS results. Manhattan plots of the association results for each trait and race/ethnic group can be found in Supplementary Figs 9–11.

The genes were aligned according to the hg19 assembly (*n*=19,432 autosomal genes) except those of the longevity study (hg18 assembly, *n*=17677 autosomal genes).

## Additional information

**How to cite this article**: Lu, A. T. *et al.* Genetic variants near *MLST8* and *DHX57* affect the epigenetic age of the cerebellum. *Nat. Commun.* 7:10561 doi: 10.1038/ncomms10561 (2016).

## Supplementary Material

Supplementary Information Supplementary Figures 1-16, Supplementary Tables 1-7, Supplementary Notes 1-3 and Supplementary References

## Figures and Tables

**Table 1:** Overview of study data sets.

**Data**	**Age, years**	**% Male**	**Brain region**	***N***_**GWAS**_	***N***_***cis*****-eQTL**_	**Reference**	**Public availability**
Study 1	86±8.0 (55, 105)	38	CRBLM	59	NA	Lunnon *et al*,^52^	GSE59685
Study 2	48.0±23.2 (16, 96)	70	CRBLM	112	144	Gibbs *et al*,^43^	GSE15745GSE36192
			FCTX	—	144		
			PONS	—	143		
			TCTX	—	145		
Study 3	44.3±9.6 (19, 68)	63	CRBLM	147	130	Zhang *et al*,^53^	GSE35978GSE38873
Study 4	64.4±17.4 (25, 96)	61	CRBLM	36	NA	Pidsley *et al*,^54^	GSE61431
Study 5	52.3±29.8 (1, 102)	66	CRBLM	201	219	Hernandez *et al*,^55^	GSE36192GSE31694
			FCTX	—	218		
Study 6	30.7±10.8 (15, 65)	81	Neuron	—	81	Di Narzo *et al*,^56^	NA

CRBLM, cerebellum; FCTX, frontal cortex; NA, not available; N_c*is*-eQTL_, number of participants passing QC available for *cis*-eQTL analysis; N_GWAS_, number of participants passing QC available for GWAS analysis; PONS, pons; TCTX, temporal cortex.

— denotes the brain region data were not used for GWAS.

The first five studies involved a total of *n*=555 individuals that were used in our GWAS of cerebellar epigenetic age acceleration. Study 6 was used for a *cis*-expression QTL analysis in sorted neurons.

**Table 2:** SNPs that are significantly (*P*<5.0 × 10^−8^) associated with cerebellar epigenetic age acceleration.

**Band**	**SNP**	**Gene**	**Position (bp)**	**Minor/major alleles**	**MAF**	**EUR MAF**	**Corr. (s.e.)**	**Meta** ***P***	***I***^2^ **(%) (*****P*****)**
2p22.1	rs6723868	*DHX57*	39049601	A/G	0.26	0.27	0.23 (0.04)	3.1 × 10^−8^	65 (0.02)
16p13.3	rs30986	near *MLST8*	2275867	T/C	0.38	0.43	0.25 (0.04)	9.3 × 10^−9^	0 (0.5)
	rs27709	near *MLST8*	2281829	A/G	0.39	0.44	0.25 (0.04)	1.1 × 10^−8^	0 (0.6)
	rs26840	near *MLST8*	2285357	T/C	0.38	0.42	0.25 (0.04)	1.2 × 10^−8^	0 (0.5)
	rs27648	near *MLST8*	2291350	A/G	0.39	0.43	0.24 (0.04)	2.2 × 10^−8^	25 (0.3)

Corr., Correlation with respect to minor allele; EUR MAF, minor-allele frequency calculated using 1000 genome individuals with ancestry of European (released in December 2013); MAF, mean of minor-allele frequency estimates across studies weighted by study sample sizes; SNP, single, nucleotide polymorphism.

Position bp based on Hg19 assembly .

Fixed effects meta-analysis was used to estimate the correlation coefficient and standard error (‘Corr. (s.e.)') between the minor allele and epigenetic age acceleration across five studies. The corresponding meta-analysis *P* values can be found in the column ‘Meta *P*'.

**Table 3:** Overlap with gene sets found in other GWAS studies.

**Trait of GWAS study**	**Genetic ancestry**	No. of genes overlap/annotation*	**Hypergeometric** ***P***	**Bonferroni** ***P***
AMD	EUR+ASN	79/957	6.4 × 10^−6^	1.0 × 10^−4^
Alzheimer's disease†	EUR	29/100	4.4 × 10^−15^	7.1 × 10^−14^
Longevity (age >90)‡	EUR	25/880	>0.99	>0.99
Parkinson's disease	EUR	72/952	2.6 × 10^−4^	3.9 × 10^−3^
*Health Retirement Study*				
Cognitive decline	ALL	55/969	0.17	>0.99
	EUR	61/970	3.5 × 10^−2^	0.56
	AFR	57/970	0.11	>0.99
	AMR	50/969	0.42	>0.99
Dementia Wave 8	ALL	52/970	0.31	>0.99
	EUR	60/970	4.8 × 10^−2^	0.76
	AFR	49/969	0.47	>0.99
	AMR	54/968	0.21	>0.99
Dementia Wave 9	ALL	38/970	0.95	>0.99
	EUR	36/970	0.98	>0.99
	AFR	66/969	5.9 × 10^−3^	9.5 × 10^−2^
	AMR	48/968	0.53	>0.99

ALL, all genetic ancestry; AMD, age-related macular disease; AMR, Americans; AFR, Africans; ASN, Asians; EUR, Europeans; GWAS, genome-wide association studies..

^*^The proportion of trait related genes (MAGENTA *P*<95th percentile) that also relate to cerebellar age acceleration. The set of cerebellar aging genes contains *n*=967 autosomal genes that have a suggestive relationship with cerebellar age acceleration (MAGENTA *P*<95th percentile across autosomal genes).

^†^The small denominator numbers reflects the GWAS results of Alzheimer's diseases on 11,632 SNPs (Supplementary Information for more details).

^‡^94 out of 967 genes not in the human March 2006 (hg18) assembly had to be removed from the overlap analysis.
