# Supplementary material for: Genetic variants near MLST8 and DHX57 affect the epigenetic age of the cerebellum
Source: Nat Commun. 2016 Feb 2;7:10561. doi: 10.1038/ncomms10561 (PMC4740877; doi:10.1038/ncomms10561)
Supplement: Supplementary Information — Supplementary Figures 1-16, Supplementary Tables 1-7, Supplementary Notes 1-3 and Supplementary References [file ncomms10561-s1.pdf]

Supplementary Figures

Supplementary Figure 1: DNAm age versus chronological age

The figure depicts the scatter plots of DNA methylation age versus chronological age stratified by study. Each panel reports the Pearson correlation coefficients and a corresponding p-value.

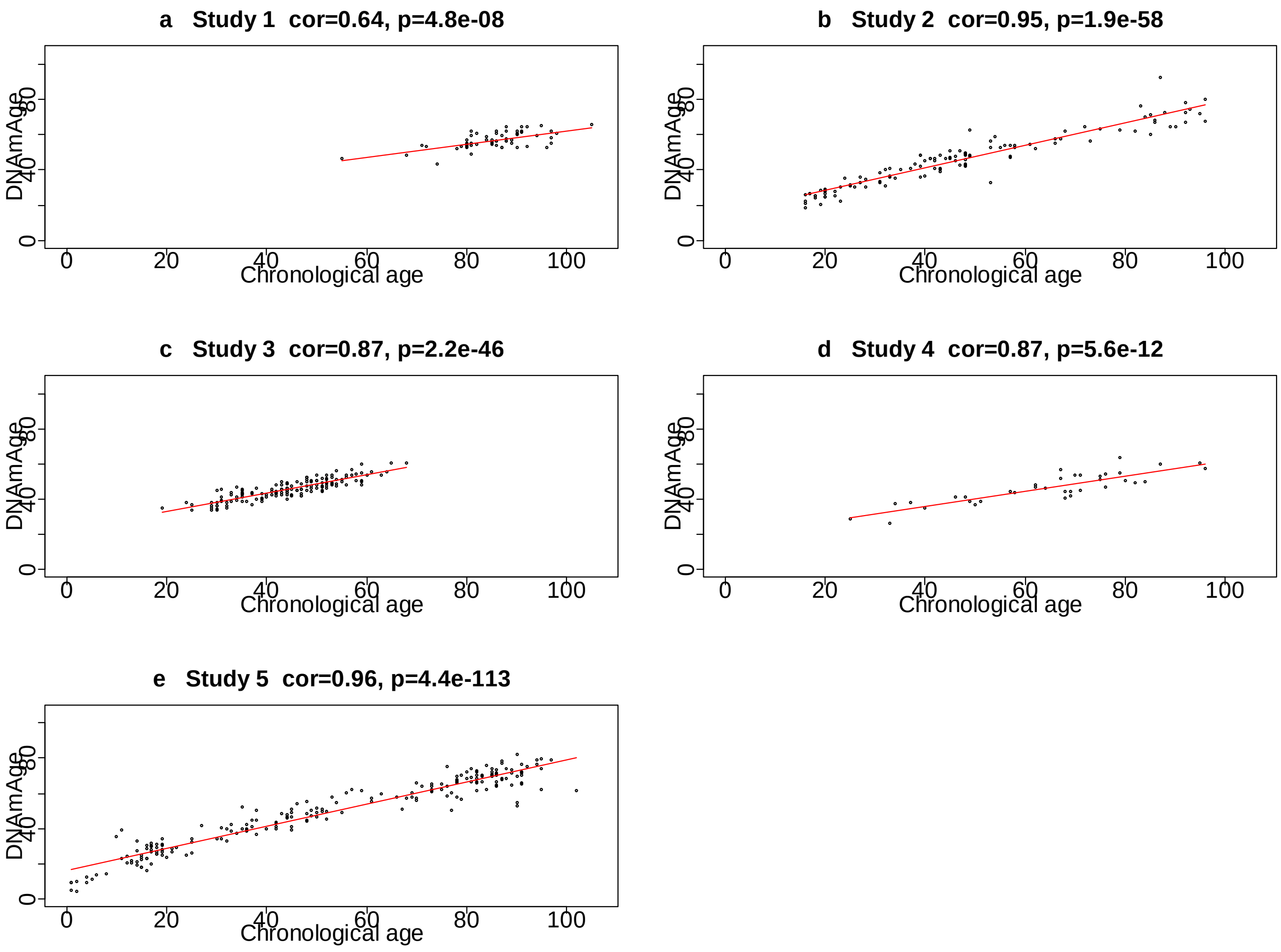

Supplementary Figure 2: QQ plots for evaluating genomic inflation in the meta-analysis

The black and red lines represent the observed and expected meta P values, respectively.

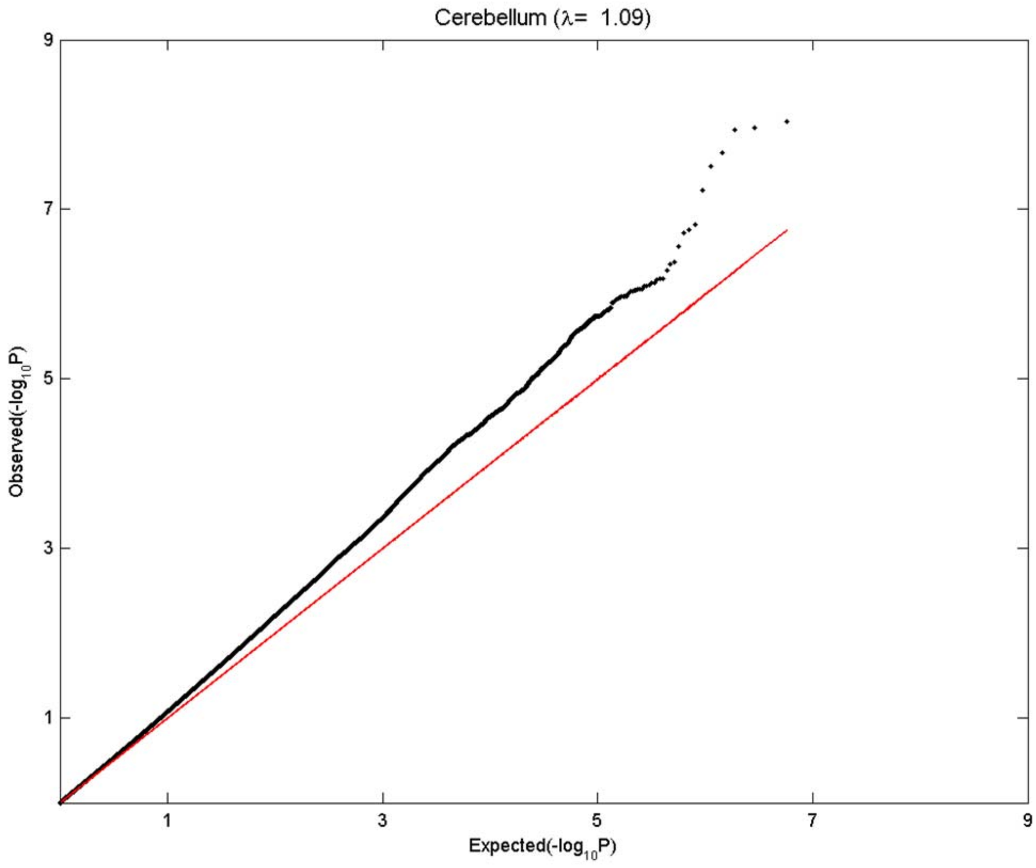

Supplementary Figure 3: Forest plot for relating rs6723868 with cerebellar age acceleration

In this fixed effects meta-analysis, the effects measure the correlation coefficient between the minor allele of the SNP and epigenetic age acceleration in the cerebellum.

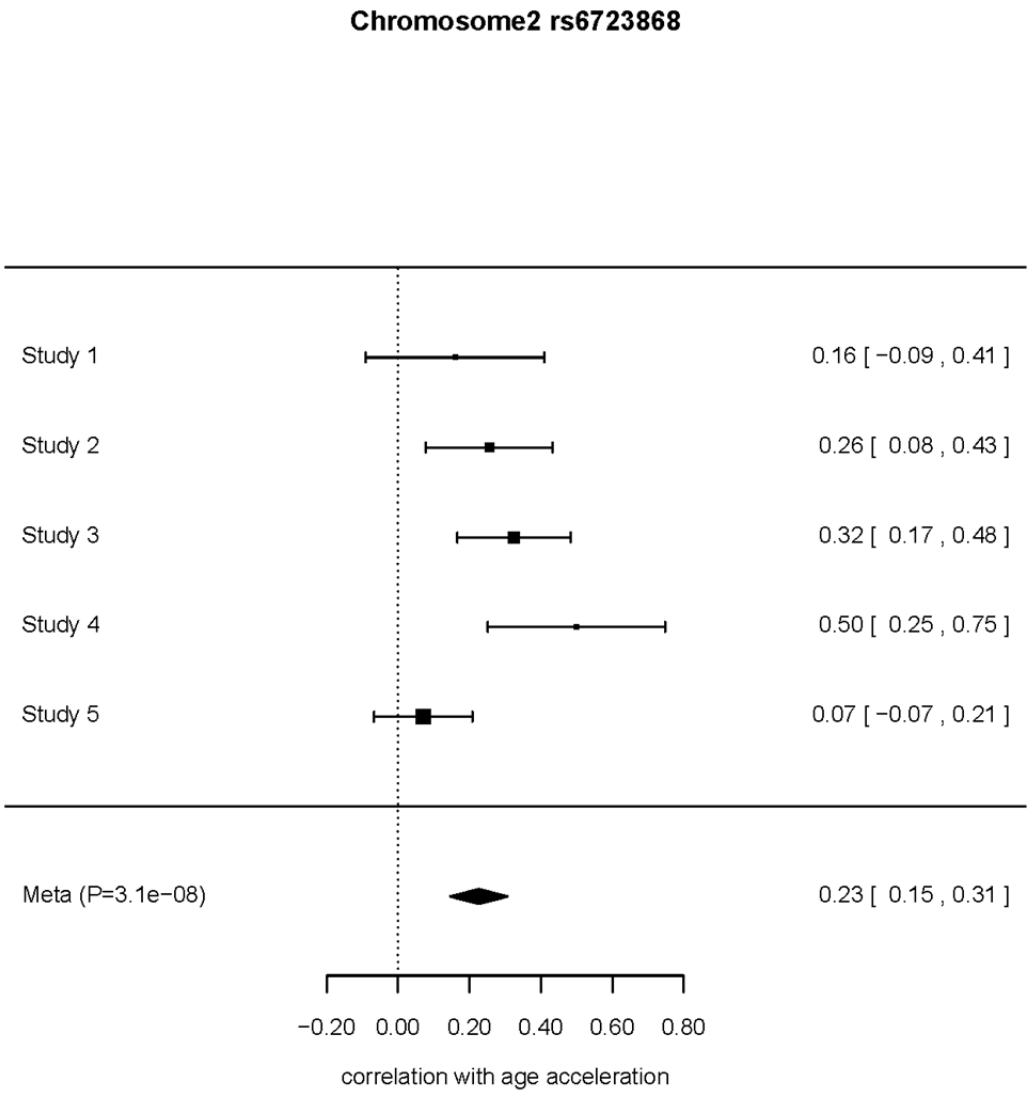

Supplementary Figure 4: Forest plot for relating rs30986 with cerebellar age acceleration

In this fixed effects meta-analysis, the effects measure the correlation coefficient between the minor allele of the SNP and epigenetic age acceleration in the cerebellum.

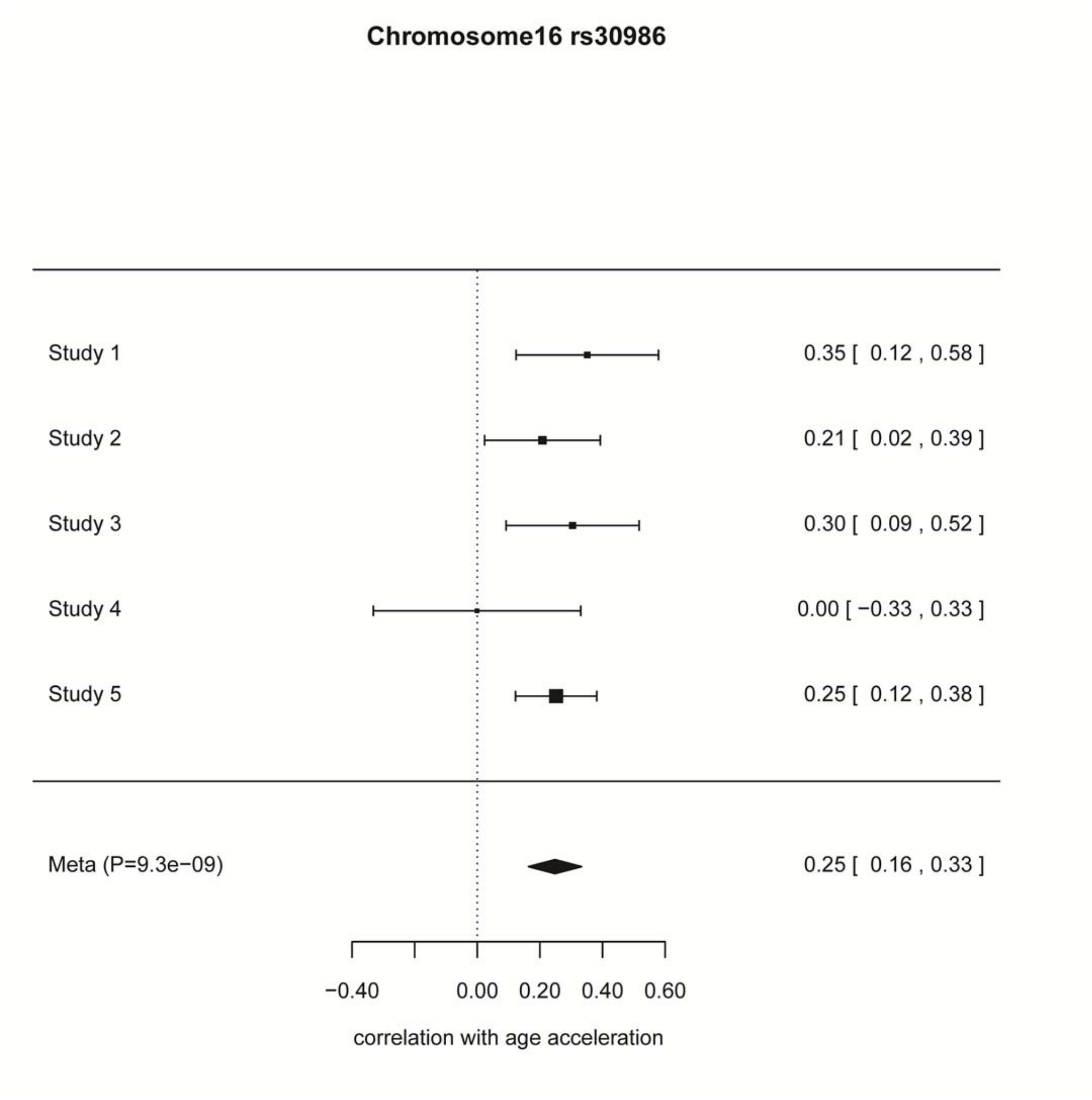

Supplementary Figure 5: Sensitivity analysis for study 4

The figure displays the scatter plot of age acceleration measure versus allele dosage of rs6723868 (on chromosome 2) in study 4 (N=36). Sensitivity analysis was conducted by calculating a robust correlation coefficient (biweight midcorrelation) [16]. The robust correlation coefficient 0.51 (P =1.5x10^{-3}) is very close to the Pearson correlation coefficient. In additional analyses, we removed (1) the three individuals with dosage close to zero and re-analyzed the association, resulting in a Pearson correlation estimate of 0.49 (P=4.5x10^{-4}), and (2) the two individuals with dosages close to 1 and age acceleration measures greater than 8, still yielding a significant Pearson correlation estimate of 0.5 (P=4.2x10^{-4}). These results show that the significant association signal for rs6723868 in study 4 is robust with respect to potential outliers.

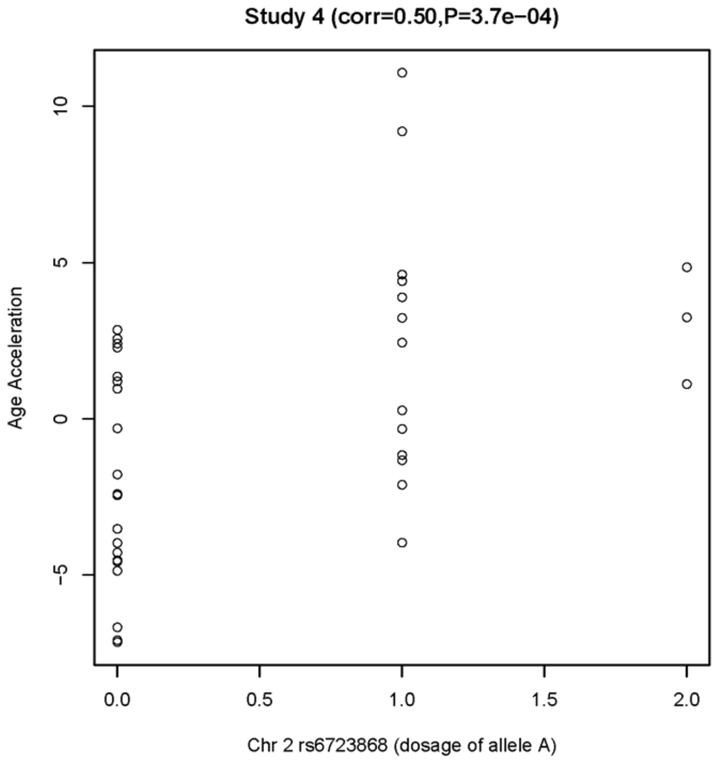

Supplementary Figure 6: Roadmap Epigenomics and ENCODE chromatin state analysis

Description: The plot summarizes the chromatin states for a study SNP across (1) 127 diverse human cell/tissue types from ChromImpute analysis[17] in the upper panel, and (2) 9 human cell types from ChromHMM[18] analysis in the lower panel. In (1) the hidden Markov model (HMM) classifies DNA regions dynamically into 25 states while in (2) the HMM classifies the regions into 13 states as described below.

The 25 states in ChromImpute

- State 1 - Red - TssA (Active TSS)
- State 2 - Orange Red - PromU (Promoter Upstream TSS)
- State 3 - Orange Red - PromD1 (Promoter Downstream TSS with DNase)
- State 4 - Orange Red - PromD2 (Promoter Downstream TSS)
- State 5 - Green - Tx5' (Transcription 5')
- State 6 - Green - Tx (Transcription)
- State 7 - Green - Tx3' (Transcription 3')
- State 8 - Light Green - TxWk (Weak transcription)
- State 9 - GreenYellow - TxReg (Transcription Regulatory)
- State 10 - GreenYellow - TxEnh5' (Transcription 5' Enhancer)
- State 11 - GreenYellow - TxEnh3' (Transcription 3' Enhancer)
- State 12 - GreenYellow - TxEnhW (Transcription Weak Enhancer)
- State 13 - Orange - EnhA1 (Active Enhancer 1)
- State 14 - Orange - EnhA2 (Active Enhancer 2)
- State 15 - Orange - EnhAF (Active Enhancer Flank)
- State 16 - Yellow - EnhW1 (Weak Enhancer 1)
- State 17 - Yellow - EnhW2 (Weak Enhancer 2)
- State 18 - Yellow - EnhAc (Enhancer Acetylation Only)
- State 19 - Light Yellow - DNase (DNase only)
- State 20 - Medium Aquamarine - ZNF/Rpts (ZNF genes & repeats)
- State 21 - PaleTurquoise - Het (Heterochromatin)
- State 22 - Light Purple - PromP (Poised Promoter)
- State 23 - Purple - PromBiv (Bivalent Promoter)
- State 24 - Silver - ReprPC (Repressed PolyComb)
- State 25 - White - Quies (Quiescent/Low)

More details can be found in https://genome.ucsc.edu/cgi-bin/hgTrackUi?hgsid=433891379_Ri32gCKnmDLSQiE1ZgjJAgfHAo6H&c=chr16&g=hub_24125_RoadmapConsolidatedAssaya27004.

The 13 states in ChromHMM

- State 1 - Bright Red - Active Promoter
- State 2 - Light Red -Weak Promoter
- State 3 - Purple - Inactive/poised Promoter
- State 4 - Orange - Strong enhancer
- State 5 - Orange - Strong enhancer
- State 6 - Yellow - Weak/poised enhancer
- State 7 - Yellow - Weak/poised enhancer
- State 8 - Blue - Insulator
- State 9 - Dark Green - Transcriptional transition
- State 10 - Dark Green - Transcriptional elongation
- State 11 - Light Green - Weak transcribed
- State 12 - Gray - Polycomb-repressed
- State 13 - Light Gray - Heterochromatin; low signal
- State 14 - Light Gray - Repetitive/Copy Number Variation
- State 15 - Light Gray - Repetitive/Copy Number Variation

More details can be found in https://genome.ucsc.edu/cgi-bin/hgTrackUi?hgsid=433891379_Ri32gCKnmDLSQiE1ZgjJAgfHAo6H&c=chr2&g=wgEncodeBroadHmm.

Supplementary Figure 6a: Hidden Markov Model analysis for rs6723868 in 2p22.1

127 cell/tissue types

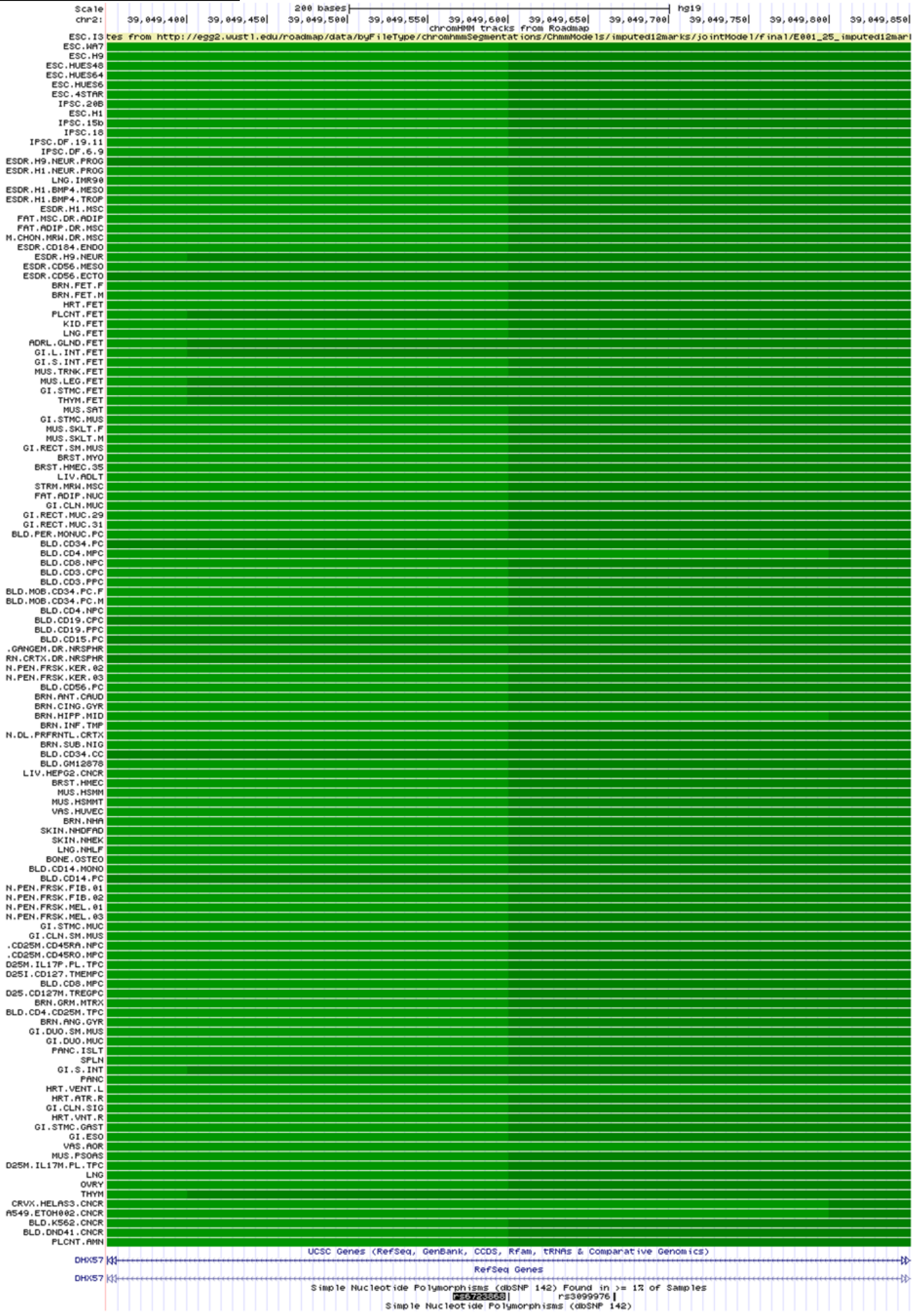

9 cell types

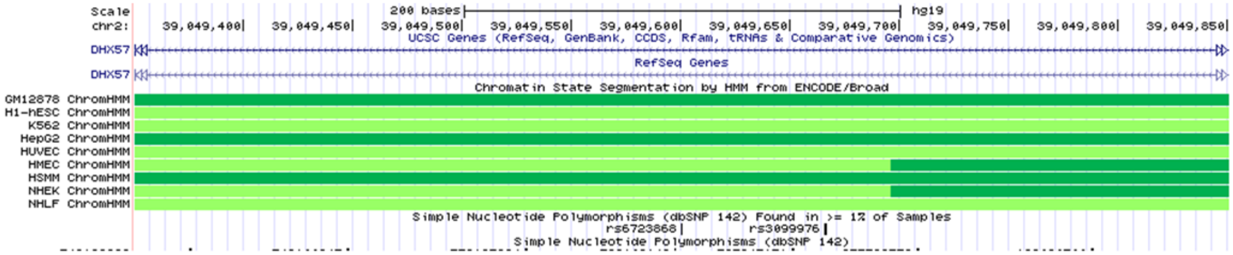

URL: https://genome.ucsc.edu/cgi-bin/hgTracks?db=hg19&position=chr2%3A39049351-39049851&hgsid=433891379_Ri32gCKnmDLSQiE1ZgjJAgfHAo6H

Supplementary Figure 6b: Hidden Markov Model analysis for rs30986 in 16p13.1

<u>127 cell/tissue types</u>

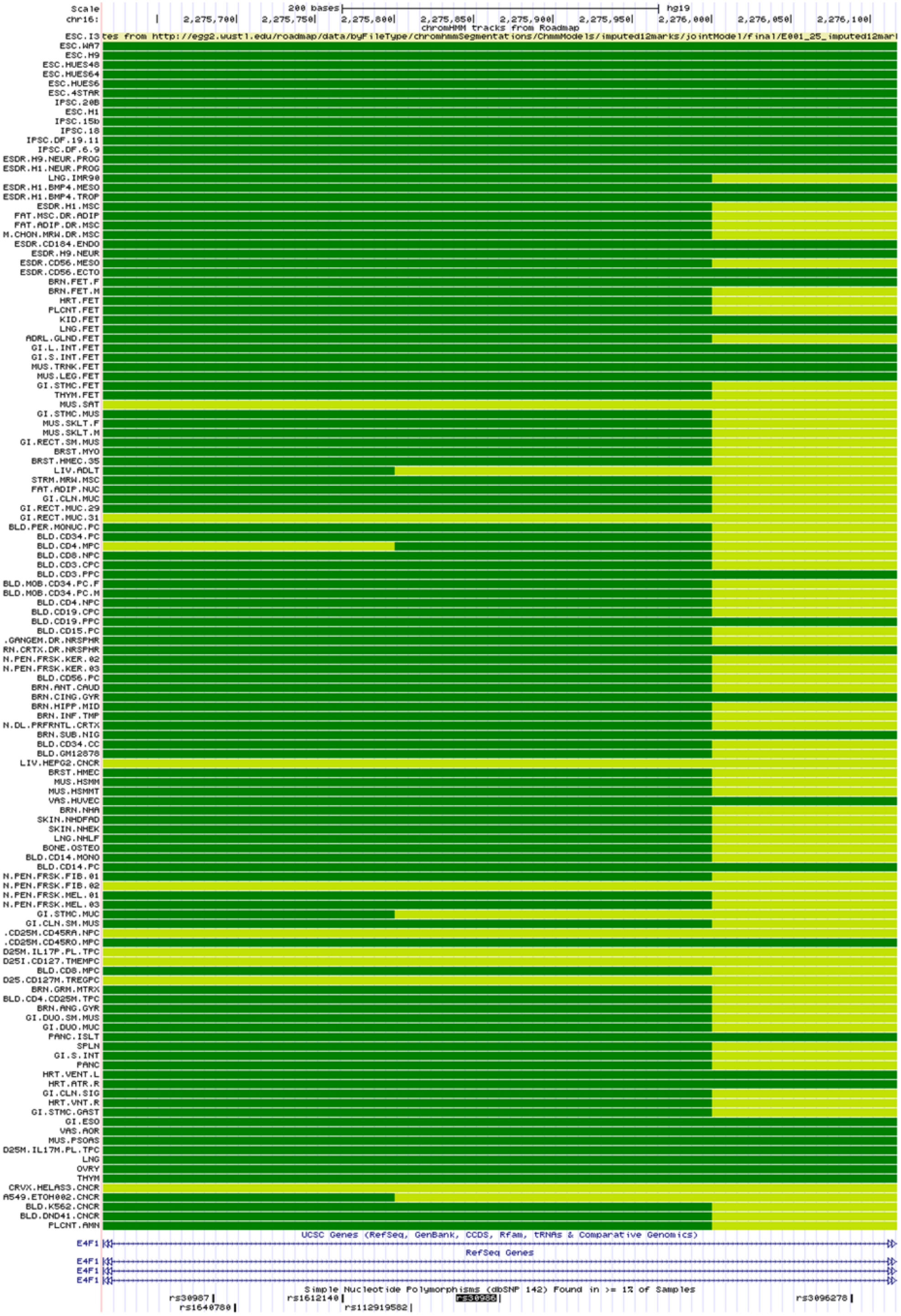

9 cell types

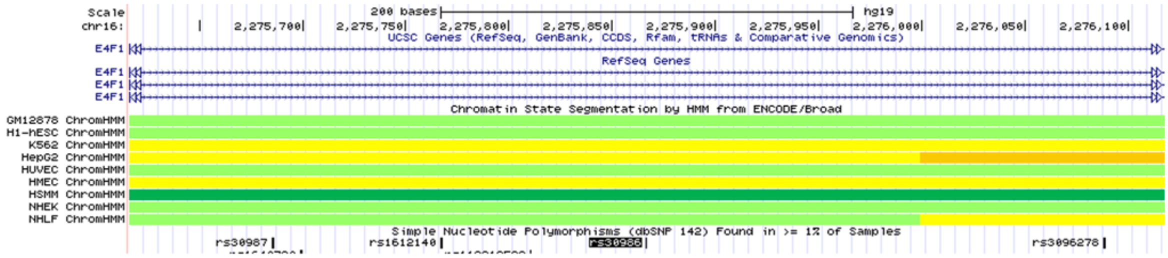

URL: https://genome.ucsc.edu/cgi-bin/hgTracks?db=hg19&position=chr16%3A2275617-2276117&hgsid=433891379_Ri32gCKnmDLSQiE1ZgjJAgfHAo6H

Supplementary Figure 7: Gene expression stratified by SNP genotypes from the UK brain expression database

In the following plots, we present the gene expression levels stratified by SNP genotypes from the Brain eQTL Almanac (BRAINEAC), which is a web-based resource to access the archived brain eQTL results from the UK Brain Expression Consortium (UKBEC) dataset. Whenever possible, we verified that the directionality is congruent (same effect allele) between our study data and the UK datasets. Panel (a) shows the stratification of the gene expression level of *DHX57* (transcript id =2549021 and probe id =2549027) by rs6723868, while panel (b) shows *MLST8* (transcript id = 3466593 and probe id =3644621) by rs30986. The brain eQTL results from BRAINEAC are available for 10 brain regions including cerebellum (CRBL), frontal cortex (FCTX), hippocampus (HIPP), medulla (MEDU), occipital cortex (OCTX), putamen (PUTM), substantia nigra (SNIG), temporal cortex (TCTX), thalamus (THAL) and intralobular white matter (WHMT). In (a), the minor allele (A) is negatively related to the gene expression levels in CRBL and FCTX while it is positively related to the TCTX region. The sign of this association is consistent with our study results (**Figure 2** in the main article). In (b), the minor allele (T) increases the levels of expression in each brain region. This is consistent with our eQTL results for rs30986 - *MLST8:* note that all of our robust correlation estimates are positive in the four brain regions and assorted neurons (**Figure 2** of the main article).

Supplementary Figure 7a: Expression of DHX57 *stratified by rs6723868*

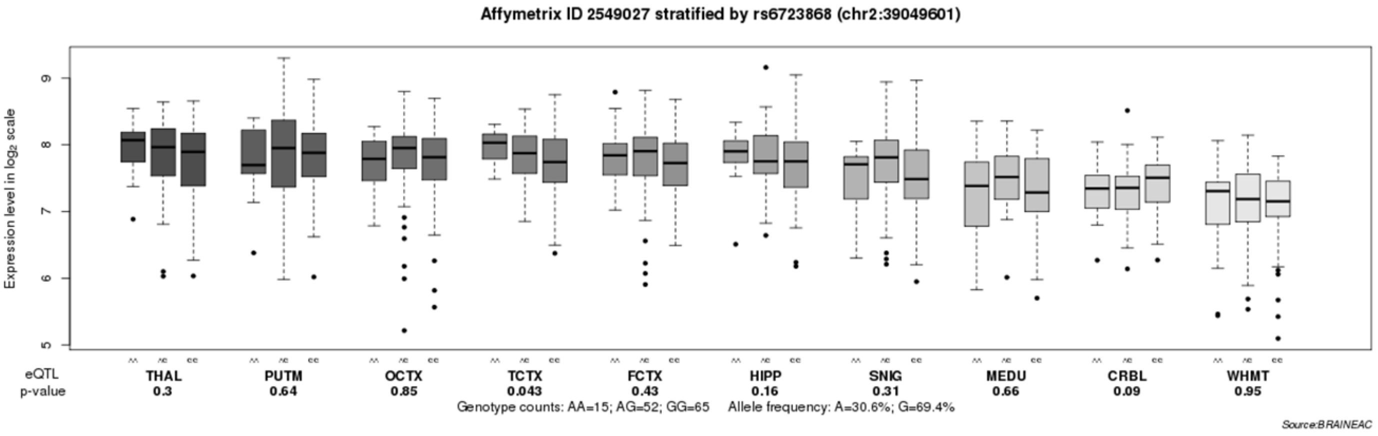

Supplementary Figure 7b: Expression of MLST8 *stratified by rs30986*

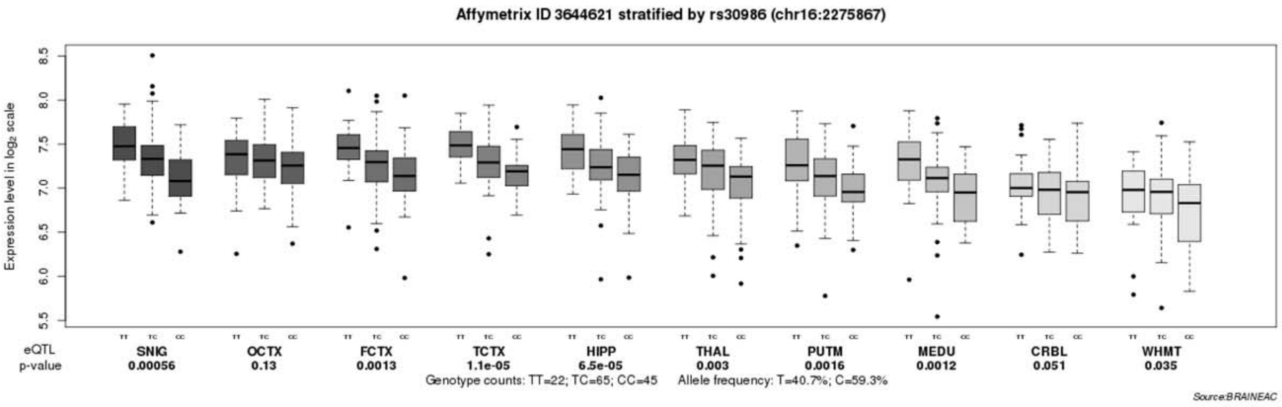

Supplementary Figure 8: Expression levels of cis-acting genes versus chronological age

The scatter plots relate the expression levels of *DHX57*, *MLST8,* and *PGP* to chronological age (x-axis) in all brain data sets for which gene expression levels were available: study 2 (4 brain regions), study 3, study 5 (2 brain regions), and study 6. Each panel reports the results of a robust correlation (biweight midcorrelation estimation).

Supplementary Figure 8a: DHX57

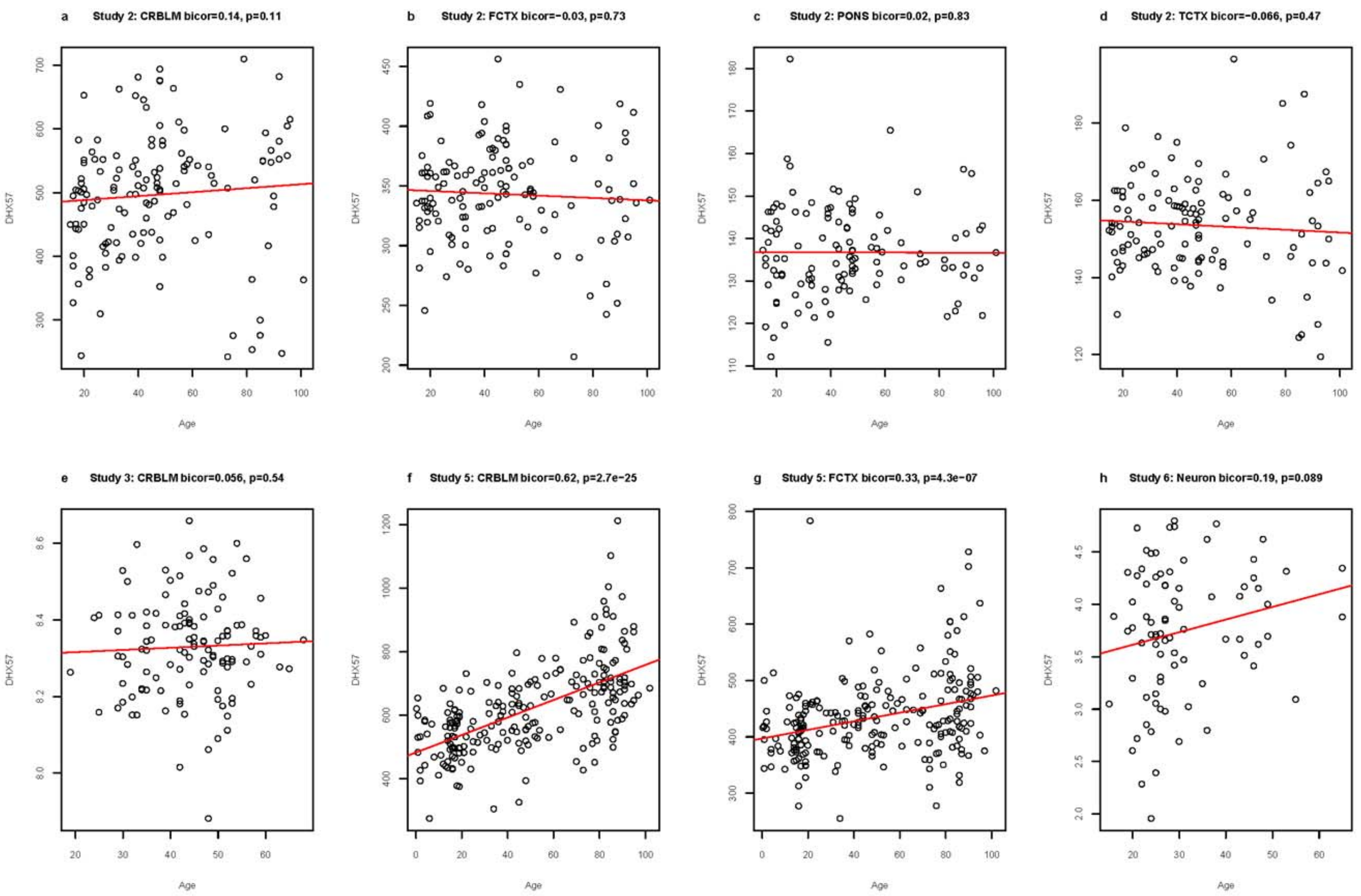

Supplementary Figure 8b: MLST8

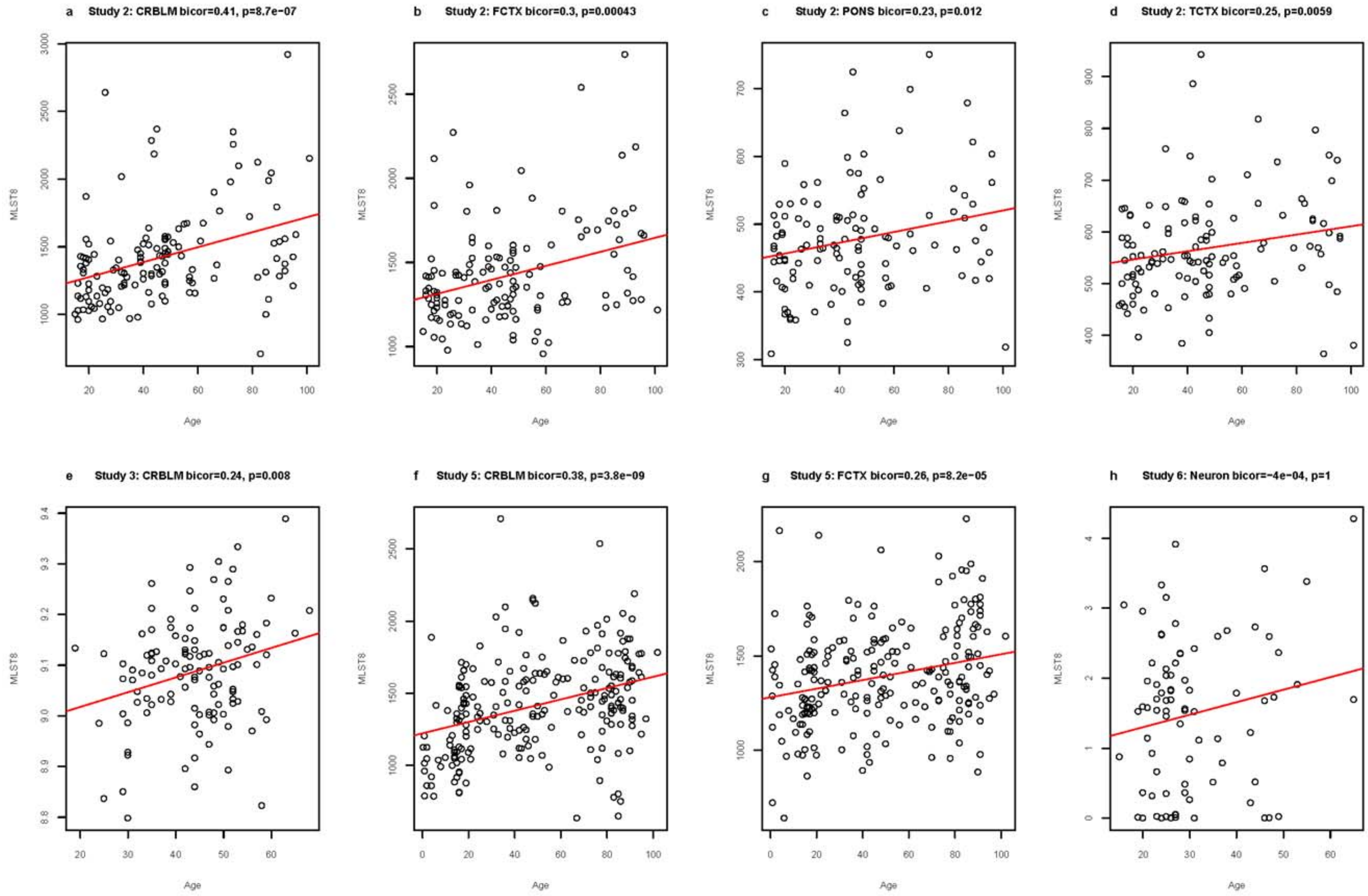

Supplementary Figure 8c: PGP

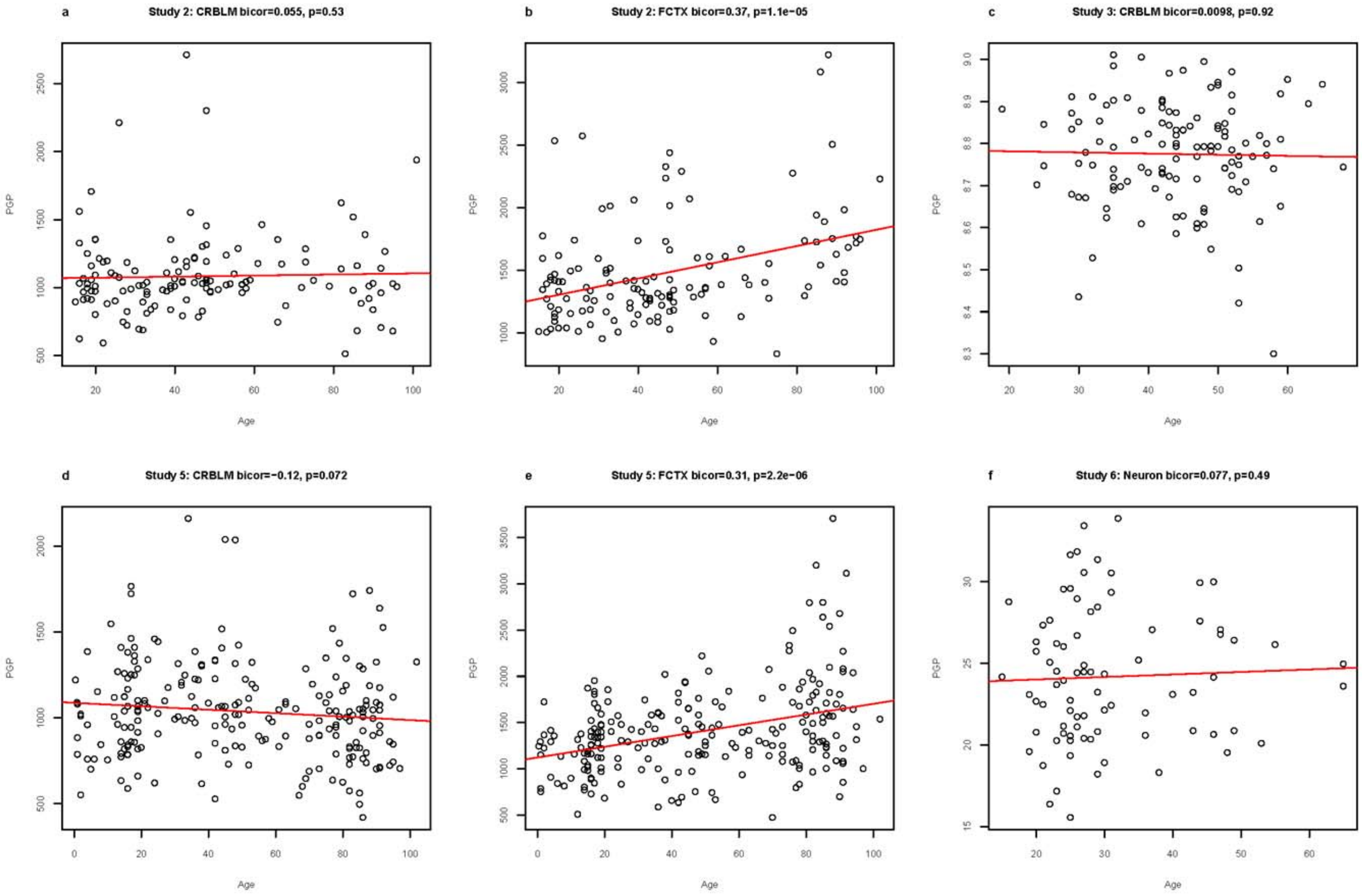

Supplementary Figure 9: Manhattan plot of cognitive decline/slope in the HRS. The plot presents the Manhattan plots for the GWAS performed on all the HRS participants (ALL) as well as the subgroups stratified by ethnicity, including individuals of European (EUR), Africans (AFR) and Americas (AMR). SNPs associated at $P < 5.0\text{x}10^{-8}$ are coded in red color, with their loci (gene names) listed in top.

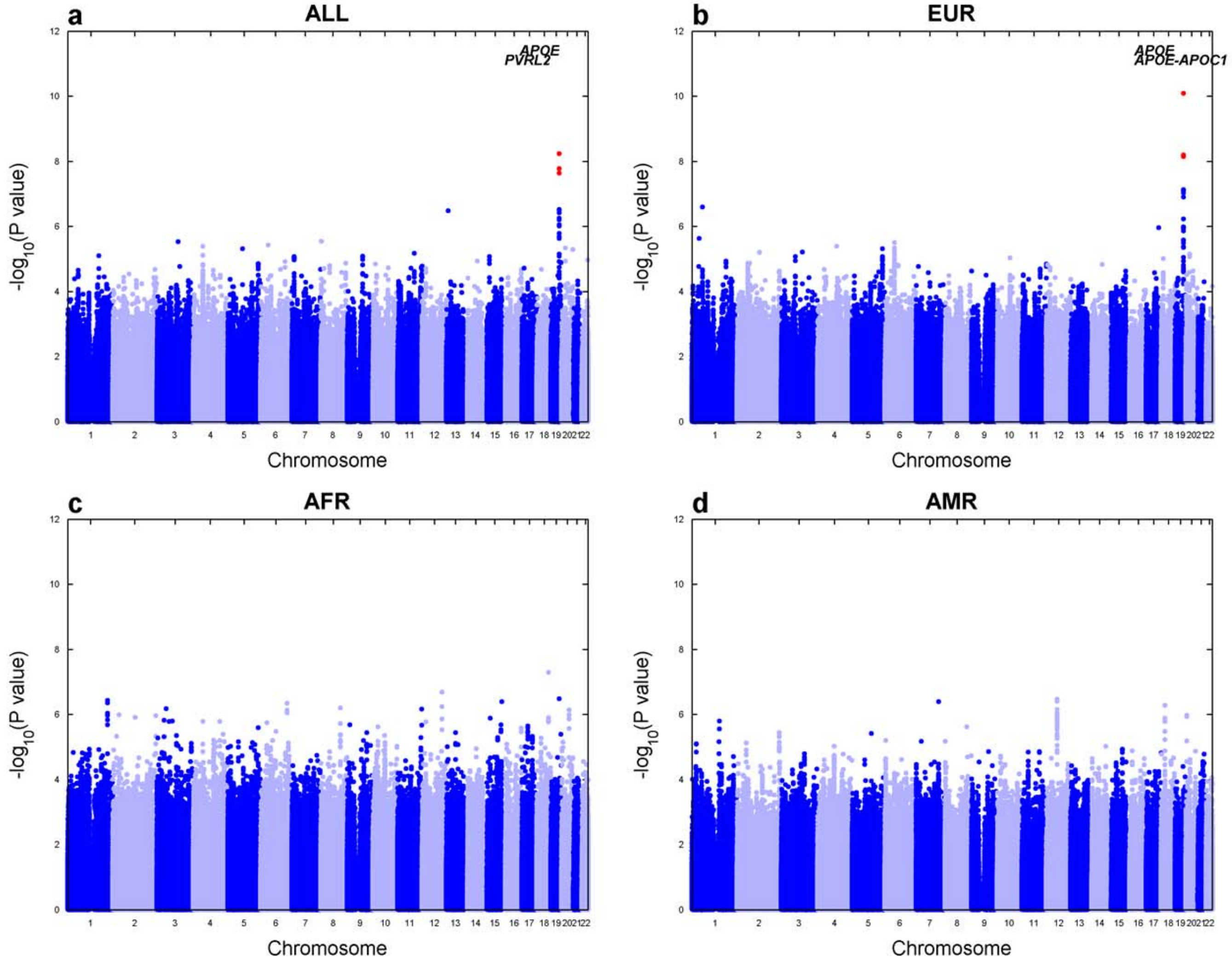

Supplementary Figure 10: Manhattan plot of dementia status for HRS wave 8

The plot presents the Manhattan plots for the GWAS performed on all the HRS participants (ALL) as well as the subgroups stratified by ethnicity, including individuals of European (EUR), Africans (AFR) and Americas (AMR). SNPs associated at $P < 5.0 \times 10^{-8}$ are coded in red color, with their loci (gene names) listed in top.

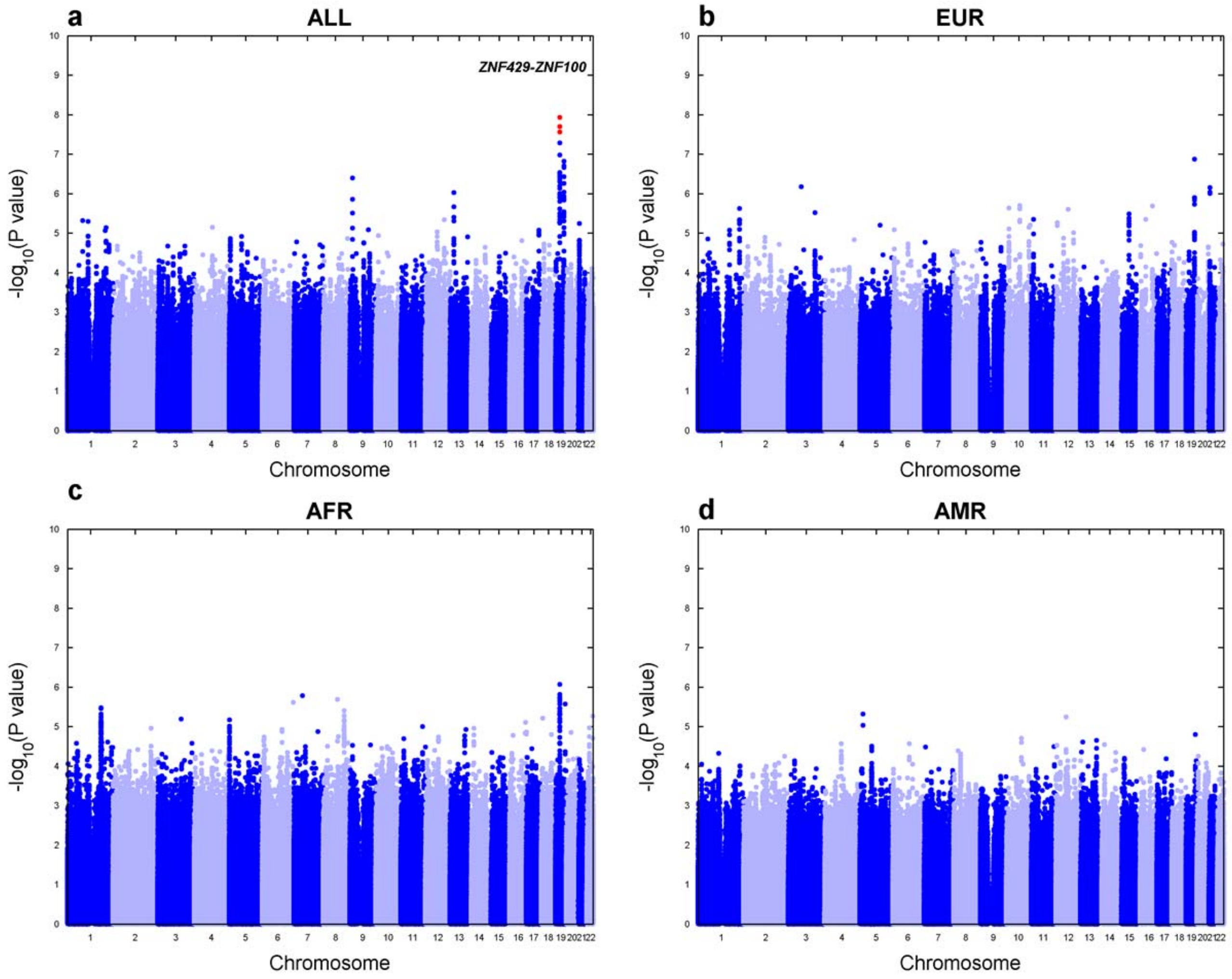

Supplementary Figure 11: Manhattan plot of dementia status for HRS wave 9

The plot presents the Manhattan plots for the GWAS performed on all the HRS participants (ALL) as well as the subgroups stratified by ethnicity, including individuals of European (EUR), Africans (AFR) and Americas (AMR). SNPs associated at $P < 5.0\text{x}10^{-8}$ are coded in red color, with their loci (gene names) listed in top.

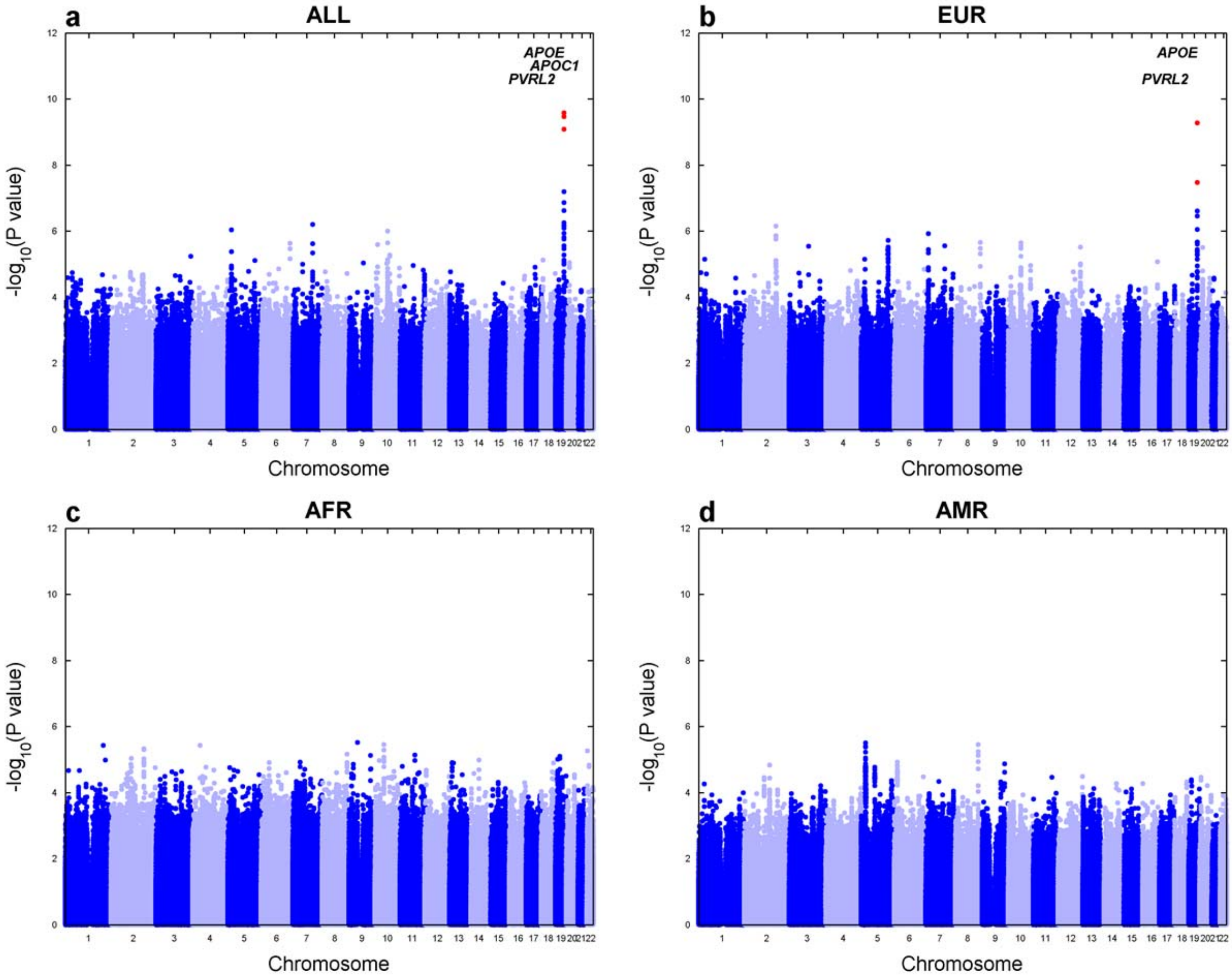

Supplementary Figure 12: Gene expression levels of *DHX57*, *MLST8* and *PGP* in different brain regions. The bar plots depict mean gene expression levels (y-axis) versus brain region (x-axis). The multiple brain regions were collected from the same subjects in study 2 and 5. Each bar plot reports one standard error and the results from a non-parametric group comparison test (Kruskal Wallis).

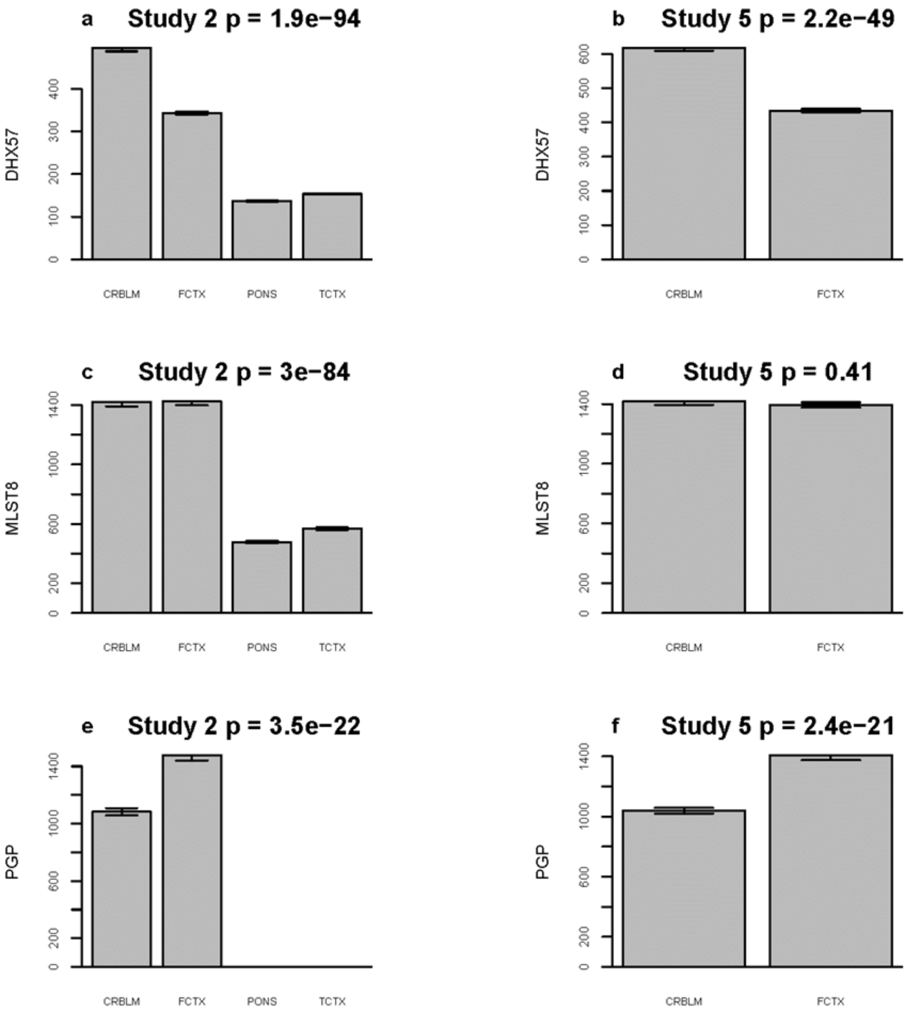

Supplementary Figure 13: Hierarchical clustering of the brain expression data from Study 2. These unsupervised hierachical clustering plots were used to identify outlying samples. The plots present four dendrograms based on mRNA expression profiled from CRBLM, frontal cortex (FCTX), pons (PONS) and temporal cortex (TCTX) tissues respectively. Expression data in CRBLM and FCTX were measured using Illumina HumanHT-12 V3.0 (m=48806) while PONS and TCTX were measured using Illumina humanRef-8 v2.0 (m=22184). The color band underneath the dendrogram represents standardized connectivity measures, $\boldsymbol{Z_k}$, (based on interarray correlation) with outliers colored in red. The outliers were removed from the cis-eQTL analysis.

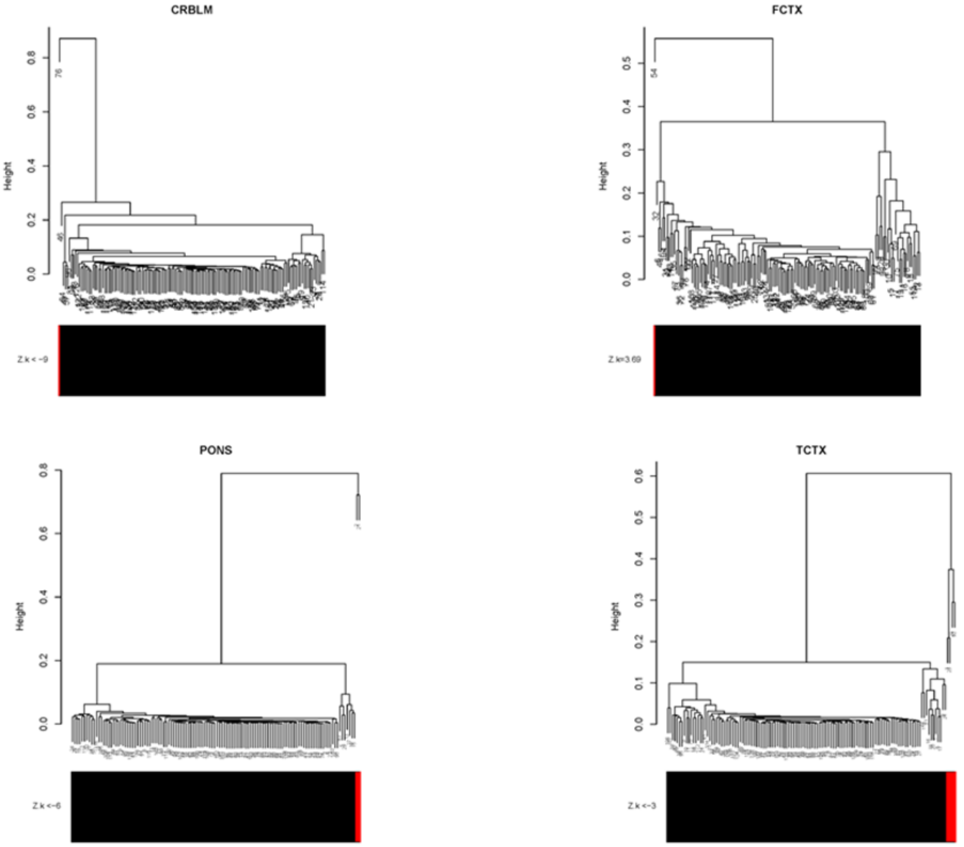

Supplementary Figure 14: Hierarchical clustering of the brain expression data from Study 3

Dendrogram based on mRNA expression profiled from the cerebellum. The color band underneath the dendrogram represents standardized connectivity measures, $\boldsymbol{Z_k}$, with outliers colored in red. The outliers were removed from the cis-eQTL analysis.

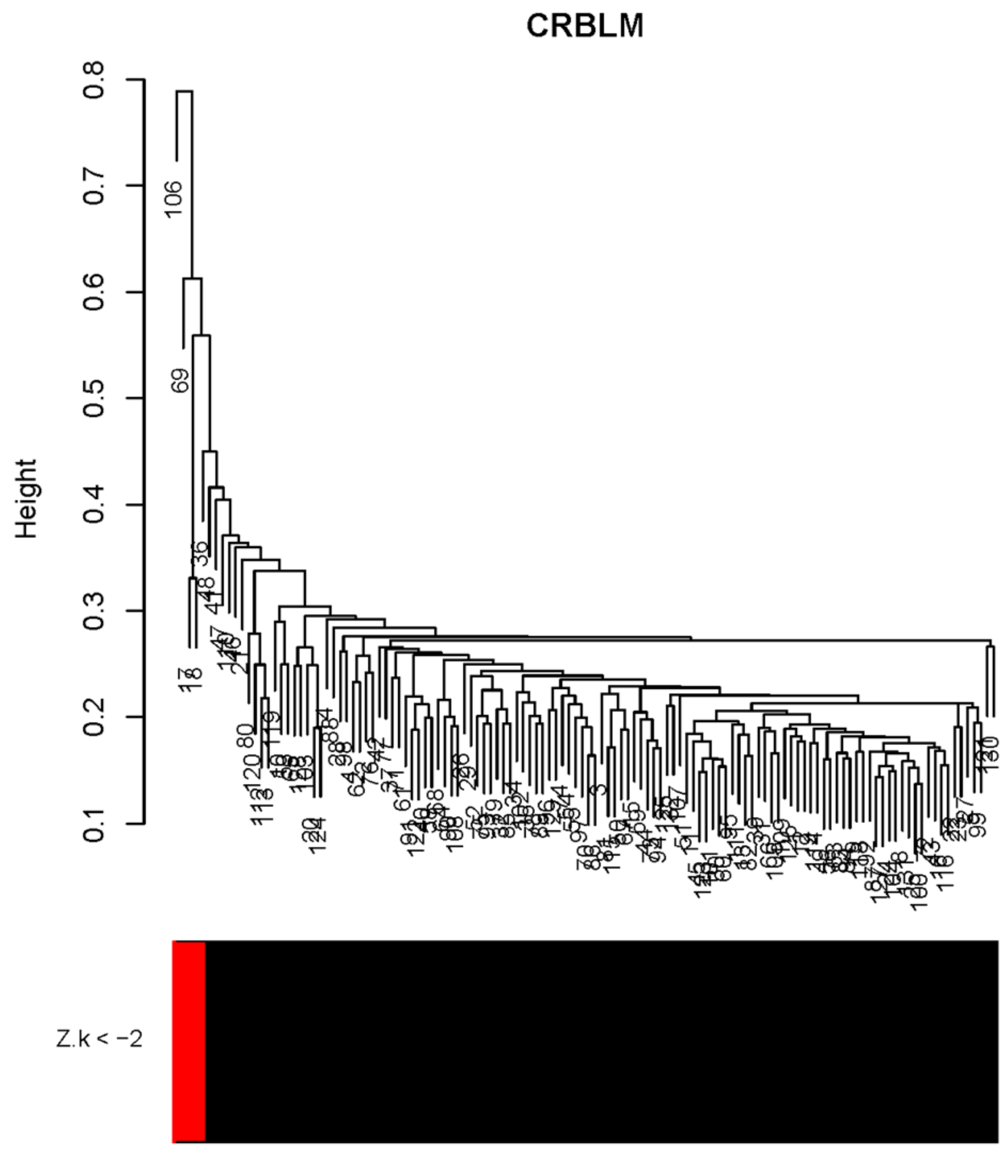

Supplementary Figure 15: Hierarchical clustering of the brain expression data from Study 5

The plots present dendrograms based on mRNA expression profiled from CRBLM (in the top) and FCTX (in the bottom), respectively. The color band underneath the dendrogram represents standardized connectivity measures, $\boldsymbol{Z_k}$, with outliers colored in red.

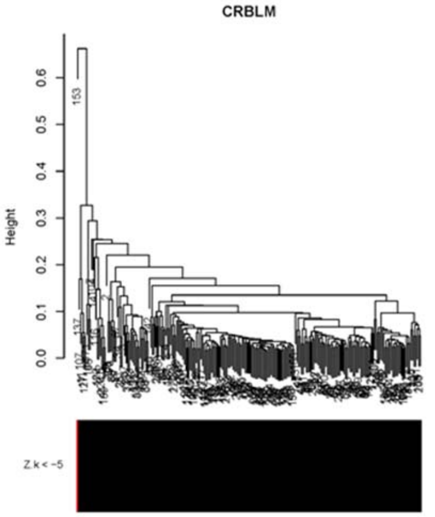

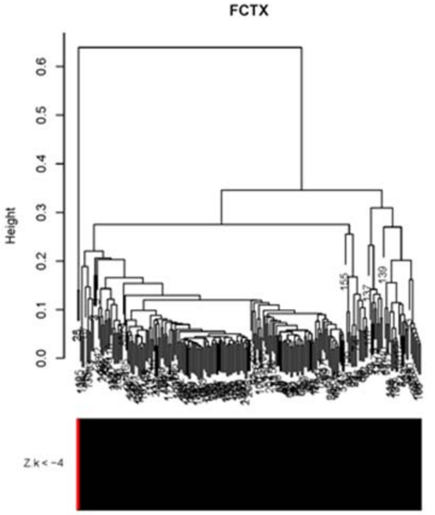

Supplementary Figure 16: Hierarchical clustering of the neuron expression data (Study 6)

The plot presents a dendrogram based on mRNA expression profiled from sorted neurons. The color band underneath the dendrogram represents standardized connectivity measures, $\boldsymbol{Z_k}$, with outliers colored in red.

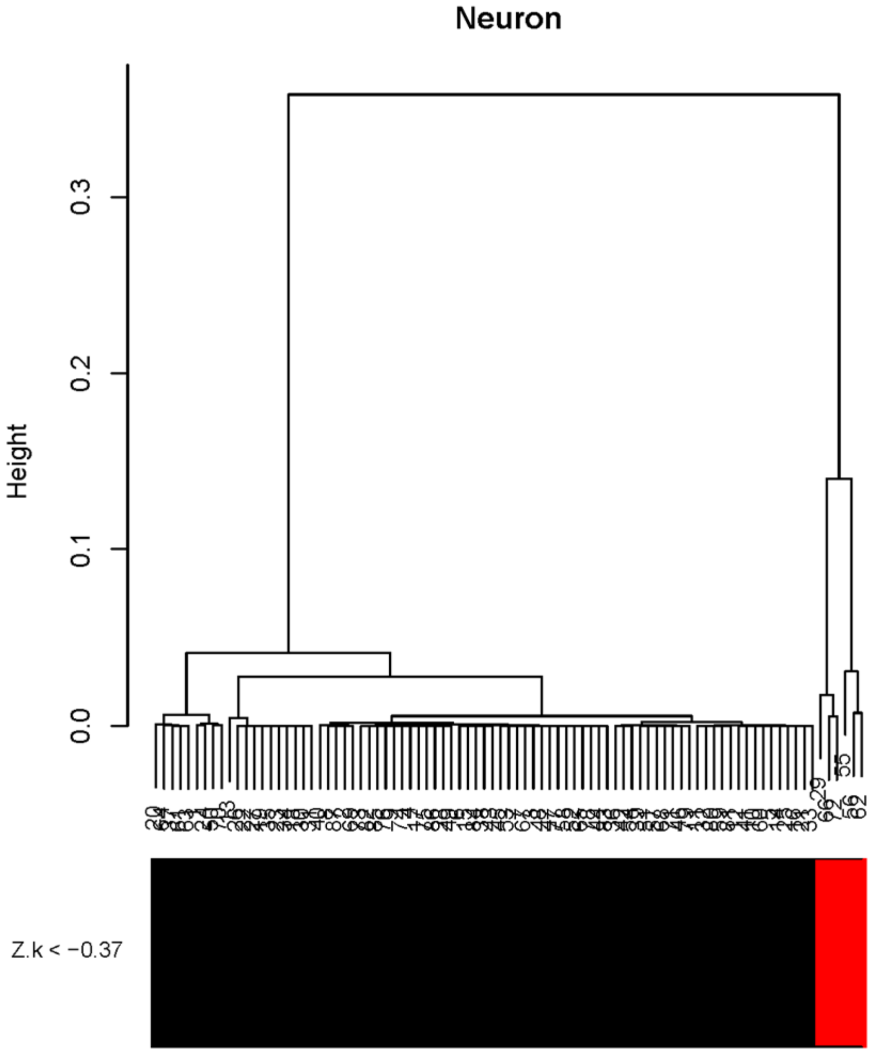

Supplementary Tables

Supplementary Table 1: Genomic platforms in the different studies

Data	SNP platform	DNA methylation platform	Gene expression platform
**Study 1**	Illumina 610-Quad, Illumina 666W-Quad	Illumina 450K	Not available
**Study 2**	Illumina HumanHap550v3	Illumina 27	Illumina humanRef-8 v2.0 HumanHT-12 V3.0
**Study 3**	Affymetrix SNP Array 5.0	Illumina 27	Affymetrix Human Gene 1.0 ST Array
**Study 4**	Illumina HumanOmniExpress	Illumina 450K	Not available
**Study 5**	Illumina 610-Quad	Illumina 27	Illumina HumanHT-12 V3.0
**Study 6***	Illumina HumanOmniExpress Exome-8v1	Not Applicable	Illumina HiSeq2000

*only used in brain *cis*-eQTL analysis

Supplementary Table 2: SNPs used for imputation and GWAS

QC prior imputation

Data	# SNPs	MAF	HWE *P*	Missing rate threshold[a]	Genotyping rate
**Study 1**	528739	≥ 1%	>1.0 x 10^{-4}	3% (MAF > 5%)<br>1% (MAF > 1%)	>.97
**Study 2**	495169	≥ 5%	>1.0 x 10^{-4}	0%	>.99
**Study 3**	368899	≥ 5%	>1.0 x 10^{-4}	< 30%	.97
**Study 4**	601993	≥ 5%	>1.0 x 10^{-4}	<30%	>.99
**Study 5**	515214	≥ 5%	>1.0 x 10^{-4}	<10%	>.99
**Study 6***	576923	≥ 5 copies minor allele	>1.0 x 10^{-6}	< 10%	>.99

QC post imputation and GWAS

Data	#SNPs	Imputation software	Info[b]	genotype method[c]	Min #[d]	MAF	# PCA	GWAS software	λ_{GC}
**Study 1**	5552013	SHAPEIT/ IMPUTE2	0.4	Threshold at 0.80	20	≥ 5%	0	Plink	0.98
**Study 2**	5546698	SHAPEIT/ IMPUTE2	0.4	Threshold at 0.90	30	≥ 5%	2	*R*	0.97
**Study 3**	6234466	SHAPEIT/ IMPUTE2	0.4	Threshold at 0.90	30	≥ 5%	2	*R*	0.99
**Study 4**	5780451	SHAPEIT/ IMPUTE2	0.4	Expected dosage	NA	≥ 5%	0	*R*	1.01
**Study 5**	6473646	SHAPEIT/ IMPUTE2	0.4	Expected dosage	NA	≥ 5%	0	*R*	1.00
**Study 6***	NA	MaCH/ Minimach	0.3	Expected dosage	NA	NA	NA	*R*	NA

*only used in brain *cis*-eQTL analysis

Abbreviations: HWE=Hardy-Weinberg equilibrium; MAF= minor allele frequency; NA=not applicable; PCA= principal component; λ_{GC} = genomic inflation.

[a]SNPs failing the threshold were removed prior imputation.

[b]Assessment for imputed SNPs by info measure in IMPUTE2 and R^2 in MaCH.

[c]HWE *P* > 1.0 x 10^{-4} was required for thresholded genotypes.

[d]Minimum number of samples per marker level was required for conduction association analysis, applied to SNPs with thresholded genotypes.

Supplementary Table 3: Conditional analysis of epigenetic age acceleration for SNPs near 16p13.3

To test whether the region 16p13.3 might harbor two independent loci that relate to the epigenetic age of the cerebellum, we repeated our association analysis for 3 SNPs in that region after conditioning on rs30986. Note that the three SNPs are close to rs30986 in terms of physical distance and linkage disequilibrium (LD), as displayed in the columns "Distance" and "LD", respectively. The last two columns report the meta-analysis results after conditioning on rs30986. In study 1, conditional analysis was only performed on the SNP rs27648 because the other two SNPs were in perfect LD with rs30986. Similarly, we were not able to conduct conditional analysis for studies 2 or 3 since each of the three SNPs was in perfect LD with rs30986 in these studies.

SNP	Position (bp)	Minor/ Major alleles	Distance (kp)	LD[a]	Corr. (SE)	Meta *P*
rs27709	2281829	A/G	6.0	0.98	0.01 (0.07)	0.9
rs26840	2285357	T/C	9.5	0.91	0.04 (0.06)	0.5
rs27648	2291350	A/G	15.5	0.86	0.06 (0.06)	0.4

[a] Calculated using the 1000 genome individuals with ancestry of European released in December 2013.

Supplementary Table 4: *Cis*-expression QTL analysis for SNPs associated with epigenetic age acceleration in the cerebellum

For studies 2, 3, 5 we also had gene expression data available which allowed us to correlate our five genome-wide significant SNPs with cerebellar gene expression levels of adjacent genes, i.e. genes that are located within +/- 500 kb of these five SNPs). Each of the five SNP corresponds to a different set of adjacent genes which gave rise to five lists of gene transcripts (labelled a-e). a) *cis*-eQTL results for SNP, rs6723868 on 2p22.1 b-e) results for rs30986, rs27709, rs26840, and rs27648 in 16p13.3, respectively. The table reports the gene symbol, gene identifier, and results from the correlation tests. The column "meta.bicor" denotes the meta-analysis estimate of the correlation coefficient between the SNP and the gene transcript across the three studies. Similar, "Meta.se" denotes the standard error, and "Meta.p" the meta-analysis p-value. "bicor.Study2" denotes the robust correlation coefficient (biweight midcorrelation) in study 2. "n.Study2" denotes the number of non-missing observations in study 2.

a) Test SNP=rs6723868 and test allele=G

CHR	Gene	GeneID	Meta.bicor	Meta.se	Meta.p	same_direction_for_all_bicors	bicor.Study2	bicor.Study3	bicor.Study5	ID.Study2	p.Study2	n.Study2	ID.Study3	p.Study3	n.Study3	ID.Study5	p.Study5	n.Study5	num.data	dist_to_SNP (bp)
2	DHX57	90957	0.204747	0.046895	1.26E-05	Yes	0.0813302	0.412915	0.175341	ILMN_1732226	0.357638	130	X8051637	1.96E-05	100	ILMN_1732226	0.009319	219	3	0
2	GEMIN6	79833	0.010236	0.047562	0.829607	No	-0.0654888	0.027577	0.047073	ILMN_1707484	0.459136	130	X8041553	0.785346	100	ILMN_1707484	0.488298	219	3	40495

b) Test SNP=rs30986 and test allele=C

CHR	Gene	GeneID	Meta.bicor	Meta.se	Meta.p	same_direction_for_all_bicors	bicor.Study2	bicor.Study3	bicor.Study5	ID.Study2	p.Study2	n.Study2	ID.Study3	p.Study3	n.Study3	ID.Study5	p.Study5	n.Study5	num.data	dist_to_SNP (bp)
16	E4F1	1877	-0.05671	0.050494	0.26139	Yes	-0.0665137	-0.09917	-0.04066	ILMN_1720287	0.462962	124	X7992568	0.467128	56	ILMN_1720287	0.549449	219	3	0
16	DNASE1L2	1775	0.017437	0.050498	0.729867	No	-0.010048	-0.0939	0.06042	ILMN_1801366	0.911807	124	X7992584	0.491225	56	ILMN_1801366	0.373553	219	3	10600
16	PGP	283871	0.199654	0.049787	6.07E-05	No	0.3397487	-0.10024	0.19076	ILMN_2057566	0.000113	124	X7998759	0.462317	56	ILMN_2057566	0.004613	219	3	11045
16	ECI1	1632	-0.00219	0.050526	0.965446	No	0.0343658	0.034592	-0.0318	ILMN_2049303	0.704749	124	X7998762	0.800191	56	ILMN_2049303	0.639817	219	3	14005
16	C16orf79	283870	0.029578	0.050475	0.55788	No	0.1027406	-0.10559	0.021894	ILMN_1759411	0.256169	124	X7998749	0.438622	56	ILMN_1759411	0.747312	219	3	14798
16	MLST8	64223	-0.20268	0.0499	4.87E-05	Yes	-0.1047093	-0.39619	-0.20587	ILMN_1789240	0.247122	124	X7992552	0.002505	56	ILMN_1789240	0.002198	219	3	16449
16	RNPS1	10921	0.069678	0.050455	0.167282	Yes	0.1240879	0.096875	0.032172	ILMN_1691843	0.16972	124	X7998774	0.47754	56	ILMN_1691843	0.635854	219	3	27232
16	CASKIN1	57524	-0.01198	0.05049	0.812474	No	-0.0625122	-0.11241	0.041369	ILMN_1679039	0.490363	124	X7998724	0.409456	56	ILMN_1679039	0.542545	219	3	29402
16	TRAF7	84231	-0.00718	0.050478	0.886832	No	-0.0556359	-0.13267	0.051261	ILMN_2405991	0.539389	124	X7992529	0.329678	56	ILMN_2405991	0.450399	219	3	47737
16	ABCA3	21	0.017306	0.050511	0.731885	No	0.0423123	-0.09276	0.030571	ILMN_1758523	0.640781	124	X7998784	0.496531	56	ILMN_1758523	0.652762	219	3	50011
16	SNORD60	26788	0.0232	NA	0.865232	NA	NA	0.0232	NA	NA	NA	NA	X7998722	0.865232	56	NA	NA	NA	1	70761
16	RAB26	25837	0.059859	0.050445	0.235377	Yes	0.1517919	0.015044	0.018746	ILMN_1790317	0.092388	124	X7992518	0.912375	56	ILMN_1790317	0.782664	219	3	71726
16	PKD1	5310	0.001518	0.050464	0.976002	No	0.1129473	-0.09423	-0.03762	ILMN_1669645	0.211668	124	X7999614	0.48971	56	ILMN_2339028	0.579714	219	3	89968
16	TSC2	7249	-0.01321	0.050336	0.792926	No	0.1725258	-0.12792	-0.09014	ILMN_1714216	0.055352	124	X7992474	0.34747	56	ILMN_1714216	0.183835	219	3	137154
16	NTHL1	4913	0.008719	0.050475	0.862853	No	0.0824592	-0.14589	0.005815	ILMN_2227968	0.36257	124	X7998692	0.283338	56	ILMN_2227968	0.931817	219	3	178000
16	SLC9A3R2	9351	-0.14099	0.050222	0.004995	Yes	-0.0476494	-0.2625	-0.16204	ILMN_1765833	0.599217	124	X7992463	0.050645	56	ILMN_1765833	0.016389	219	3	186840
16	CCNF	899	0.014728	0.050533	0.770709	Yes	0.0415385	0.005174	0.002029	ILMN_1773119	0.646905	124	X7992594	0.969812	56	ILMN_1773119	0.976179	219	3	203527
16	NPW	283869	-0.1883	NA	0.164597	NA	NA	-0.1883	NA	NA	NA	NA	X7992460	0.164597	56	NA	NA	NA	1	205111
16	ZNF598	90850	-0.04439	0.050454	0.378972	No	0.0387852	-0.17874	-0.05734	ILMN_2075818	0.668884	124	X7998679	0.18749	56	ILMN_2075820	0.398472	219	3	216104
16	SYNGR3	9143	0.019155	0.050504	0.704477	No	-0.03116	-0.0456	0.063337	ILMN_1786379	0.731183	124	X7992447	0.738588	56	ILMN_1786379	0.350885	219	3	231591
16	C16orf59	80178	-0.00607	0.050503	0.90428	No	0.0650742	-0.09575	-0.02382	ILMN_1656452	0.472721	124	X7992614	0.482704	56	ILMN_1656452	0.725952	219	3	234247
16	GFER	2671	0.084084	0.050436	0.095484	Yes	0.0382124	0.071347	0.112817	ILMN_1768055	0.673493	124	X7992439	0.601295	56	ILMN_1768055	0.095851	219	3	238117
16	NOXO1	124056	0.057742	0.050402	0.251947	No	0.0988154	-0.15872	0.088635	ILMN_1708337	0.27488	124	X7998668	0.242663	56	ILMN_1708337	0.191301	219	3	244427
16	NTN3	4917	-0.0627	0.050405	0.213543	No	-0.1250352	-0.20201	0.007711	ILMN_1656040	0.16646	124	X7992632	0.135419	56	ILMN_1656040	0.909661	219	3	245632
16	TBL3	10607	-0.0416	0.050423	0.409307	No	0.0873521	-0.08812	-0.10239	ILMN_2177413	0.334692	124	X7992414	0.518376	56	ILMN_2177413	0.130904	219	3	247116
16	TBC1D24	57465	0.013975	0.050499	0.781977	No	0.0196444	-0.12107	0.044382	ILMN_1780197	0.828554	124	X7992639	0.3741	56	ILMN_2060212	0.513523	219	3	249279
16	RNF151	146310	0.012002	0.050459	0.811993	No	-0.0769227	-0.07363	0.083029	ILMN_1745330	0.395793	124	X7992409	0.58967	56	ILMN_1745330	0.22103	219	3	256891
16	RPS2	6187	-0.03008	0.050426	0.550878	No	0.0407636	-0.24194	-0.01591	ILMN_2218277	0.653061	124	X8013348	0.072424	56	ILMN_2218277	0.814847	219	3	261040
16	SNORA64	26784	-0.00814	0.050522	0.872072	No	0.0453373	-0.02867	-0.03309	ILMN_1709604	0.617076	124	X7998666	0.833869	56	ILMN_1709604	0.626293	219	3	262760
16	SNORA10	574042	0.076108	0.050459	0.131477	Yes	0.0983725	0.02198	0.076929	ILMN_2189842	0.277048	124	X7998664	0.872257	56	ILMN_2189842	0.256959	219	3	263400
16	NDUFB10	4716	0.019707	0.050514	0.696448	No	0.0763526	-0.03675	0.001785	ILMN_1811754	0.399314	124	X7992402	0.788001	56	ILMN_1811754	0.979046	219	3	263891
16	RPL3L	6123	0.010111	0.050504	0.841327	No	0.0642948	-0.10366	0.007907	ILMN_1775175	0.478051	124	X7998643	0.447089	56	ILMN_1775175	0.907375	219	3	271188
16	SEPX1	51734	-0.0376	0.050429	0.455946	No	0.0888927	-0.072	-0.10003	ILMN_1719661	0.326205	124	X7998637	0.597978	56	ILMN_1719661	0.140075	219	3	282573
16	ATP6V0C	527	0.040232	0.050312	0.423914	No	-0.1123497	-0.07748	0.154207	ILMN_1773849	0.214111	124	X7992646	0.570299	56	ILMN_1789005	0.02245	219	3	287859
16	AMDHD2	51005	-0.07412	0.05045	0.141805	No	-0.1148324	0.014585	-0.07304	ILMN_1695759	0.204094	124	X7992656	0.915037	56	ILMN_1695759	0.281868	219	3	294495
16	CEMP1	752014	0.06556	0.050365	0.193026	No	0.0945982	-0.1765	0.109693	ILMN_2280816	0.295987	124	X7998817	0.193176	56	ILMN_2280816	0.105468	219	3	304168
16	HS3ST6	64711	-0.01721	0.050363	0.732578	No	-0.1365501	-0.16758	0.087685	ILMN_1710416	0.13047	124	X7998634	0.21702	56	ILMN_1710416	0.196121	219	3	307636
16	PDPK1	5170	0.005343	0.05036	0.915504	No	-0.137814	-0.09978	0.111893	ILMN_1653793	0.126915	124	X7998820	0.464369	56	ILMN_1773758	0.098621	219	3	312102
16	C16orf73	254528	-0.04497	0.050431	0.372517	No	0.0757521	-0.09571	-0.10005	ILMN_1754241	0.403043	124	X7998615	0.482865	56	ILMN_1754241	0.140007	219	3	353688
16	LOC65227	652276	-0.21487	NA	0.111761	NA	NA	-0.21487	NA	NA	NA	NA	X7992678	0.111761	56	NA	NA	NA	1	377517
16	FAHD1	81889	0.01489	0.050407	0.767689	No	-0.133949	0.049037	0.090419	ILMN_1701457	0.138027	124	X7992396	0.719669	56	ILMN_1694593	0.182481	219	3	385664
16	HAGH	3029	-0.03104	0.050453	0.538403	No	-0.0851622	-0.16917	0.03404	ILMN_2379520	0.346996	124	X7998604	0.212621	56	ILMN_2379520	0.616365	219	3	398672
16	FLJ42627	645644	-0.08121	0.054219	0.134189	NA	-0.1841035	NA	-0.02248	ILMN_1792882	0.040672	124	NA	NA	NA	ILMN_1792882	0.740819	219	2	413115
16	IGFALS	3483	-0.00555	0.050516	0.912469	No	0.0565838	-0.01017	-0.03931	ILMN_2188966	0.53249	124	X7998601	0.940717	56	ILMN_2188966	0.5628	219	3	430958
16	NUBP2	10101	0.028964	0.050454	0.565925	No	0.1413241	-0.05567	-0.01378	ILMN_1657237	0.117431	124	X7992388	0.683644	56	ILMN_1657237	0.8393	219	3	436675
16	SPSB3	90864	-0.00588	0.050457	0.907192	No	-0.0437532	-0.17193	0.056943	ILMN_1682864	0.629443	124	X7998590	0.205138	56	ILMN_1682864	0.401729	219	3	443286
16	EME2	197342	0.047843	0.050486	0.343307	No	-0.0165424	0.058162	0.081317	ILMN_1771185	0.855304	124	X7992379	0.670264	56	ILMN_1771185	0.230732	219	3	449628
16	MRPS34	65993	-0.11774	0.050301	0.019249	Yes	-0.2059029	-0.16343	-0.05583	ILMN_2210482	0.021776	124	X7998585	0.228781	56	ILMN_2210482	0.410986	219	3	452727
16	NME3	4832	0.079416	0.050438	0.115361	Yes	0.1264848	0.123643	0.041806	ILMN_1669456	0.161561	124	X7998577	0.363939	56	ILMN_1669456	0.538287	219	3	454157
16	MAPK8IP3	23162	-0.01775	0.050509	0.725202	No	0.0505665	-0.04689	-0.0489	ILMN_1811574	0.577023	124	X7992347	0.731482	56	ILMN_1811574	0.47155	219	3	455549
16	KCTD5	54442	-0.04439	0.050398	0.378471	No	-0.1582267	0.144982	-0.02699	ILMN_1652331	0.079231	124	X7992685	0.28637	56	ILMN_1652331	0.691234	219	3	456627
16	PRSS27	83886	-0.02406	0.050464	0.633568	No	0.0399807	-0.19472	-0.01703	ILMN_1775549	0.659305	124	X7998843	0.150409	56	ILMN_1775549	0.802144	219	3	486555

c) Test SNP= 27709 and test allele=G

CHR	Gene	GeneID	Meta.bicor	Meta.se	Meta.p	same_direction_for_all_bicors	bicor.Study2	bicor.Study3	bicor.Study5	ID.Study2	p.Study2	n.Study2	ID.Study3	p.Study3	n.Study3	ID.Study5	p.Study5	n.Study5	num.data	dist_to_SNP (bp)
16	E4F1	1877	-0.03794	0.05045	0.452025	Yes	-0.0248278	-0.0981	-0.03043	ILMN_1720287	0.783441	125	X7992568	0.471961	56	ILMN_1720287	0.654299	219	3	0
16	DNASE1L2	1775	0.015623	0.05045	0.756805	No	0.0227142	-0.0882	0.037353	ILMN_1801366	0.801479	125	X7992584	0.518011	56	ILMN_1801366	0.582456	219	3	4638
16	ECI1	1632	0.00512	0.050468	0.919201	No	0.0372176	0.007823	-0.01372	ILMN_2049303	0.68029	125	X7998762	0.954369	56	ILMN_2049303	0.839993	219	3	8043
16	PGP	283871	0.194304	0.049776	9.48E-05	No	0.3273435	-0.09023	0.185436	ILMN_2057566	0.000194	125	X7998759	0.508403	56	ILMN_2057566	0.005916	219	3	17007
16	C16orf79	283870	0.026986	0.050366	0.592096	No	0.1293937	-0.15326	0.013627	ILMN_1759411	0.150382	125	X7998749	0.25945	56	ILMN_1759411	0.841073	219	3	20760
16	RNPS1	10921	0.061608	0.050411	0.221659	Yes	0.0988882	0.107408	0.02904	ILMN_1691843	0.272559	125	X7998774	0.430743	56	ILMN_1691843	0.669097	219	3	21270
16	MLST8	64223	-0.21607	0.0498	1.43E-05	Yes	-0.123067	-0.36046	-0.23047	ILMN_1789240	0.171528	125	X7992552	0.006353	56	ILMN_1789240	0.000587	219	3	22411
16	CASKIN1	57524	-0.02641	0.050425	0.600421	No	-0.0980669	-0.06735	0.024453	ILMN_1679039	0.276581	125	X7998724	0.621864	56	ILMN_1679039	0.71896	219	3	35364
16	ABCA3	21	0.005687	0.050454	0.910252	No	0.0348329	-0.09275	0.013616	ILMN_1758523	0.699757	125	X7998784	0.496587	56	ILMN_1758523	0.841203	219	3	44049
16	TRAF7	84231	-0.00936	0.050395	0.8526	No	-0.0612079	-0.1565	0.056733	ILMN_2405991	0.497717	125	X7992529	0.249397	56	ILMN_2405991	0.403469	219	3	53699
16	SNORD60	26788	0.003295	NA	0.980773	NA	NA	0.003295	NA	NA	NA	NA	X7998722	0.980773	56	NA	NA	NA	1	76723
16	RAB26	25837	0.047516	0.050359	0.345403	No	0.1689203	0.015047	-0.01415	ILMN_1790317	0.059677	125	X7992518	0.912356	56	ILMN_1790317	0.835108	219	3	77688
16	PKD1	5310	-0.00862	0.050382	0.864075	No	0.1167429	-0.09566	-0.05836	ILMN_1669645	0.194786	125	X7999614	0.48311	56	ILMN_2339028	0.390125	219	3	95930
16	TSC2	7249	-0.03255	0.05023	0.517013	No	0.1662055	-0.15756	-0.11476	ILMN_1714216	0.063958	125	X7992474	0.246155	56	ILMN_1714216	0.090236	219	3	143116
16	NTHL1	4913	-0.00934	0.050432	0.853058	No	0.0481751	-0.13922	-0.00951	ILMN_2227968	0.593679	125	X7998692	0.306151	56	ILMN_2227968	0.888708	219	3	183962
16	SLC9A3R2	9351	-0.16168	0.050093	0.001248	Yes	-0.0777532	-0.26071	-0.1836	ILMN_1765833	0.388754	125	X7992463	0.052297	56	ILMN_1765833	0.006437	219	3	192802
16	CCNF	899	0.008586	0.050471	0.864913	No	0.0336527	-0.0121	-0.00049	ILMN_1773119	0.709465	125	X7992594	0.929454	56	ILMN_1773119	0.994225	219	3	197565
16	NPW	283869	-0.18093	NA	0.182062	NA	NA	-0.18093	NA	NA	NA	NA	X7992460	0.182062	56	NA	NA	NA	1	211073
16	ZNF598	90850	-0.03246	0.050406	0.519546	No	0.0582955	-0.13564	-0.05813	ILMN_2075818	0.518431	125	X7998679	0.318898	56	ILMN_2075820	0.391989	219	3	222066
16	C16orf59	80178	0.00507	0.050459	0.919965	No	0.0554044	-0.04063	-0.01215	ILMN_1656452	0.539421	125	X7992614	0.766212	56	ILMN_1656452	0.858118	219	3	228285
16	SYNGR3	9143	0.019068	0.050437	0.705399	No	-0.0342181	-0.04549	0.065108	ILMN_1786379	0.704809	125	X7992447	0.739195	56	ILMN_1786379	0.337551	219	3	237553
16	NTN3	4917	-0.05003	0.050389	0.320766	No	-0.0906135	-0.17405	0.004149	ILMN_1656040	0.314906	125	X7992632	0.199522	56	ILMN_1656040	0.951324	219	3	239670
16	TBC1D24	57465	-0.00502	0.050437	0.920702	No	-0.0279624	-0.12055	0.036697	ILMN_2060212	0.756904	125	X7992639	0.376154	56	ILMN_2060212	0.589105	219	3	243317
16	GFER	2671	0.069058	0.050404	0.170665	Yes	0.0275133	0.071399	0.091896	ILMN_1768055	0.76069	125	X7992439	0.601029	56	ILMN_1768055	0.17541	219	3	244079
16	NOXO1	124056	0.066202	0.050339	0.188468	No	0.1174418	-0.13145	0.086204	ILMN_1708337	0.192109	125	X7998668	0.334194	56	ILMN_1708337	0.203809	219	3	250389
16	TBL3	10607	-0.05249	0.050366	0.297366	No	0.0608674	-0.08798	-0.10762	ILMN_2177413	0.500116	125	X7992414	0.519068	56	ILMN_2177413	0.112267	219	3	253078
16	RNF151	146310	0.021138	0.050406	0.674962	No	-0.0510096	-0.07217	0.0849	ILMN_1745330	0.572112	125	X7992409	0.59711	56	ILMN_1745330	0.210759	219	3	262853
16	RPS2	6187	-0.00327	0.050362	0.94819	No	0.0733914	-0.22648	0.009857	ILMN_2218277	0.415992	125	X8013348	0.093259	56	ILMN_2218277	0.884677	219	3	267002
16	SNORA64	26784	-0.00207	0.050432	0.967252	No	0.0843147	-0.03083	-0.04397	ILMN_1709604	0.349859	125	X7998666	0.821533	56	ILMN_1709604	0.517425	219	3	268722
16	SNORA10	574042	0.084257	0.050369	0.094367	Yes	0.1313007	0.02198	0.072831	ILMN_2189842	0.14441	125	X7998664	0.872254	56	ILMN_2189842	0.283248	219	3	269362
16	NDUFB10	4716	0.017451	0.050455	0.729432	No	0.0679655	-0.03605	0.002033	ILMN_1811754	0.451381	125	X7992402	0.791953	56	ILMN_1811754	0.976135	219	3	269853
16	RPL3L	6123	0.018402	0.050418	0.715123	No	0.1073162	-0.08209	-0.00728	ILMN_1775175	0.233567	125	X7998643	0.547543	56	ILMN_1775175	0.914665	219	3	277150
16	ATP6V0C	527	0.048562	0.050281	0.334142	No	-0.0746532	-0.08535	0.150516	ILMN_1773849	0.408	125	X7992646	0.531684	56	ILMN_1789005	0.025921	219	3	281897
16	AMDHD2	51005	-0.07739	0.050373	0.124444	No	-0.1162885	0.030625	-0.08192	ILMN_1695759	0.196541	125	X7992656	0.822712	56	ILMN_1695759	0.227252	219	3	288533
16	SEPX1	51734	-0.05131	0.050365	0.308341	No	0.0604184	-0.05394	-0.11359	ILMN_1719661	0.503289	125	X7998637	0.692988	56	ILMN_1719661	0.093598	219	3	288535
16	CEMP1	752014	0.053474	0.050326	0.287982	No	0.0895915	-0.18281	0.09218	ILMN_2280816	0.320418	125	X7998817	0.177486	56	ILMN_2280816	0.174075	219	3	298206
16	PDPK1	5170	-0.13377	0.050247	0.007762	Yes	-0.1409858	-0.10924	-0.13572	ILMN_1810554	0.116816	125	X7998820	0.422863	56	ILMN_1718306	0.04482	219	3	306140
16	HS3ST6	64711	-0.00689	0.050346	0.891158	No	-0.1047163	-0.14666	0.083372	ILMN_1710416	0.245154	125	X7998634	0.280773	56	ILMN_1710416	0.21912	219	3	313598
16	C16orf73	254528	-0.08179	0.050372	0.104456	Yes	-0.0264942	-0.12374	-0.10252	ILMN_1754241	0.769301	125	X7998615	0.363544	56	ILMN_1754241	0.130405	219	3	359650
16	LOC65227	652276	-0.19823	NA	0.143054	NA	NA	-0.19823	NA	NA	NA	NA	X7992678	0.143054	56	NA	NA	NA	1	371555
16	FAHD1	81889	0.023553	0.050389	0.640195	No	-0.0874758	0.011968	0.089218	ILMN_1701457	0.332024	125	X7992396	0.930242	56	ILMN_1694593	0.188383	219	3	391626
16	HAGH	3029	-0.01954	0.050374	0.698062	No	-0.0710038	-0.18802	0.051942	ILMN_1772731	0.431361	125	X7998604	0.165239	56	ILMN_2379520	0.444397	219	3	404634
16	FLJ42627	645644	-0.06884	0.054203	0.204106	NA	-0.1442418	NA	-0.02573	ILMN_1792882	0.108521	125	NA	NA	NA	ILMN_1792882	0.704923	219	2	407153
16	IGFALS	3483	-0.0143	0.050428	0.776802	No	0.0691983	-0.00828	-0.06304	ILMN_2188966	0.443196	125	X7998601	0.951687	56	ILMN_2188966	0.353144	219	3	436920
16	NUBP2	10101	0.022268	0.050373	0.658448	No	0.1503009	-0.05551	-0.03173	ILMN_1657237	0.094317	125	X7992388	0.684483	56	ILMN_1657237	0.640505	219	3	442637
16	SPSB3	90864	0.005186	0.050412	0.918067	No	1.47E-05	-0.16701	0.05121	ILMN_1682864	0.99987	125	X7998590	0.218588	56	ILMN_1682864	0.45085	219	3	449248
16	KCTD5	54442	-0.03708	0.050366	0.461636	No	-0.1319059	0.141737	-0.02759	ILMN_1652331	0.142552	125	X7992685	0.297405	56	ILMN_1652331	0.684701	219	3	450665
16	EME2	197342	0.040281	0.050421	0.424355	No	-0.0357036	0.058891	0.078576	ILMN_1771185	0.692626	125	X7992379	0.666371	56	ILMN_1771185	0.24688	219	3	455590
16	MRPS34	65993	-0.10856	0.050257	0.03077	Yes	-0.1991514	-0.16307	-0.04266	ILMN_2210482	0.025976	125	X7998585	0.229805	56	ILMN_2210482	0.530061	219	3	458689
16	NME3	4832	0.074044	0.050357	0.141457	Yes	0.145498	0.134491	0.018114	ILMN_1669456	0.105447	125	X7998577	0.323037	56	ILMN_1669456	0.78981	219	3	460119
16	MAPK8IP3	23162	-0.03443	0.050434	0.494774	No	0.0326894	-0.04676	-0.0693	ILMN_1811574	0.717424	125	X7992347	0.732202	56	ILMN_1811574	0.307303	219	3	461511
16	PRSS27	83886	-0.02424	0.050416	0.630609	No	0.0475933	-0.15776	-0.03152	ILMN_1775549	0.59815	125	X7998843	0.245545	56	ILMN_1775549	0.642726	219	3	480593

d) Test SNP=rs26840 and test allele=C

CHR	Gene	GeneID	Meta.bicor	Meta.se	Meta.p	same_direction_for_all_bicors	bicor.Study2	bicor.Study3	bicor.Study5	ID.Study2	p.Study2	n.Study2	ID.Study3	p.Study3	n.Study3	ID.Study5	p.Study5	n.Study5	num.data	dist_to_SNP (bp)
16	E4F1	1877	-0.04085	0.050704	0.420397	Yes	-0.0507194	-0.10026	-0.02047	ILMN_1720287	0.582222	120	X7992568	0.458069	57	ILMN_1720287	0.763286	219	3	0
16	DNASE1L2	1775	-0.00286	0.050716	0.95498	No	-0.0122572	-0.08721	0.023543	ILMN_1801366	0.894296	120	X7992584	0.518894	57	ILMN_1801366	0.729001	219	3	1110
16	ECI1	1632	0.017052	0.050727	0.736752	Yes	0.0353578	0.039874	0.001361	ILMN_2049303	0.70143	120	X7998762	0.768382	57	ILMN_2049303	0.984022	219	3	4515
16	RNPS1	10921	0.068954	0.050652	0.173414	Yes	0.1162502	0.11235	0.032134	ILMN_1691843	0.20608	120	X7998774	0.40537	57	ILMN_1691843	0.636254	219	3	17742
16	PGP	283871	0.196805	0.049982	8.23E-05	No	0.3467002	-0.10064	0.185588	ILMN_2057566	0.000105	120	X7998759	0.456362	57	ILMN_2057566	0.005875	219	3	20535
16	C16orf79	283870	0.023959	0.050667	0.6363	No	0.1078496	-0.10929	0.011911	ILMN_1759411	0.241	120	X7998749	0.41835	57	ILMN_1759411	0.860873	219	3	24288
16	MLST8	64223	-0.20952	0.050049	2.84E-05	Yes	-0.1013148	-0.39878	-0.21648	ILMN_1789240	0.270867	120	X7992552	0.002122	57	ILMN_1789240	0.001266	219	3	25939
16	CASKIN1	57524	-0.01553	0.05071	0.75936	No	-0.0737807	-0.02729	0.019121	ILMN_1679039	0.423213	120	X7998724	0.840284	57	ILMN_1679039	0.77843	219	3	38892
16	ABCA3	21	0.01504	0.050706	0.766764	No	0.0418126	-0.09376	0.028004	ILMN_1758523	0.650233	120	X7998784	0.487843	57	ILMN_1758523	0.680248	219	3	40521
16	TRAF7	84231	-0.00677	0.050669	0.893772	No	-0.0684987	-0.1194	0.055281	ILMN_2405991	0.457248	120	X7992529	0.376341	57	ILMN_2405991	0.415627	219	3	57227
16	SNORD60	26788	0.006803	NA	0.959947	NA	NA	0.006803	NA	NA	NA	NA	X7998722	0.959947	57	NA	NA	NA	1	80251
16	RAB26	25837	0.039454	0.050663	0.436127	No	0.1304716	0.048641	-0.01268	ILMN_1790317	0.155502	120	X7992518	0.719365	57	ILMN_1790317	0.85198	219	3	81216
16	PKD1	5310	0.004853	0.050663	0.923694	No	0.0946197	0.073989	-0.06141	ILMN_1669645	0.303966	120	X7998700	0.584394	57	ILMN_2339028	0.365739	219	3	99458
16	TSC2	7249	-0.02216	0.050522	0.660891	No	0.1699043	-0.11179	-0.10478	ILMN_1714216	0.063562	120	X7992474	0.407742	57	ILMN_1714216	0.122116	219	3	146644
16	NTHL1	4913	0.011852	0.050649	0.81498	No	0.1105115	-0.14607	-0.00184	ILMN_2227968	0.229516	120	X7998692	0.278288	57	ILMN_2227968	0.978432	219	3	187490
16	CCNF	899	0.008702	0.050726	0.863793	No	0.0459573	-0.00347	-0.00847	ILMN_1773119	0.61818	120	X7992594	0.979571	57	ILMN_1773119	0.900763	219	3	194037
16	SLC9A3R2	9351	-0.14908	0.050392	0.003092	Yes	-0.0578389	-0.25984	-0.16952	ILMN_1765833	0.53034	120	X7992463	0.050946	57	ILMN_1765833	0.011987	219	3	196330
16	NPW	283869	-0.18064	NA	0.178722	NA	NA	-0.18064	NA	NA	NA	NA	X7992460	0.178722	57	NA	NA	NA	1	214601
16	C16orf59	80178	0.012037	0.05071	0.812367	No	0.0754726	-0.01252	-0.0163	ILMN_1656452	0.412629	120	X7992614	0.926346	57	ILMN_1656452	0.810413	219	3	224757
16	ZNF598	90850	-0.04692	0.050649	0.354284	No	0.0469721	-0.15005	-0.07161	ILMN_2075818	0.610438	120	X7998679	0.265244	57	ILMN_2075820	0.291448	219	3	225594
16	NTN3	4917	-0.05953	0.050605	0.239412	No	-0.1373609	-0.17487	0.012591	ILMN_1656040	0.134637	120	X7992632	0.193251	57	ILMN_1656040	0.853022	219	3	236142
16	TBC1D24	57465	-0.01076	0.050703	0.831965	No	-0.0292678	-0.11374	0.025374	ILMN_2060212	0.750997	120	X7992639	0.399541	57	ILMN_2060212	0.708837	219	3	239789
16	SYNGR3	9143	0.007222	0.050702	0.886735	No	-0.0470455	-0.05147	0.051444	ILMN_1786379	0.609879	120	X7992447	0.703757	57	ILMN_1786379	0.448783	219	3	241081
16	GFER	2671	0.07257	0.050663	0.152025	Yes	0.0455263	0.068265	0.088288	ILMN_1768055	0.621481	120	X7992439	0.613865	57	ILMN_1768055	0.193052	219	3	247607
16	NOXO1	124056	0.062638	0.050602	0.215769	No	0.0943884	-0.13625	0.095716	ILMN_1708337	0.305155	120	X7998668	0.312202	57	ILMN_1708337	0.158065	219	3	253917
16	TBL3	10607	-0.04055	0.050631	0.423166	No	0.0819476	-0.08907	-0.09475	ILMN_2177413	0.373577	120	X7992414	0.509968	57	ILMN_2177413	0.162313	219	3	256606
16	RNF151	146310	0.02287	0.050655	0.651635	No	-0.0629462	-0.06045	0.090275	ILMN_1745330	0.494606	120	X7992409	0.65511	57	ILMN_1745330	0.183182	219	3	266381
16	RPS2	6187	-0.00139	0.050622	0.978092	No	0.0507875	-0.22904	0.02911	ILMN_2218277	0.581715	120	X8013348	0.086577	57	ILMN_2218277	0.668347	219	3	270530
16	SNORA64	26784	-0.00821	0.050708	0.871426	No	0.059933	-0.03053	-0.03961	ILMN_1709604	0.515534	120	X7998666	0.821657	57	ILMN_1709604	0.55987	219	3	272250
16	SNORA10	574042	0.064577	0.050676	0.202559	Yes	0.0839899	0.020696	0.065052	ILMN_2189842	0.361745	120	X7998664	0.878552	57	ILMN_2189842	0.337972	219	3	272890
16	NDUFB10	4716	0.017616	0.050695	0.728233	No	0.0793549	-0.08804	0.010705	ILMN_1811754	0.388935	120	X7992402	0.514876	57	ILMN_1811754	0.874835	219	3	273381
16	ATP6V0C	527	0.044098	0.050482	0.38237	No	-0.1201836	-0.07371	0.162069	ILMN_1773849	0.19104	120	X7992646	0.585807	57	ILMN_1789005	0.01637	219	3	278369
16	RPL3L	6123	0.007715	0.050699	0.879045	No	0.0661212	-0.10053	0.003336	ILMN_1775175	0.47305	120	X7998643	0.456838	57	ILMN_1775175	0.960846	219	3	280678
16	AMDHD2	51005	-0.07274	0.050649	0.150937	No	-0.0944653	0.021364	-0.08455	ILMN_1695759	0.304759	120	X7992656	0.874663	57	ILMN_1695759	0.212632	219	3	285005
16	SEPX1	51734	-0.04184	0.050604	0.408372	No	0.0974541	-0.08592	-0.10629	ILMN_1719661	0.289643	120	X7998637	0.525096	57	ILMN_1719661	0.116787	219	3	292063
16	CEMP1	752014	0.062886	0.050563	0.213601	No	0.1032664	-0.17692	0.101984	ILMN_2280816	0.261697	120	X7998817	0.187991	57	ILMN_2280816	0.132447	219	3	294678
16	PDPK1	5170	-0.13943	0.050483	0.005748	Yes	-0.1437232	-0.10081	-0.14676	ILMN_1653793	0.117332	120	X7998820	0.455596	57	ILMN_1718306	0.029916	219	3	302612
16	HS3ST6	64711	-0.01373	0.050581	0.786054	No	-0.1246948	-0.15682	0.083105	ILMN_1710416	0.174791	120	X7998634	0.244028	57	ILMN_1710416	0.220603	219	3	317126
16	C16orf73	254528	-0.04377	0.050609	0.38715	No	0.0893804	-0.13913	-0.09187	ILMN_1754241	0.331643	120	X7998615	0.302003	57	ILMN_1754241	0.175517	219	3	363178
16	LOC65227	652276	-0.21763	NA	0.103899	NA	NA	-0.21763	NA	NA	NA	NA	X7992678	0.103899	57	NA	NA	NA	1	368027
16	FAHD1	81889	0.014781	0.05062	0.770282	No	-0.1266433	0.060245	0.08048	ILMN_1701457	0.168097	120	X7992396	0.656201	57	ILMN_1694593	0.235583	219	3	395154
16	FLJ42627	645644	-0.08207	0.054558	0.132525	NA	-0.1754884	NA	-0.03059	ILMN_1792882	0.055217	120	NA	NA	NA	ILMN_1792882	0.652539	219	2	403625
16	HAGH	3029	-0.00888	0.050644	0.86077	No	-0.0664383	-0.16084	0.061052	ILMN_2379520	0.470925	120	X7998604	0.231992	57	ILMN_2379520	0.368568	219	3	408162
16	IGFALS	3483	-0.02208	0.050701	0.663256	No	0.043282	-0.00958	-0.06066	ILMN_2188966	0.638793	120	X7998601	0.943627	57	ILMN_2188966	0.371684	219	3	440448
16	NUBP2	10101	0.015598	0.050646	0.758102	No	0.1390216	-0.05477	-0.03427	ILMN_1657237	0.129942	120	X7992388	0.685724	57	ILMN_1657237	0.613949	219	3	446165
16	KCTD5	54442	-0.04853	0.050551	0.337088	No	-0.169995	0.180969	-0.04038	ILMN_1652331	0.063419	120	X7992685	0.177925	57	ILMN_1652331	0.552272	219	3	447137
16	SPSB3	90864	0.00198	0.050657	0.968818	No	-0.028649	-0.16264	0.060503	ILMN_1682864	0.756095	120	X7998590	0.226762	57	ILMN_1682864	0.372901	219	3	452776
16	EME2	197342	0.052078	0.050676	0.30411	No	-0.012396	0.060854	0.084741	ILMN_1771185	0.893106	120	X7992379	0.652947	57	ILMN_1771185	0.211618	219	3	459118
16	MRPS34	65993	-0.11242	0.050503	0.026018	Yes	-0.2012827	-0.17888	-0.04625	ILMN_2210482	0.027491	120	X7998585	0.183077	57	ILMN_2210482	0.495971	219	3	462217
16	NME3	4832	0.080415	0.0506	0.112012	Yes	0.1547547	0.143731	0.023486	ILMN_1669456	0.09147	120	X7998577	0.286128	57	ILMN_1669456	0.729628	219	3	463647
16	MAPK8IP3	23162	-0.0306	0.05069	0.546088	No	0.0382228	-0.00544	-0.07419	ILMN_1811574	0.678523	120	X7992347	0.967952	57	ILMN_1811574	0.274343	219	3	465039
16	PRSS27	83886	-0.03224	0.050662	0.524474	No	0.0323959	-0.18593	-0.02797	ILMN_1775549	0.725396	120	X7998843	0.166121	57	ILMN_1775549	0.680598	219	3	477065

e) Test SNP= rs27648 and test allele=G

CHR	Gene	GeneID	Meta.bicor	Meta.se	Meta.p	same_direction_for_all_bicors	bicor.Study2	bicor.Study3	bicor.Study5	ID.Study2	p.Study2	n.Study2	ID.Study3	p.Study3	n.Study3	ID.Study5	p.Study5	n.Study5	num.data	dist_to_SNP (bp)
16	ECI1	1632	-0.00087	0.050992	0.986347	No	0.0283894	0.001397	-0.01754	ILMN_2049303	0.756253	122	X7998762	0.992236	51	ILMN_2049303	0.796365	219	3	0
16	DNASE1L2	1775	0.011298	0.05094	0.824472	No	0.0502486	-0.15996	0.028632	ILMN_1801366	0.582561	122	X7992584	0.26218	51	ILMN_1801366	0.673483	219	3	2638
16	E4F1	1877	-0.03618	0.050963	0.477712	Yes	-0.0391884	-0.12824	-0.01373	ILMN_1720287	0.668245	122	X7992568	0.369798	51	ILMN_1720287	0.839899	219	3	5607
16	RNPS1	10921	0.080322	0.050889	0.114481	Yes	0.1420294	0.095041	0.042635	ILMN_1691843	0.118633	122	X7998774	0.507072	51	ILMN_1691843	0.53026	219	3	11749
16	PGP	283871	0.197404	0.050267	8.60E-05	No	0.3169386	-0.12194	0.199897	ILMN_2057566	0.000375	122	X7998759	0.393982	51	ILMN_2057566	0.002965	219	3	26528
16	C16orf79	283870	0.049277	0.050883	0.332829	No	0.157362	-0.08702	0.019522	ILMN_1759411	0.083447	122	X7998749	0.543722	51	ILMN_1759411	0.773898	219	3	30281
16	MLST8	64223	-0.20765	0.05041	3.80E-05	Yes	-0.1504008	-0.30256	-0.21709	ILMN_1789240	0.098219	122	X7992552	0.03093	51	ILMN_1789240	0.001226	219	3	31932
16	ABCA3	21	0.010914	0.050985	0.83049	No	0.0259146	-0.06731	0.020194	ILMN_1758523	0.776918	122	X7998784	0.638855	51	ILMN_1758523	0.766336	219	3	34528
16	CASKIN1	57524	-0.02438	0.050972	0.632472	No	-0.0556779	-0.08998	0.007681	ILMN_1679039	0.542442	122	X7998724	0.53007	51	ILMN_1679039	0.91001	219	3	44885

Supplementary Table 5: Functional enrichment study of SNP sets that are associated with epigenetic age acceleration

Here we used the MAGENTA software to evaluate what is known about the set of SNPs that is associated with epigenetic age acceleration in the cerebellum. The analysis is only based on the meta-analysis GWAS results of epigenetic age acceleration in the cerebellum, i.e. it does not make use of transcriptional data. While the gene sets are nominally significant, they are not significant after adjusting for multiple comparisons using the false discovery rate approach (FDR > 0.1). The rows correspond to the most significant results (at a FDR < 0.25) from the MAGENTA analysis. The flagged genes (which are listed in ascending order) have an observed *P* value that is beyond the 95th percentile of the *P* values for the entire set of human autosomal genes.

**Database**	**Pathway/Gene set (size)**	**Nominal $P_{MAGENTA}$**	**FDR***	**Flagged genes**
PANTHER molecular function	Complement component (51)	0.0048	0.16	*CFHR4 CFD CFHR3 CFH CFHR1 CFHR2 CFB*
	Glutamate receptor (20)	0.014	0.18	*GRIA4 GRIA1 GRIK1 GRIN3A*
	Protease inhibitor (21)	0.021	0.23	*BIRC2 BIRC3 BIRC7*
	DNA helicase (76)	0.013	0.25	*ERCC6 MCM7 CHD9 MCM9 SMARCA5 SKIV2L BLM DDX11*
PANTHER biological process	Other amino acid metabolism (30)	0.0083	0.22	*AANAT HDC PDXDC1 GAD2 MAT1A*

*output from the MAGENTA algorithm.

Supplementary Table 6: Genes that relate to cerebellar age acceleration and to at least one age-related disease according to the GWAS overlap analysis

The Excel file reports gene symbols and gene identifiers for genes that relate both to epigenetic age acceleration (in the cerebellum) and an age related disease (first column), according to the "overlap analysis" of GWA studies. The first column reports the GWA study for the age related disease under consideration. To define lists of genes that relate to a given trait (disease status or epigenetic age acceleration) we used a MAGENTA gene score cut-off based on the 95th percentile of the autosomal genes. In other words, we formed a list of the top 5% most significant genes for each trait. Next, we assessed which of these genes is also located in the top 5% list of epigenetic age acceleration in the cerebellum.

AMD=age-realated macular degeneration; ALZ=Alzheimer's disease

**GWAS Study**	**CHR**	**Gene Symbol**	**Gene ID**
AMD	1	CGN	57530
AMD	1	TUFT1	7286
AMD	1	CFH	3075
AMD	1	CFHR3	10878
AMD	1	CFHR1	3078
AMD	1	CFHR4	10877
AMD	1	CFHR2	3080
AMD	1	CFHR5	81494
AMD	1	LHX9	56956
AMD	2	POMC	5443
AMD	2	FAM82A1	151393
AMD	2	NDUFA10	4705
AMD	3	CLASP2	23122
AMD	4	REST	5978
AMD	4	C4orf14	84273
AMD	4	POLR2B	5431
AMD	4	IGFBP7	3490
AMD	5	VCAN	1462
AMD	6	SLC44A4	80736
AMD	6	EHMT2	10919
AMD	6	ZBTB12	221527
AMD	6	C2	717
AMD	6	CFB	629
AMD	6	RDBP	7936
AMD	6	SKIV2L	6499
AMD	6	DOM3Z	1797
AMD	6	STK19	8859
AMD	7	AZGP1	563
AMD	7	ZKSCAN1	7586
AMD	7	ZSCAN21	7589
AMD	7	ZNF3	7551
AMD	7	COPS6	10980
AMD	7	MCM7	4176
AMD	7	AP4M1	9179
AMD	7	TAF6	6878
AMD	7	CNPY4	245812
AMD	7	MBLAC1	255374
AMD	7	C7orf59	389541
AMD	7	C7orf43	55262
AMD	7	GAL3ST4	79690
AMD	7	GPC2	221914

AMD	7	STAG3	10734
AMD	7	GATS	352954
AMD	7	PVRIG	79037
AMD	7	SPDYE3	441272
AMD	7	PMS2P1	5379
AMD	7	PILRB	29990
AMD	7	PILRA	29992
AMD	7	ZCWPW1	55063
AMD	7	MEPCE	56257
AMD	7	C7orf47	221908
AMD	7	C7orf61	402573
AMD	7	TSC22D4	81628
AMD	7	C7orf51	222950
AMD	7	AGFG2	3268
AMD	9	DNAJB5	25822
AMD	9	GADD45G	10912
AMD	9	SUSD1	64420
AMD	10	MAT1A	4143
AMD	10	INPP5A	3632
AMD	12	WNK1	378465
AMD	12	BTBD11	121551
AMD	14	DCAF4	26094
AMD	14	ZFYVE1	53349
AMD	15	MEIS2	4212
AMD	15	LIPC	3990
AMD	16	CLCN7	1186
AMD	16	PTX4	390667
AMD	16	TELO2	9894
AMD	16	IFT140	9742
AMD	16	TMEM204	79652
AMD	16	CHD9	80205
AMD	16	JPH3	57338
AMD	17	CBX1	10951
AMD	17	SNX11	29916
AMD	18	RAB27B	5874
AMD	18	TNFRSF11A	8792
AMD	21	C21orf70	85395
AMD	22	RTN4R	65078
ALZ	7	AZGP1	563
ALZ	7	ZKSCAN1	7586
ALZ	7	ZSCAN21	7589

ALZ	7	ZNF3	7551
ALZ	7	COPS6	10980
ALZ	7	MCM7	4176
ALZ	7	AP4M1	9179
ALZ	7	TAF6	6878
ALZ	7	CNPY4	245812
ALZ	7	MBLAC1	255374
ALZ	7	C7orf59	389541
ALZ	7	C7orf43	55262
ALZ	7	STAG3	10734
ALZ	7	GATS	352954
ALZ	7	PMS2P1	5379
ALZ	7	PILRB	29990
ALZ	7	PILRA	29992
ALZ	7	ZCWPW1	55063
ALZ	7	MEPCE	56257
ALZ	7	C7orf47	221908
ALZ	7	C7orf61	402573
ALZ	7	TSC22D4	81628
ALZ	7	C7orf51	222950
ALZ	7	AGFG2	3268
ALZ	11	DDB2	1643
ALZ	11	MADD	8567
ALZ	11	MYBPC3	4607
ALZ	11	CCDC83	220047
ALZ	11	PICALM	8301
HRS ALL Cogitive slope	1	ZSCAN20	7579
HRS ALL Cogitive slope	2	RBM45	129831
HRS ALL Cogitive slope	4	D4S234E	27065
HRS ALL Cogitive slope	4	SCFD2	152579
HRS ALL Cogitive slope	4	IGFBP7	3490
HRS ALL Cogitive slope	7	FAM20C	56975
HRS ALL Cogitive slope	7	PMS2P11	441263
HRS ALL Cogitive slope	7	LOC100132832	1E+08
HRS ALL Cogitive slope	7	C7orf68	29923
HRS ALL Cogitive slope	10	IDI2	91734
HRS ALL Cogitive slope	10	IDI2-AS1	55853
HRS ALL Cogitive slope	10	IDI1	3422
HRS ALL Cogitive slope	10	WDR37	22884
HRS ALL Cogitive slope	10	DCLRE1C	64421
HRS ALL Cogitive slope	10	MEIG1	644890

HRS ALL Cogitive slope	10	EIF5AL1	143244
HRS ALL Cogitive slope	10	SFTPA2	729238
HRS ALL Cogitive slope	11	HYLS1	219844
HRS ALL Cogitive slope	11	PUS3	83480
HRS ALL Cogitive slope	11	DDX25	29118
HRS ALL Cogitive slope	11	CDON	50937
HRS ALL Cogitive slope	12	DDX11	1663
HRS ALL Cogitive slope	12	DBX2	440097
HRS ALL Cogitive slope	12	LOC100128554	1E+08
HRS ALL Cogitive slope	14	TMEM90A	646658
HRS ALL Cogitive slope	15	SNURF	8926
HRS ALL Cogitive slope	15	GABPB1	2553
HRS ALL Cogitive slope	15	BLM	641
HRS ALL Cogitive slope	16	SETD6	79918
HRS ALL Cogitive slope	16	CNOT1	23019
HRS ALL Cogitive slope	16	COX4NB	10328
HRS ALL Cogitive slope	17	RPH3AL	9501
HRS ALL Cogitive slope	17	MAPK7	5598
HRS ALL Cogitive slope	17	MFAP4	4239
HRS ALL Cogitive slope	17	RNF112	7732
HRS ALL Cogitive slope	17	CBX1	10951
HRS ALL Cogitive slope	17	SNX11	29916
HRS ALL Cogitive slope	17	AANAT	15
HRS ALL Cogitive slope	17	RHBDF2	79651
HRS ALL Cogitive slope	19	PTBP1	5725
HRS ALL Cogitive slope	19	LPPR3	79948
HRS ALL Cogitive slope	19	AZU1	566
HRS ALL Cogitive slope	19	PRTN3	5657
HRS ALL Cogitive slope	19	ELANE	1991
HRS ALL Cogitive slope	19	CFD	1675
HRS ALL Cogitive slope	19	MED16	10025
HRS ALL Cogitive slope	19	CYP2B6	1555
HRS ALL Cogitive slope	19	ZNF473	25888
HRS ALL Cogitive slope	19	FLJ26850	400710
HRS ALL Cogitive slope	19	KLK10	5655
HRS ALL Cogitive slope	19	KLK11	11012
HRS ALL Cogitive slope	19	KLK12	43849
HRS ALL Cogitive slope	19	KLK13	26085
HRS ALL Cogitive slope	20	RNF24	11237
HRS ALL Cogitive slope	21	C21orf70	85395
HRS EUR Cogitive slope	1	ZSCAN20	7579

HRS EUR Cogitive slope	1	CLCA4	22802
HRS EUR Cogitive slope	1	HHIPL2	79802
HRS EUR Cogitive slope	1	TAF1A	9015
HRS EUR Cogitive slope	2	ATP6V1C2	245973
HRS EUR Cogitive slope	2	PDIA6	10130
HRS EUR Cogitive slope	2	DPY30	84661
HRS EUR Cogitive slope	2	ATOH8	84913
HRS EUR Cogitive slope	3	ISY1	57461
HRS EUR Cogitive slope	3	SPATA16	83893
HRS EUR Cogitive slope	4	REST	5978
HRS EUR Cogitive slope	4	C4orf14	84273
HRS EUR Cogitive slope	6	MOCS1	4337
HRS EUR Cogitive slope	6	BEND6	221336
HRS EUR Cogitive slope	7	FAM20C	56975
HRS EUR Cogitive slope	7	PMS2P11	441263
HRS EUR Cogitive slope	7	LOC100132832	1E+08
HRS EUR Cogitive slope	7	COPS6	10980
HRS EUR Cogitive slope	7	MCM7	4176
HRS EUR Cogitive slope	7	AP4M1	9179
HRS EUR Cogitive slope	7	TAF6	6878
HRS EUR Cogitive slope	7	CNPY4	245812
HRS EUR Cogitive slope	7	MBLAC1	255374
HRS EUR Cogitive slope	7	C7orf59	389541
HRS EUR Cogitive slope	7	C7orf43	55262
HRS EUR Cogitive slope	7	GAL3ST4	79690
HRS EUR Cogitive slope	7	GPC2	221914
HRS EUR Cogitive slope	7	STAG3	10734
HRS EUR Cogitive slope	8	GINS4	84296
HRS EUR Cogitive slope	9	ZBTB43	23099
HRS EUR Cogitive slope	9	AK8	158067
HRS EUR Cogitive slope	10	IDI2	91734
HRS EUR Cogitive slope	10	IDI2-AS1	55853
HRS EUR Cogitive slope	10	IDI1	3422
HRS EUR Cogitive slope	10	WDR37	22884
HRS EUR Cogitive slope	10	SFTPA2	729238
HRS EUR Cogitive slope	11	CCDC82	79780
HRS EUR Cogitive slope	11	HYLS1	219844
HRS EUR Cogitive slope	11	PUS3	83480
HRS EUR Cogitive slope	11	DDX25	29118
HRS EUR Cogitive slope	11	CDON	50937
HRS EUR Cogitive slope	12	BTBD11	121551

HRS EUR Cogitive slope	12	TESC	54997
HRS EUR Cogitive slope	13	LMO7	4008
HRS EUR Cogitive slope	14	TMEM90A	646658
HRS EUR Cogitive slope	15	SNURF	8926
HRS EUR Cogitive slope	15	GABPB1	2553
HRS EUR Cogitive slope	16	C16orf74	404550
HRS EUR Cogitive slope	16	COX4NB	10328
HRS EUR Cogitive slope	17	RPH3AL	9501
HRS EUR Cogitive slope	17	FMNL1	752
HRS EUR Cogitive slope	17	CBX1	10951
HRS EUR Cogitive slope	17	SNX11	29916
HRS EUR Cogitive slope	19	KRI1	65095
HRS EUR Cogitive slope	19	CDKN2D	1032
HRS EUR Cogitive slope	19	CYP2B6	1555
HRS EUR Cogitive slope	20	RNF24	11237
HRS EUR Cogitive slope	20	COMMD7	149951
HRS EUR Cogitive slope	21	C21orf70	85395
HRS EUR Cogitive slope	22	BIK	638
HRS EUR Cogitive slope	22	MCAT	27349
HRS AFR Cogitive slope	1	KISS1	3814
HRS AFR Cogitive slope	1	GOLT1A	127845
HRS AFR Cogitive slope	1	MOSC1	64757
HRS AFR Cogitive slope	2	LTBP1	4052
HRS AFR Cogitive slope	2	LY75	4065
HRS AFR Cogitive slope	2	PLA2R1	22925
HRS AFR Cogitive slope	2	RBM45	129831
HRS AFR Cogitive slope	3	SPATA16	83893
HRS AFR Cogitive slope	3	TNK2	10188
HRS AFR Cogitive slope	4	D4S234E	27065
HRS AFR Cogitive slope	4	SCFD2	152579
HRS AFR Cogitive slope	5	UNC5A	90249
HRS AFR Cogitive slope	6	SLC44A4	80736
HRS AFR Cogitive slope	6	EHMT2	10919
HRS AFR Cogitive slope	6	ZBTB12	221527
HRS AFR Cogitive slope	6	C2	717
HRS AFR Cogitive slope	6	CFB	629
HRS AFR Cogitive slope	6	RDBP	7936
HRS AFR Cogitive slope	6	SKIV2L	6499
HRS AFR Cogitive slope	6	DOM3Z	1797
HRS AFR Cogitive slope	6	STK19	8859
HRS AFR Cogitive slope	7	FAM20C	56975

HRS AFR Cogitive slope	9	DNAI1	27019
HRS AFR Cogitive slope	10	SFTA1P	207107
HRS AFR Cogitive slope	10	C10orf122	387718
HRS AFR Cogitive slope	11	CCDC82	79780
HRS AFR Cogitive slope	11	ACAD8	27034
HRS AFR Cogitive slope	12	RPL13AP20	387841
HRS AFR Cogitive slope	12	GPRC5A	9052
HRS AFR Cogitive slope	12	CAPZA3	93661
HRS AFR Cogitive slope	12	CMKLR1	1240
HRS AFR Cogitive slope	12	GPR109A	338442
HRS AFR Cogitive slope	13	UCHL3	7347
HRS AFR Cogitive slope	13	LMO7	4008
HRS AFR Cogitive slope	14	STON2	85439
HRS AFR Cogitive slope	15	MEIS2	4212
HRS AFR Cogitive slope	16	ACSM2A	123876
HRS AFR Cogitive slope	16	FBXO31	79791
HRS AFR Cogitive slope	17	C17orf91	84981
HRS AFR Cogitive slope	17	WDR81	124997
HRS AFR Cogitive slope	17	PRPSAP2	5636
HRS AFR Cogitive slope	17	SLC5A10	125206
HRS AFR Cogitive slope	17	FAM83G	644815
HRS AFR Cogitive slope	17	GRAP	10750
HRS AFR Cogitive slope	17	SRCIN1	80725
HRS AFR Cogitive slope	17	SPOP	8405
HRS AFR Cogitive slope	17	SLC35B1	10237
HRS AFR Cogitive slope	17	FAM117A	81558
HRS AFR Cogitive slope	17	PITPNC1	26207
HRS AFR Cogitive slope	17	ABCA9	10350
HRS AFR Cogitive slope	19	FZR1	51343
HRS AFR Cogitive slope	19	LILRB4	11006
HRS AFR Cogitive slope	19	LILRP2	79166
HRS AFR Cogitive slope	20	ARFGAP1	55738
HRS AFR Cogitive slope	20	COL20A1	57642
HRS AFR Cogitive slope	21	RRP1B	23076
HRS AFR Cogitive slope	22	SHANK3	85358
HRS AMR Cogitive slope	1	CLCA4	22802
HRS AMR Cogitive slope	1	SMG5	23381
HRS AMR Cogitive slope	1	TMEM79	84283
HRS AMR Cogitive slope	1	C1orf85	112770
HRS AMR Cogitive slope	1	VHLL	391104
HRS AMR Cogitive slope	1	CCT3	7203

HRS AMR Cogitive slope	1	C1orf182	128229
HRS AMR Cogitive slope	1	EPRS	2058
HRS AMR Cogitive slope	1	HHIPL2	79802
HRS AMR Cogitive slope	1	TAF1A	9015
HRS AMR Cogitive slope	2	ARHGEF33	1E+08
HRS AMR Cogitive slope	2	HAAO	23498
HRS AMR Cogitive slope	2	VRK2	7444
HRS AMR Cogitive slope	2	FANCL	55120
HRS AMR Cogitive slope	2	SESTD1	91404
HRS AMR Cogitive slope	2	C2orf85	285093
HRS AMR Cogitive slope	3	SDHAP1	255812
HRS AMR Cogitive slope	4	SCFD2	152579
HRS AMR Cogitive slope	7	LOC100132832	1E+08
HRS AMR Cogitive slope	7	CACNA2D1	781
HRS AMR Cogitive slope	7	EMID2	136227
HRS AMR Cogitive slope	7	MET	4233
HRS AMR Cogitive slope	7	GPR37	2861
HRS AMR Cogitive slope	8	CLN8	2055
HRS AMR Cogitive slope	8	ARHGEF10	9639
HRS AMR Cogitive slope	11	UVRAG	7405
HRS AMR Cogitive slope	11	FAM55B	120406
HRS AMR Cogitive slope	11	GLB1L2	89944
HRS AMR Cogitive slope	12	PPFIBP1	8496
HRS AMR Cogitive slope	12	PTPRQ	374462
HRS AMR Cogitive slope	12	MYF6	4618
HRS AMR Cogitive slope	13	RCBTB2	1102
HRS AMR Cogitive slope	14	OTX2	5015
HRS AMR Cogitive slope	14	VRTN	55237
HRS AMR Cogitive slope	14	TMEM90A	646658
HRS AMR Cogitive slope	14	STON2	85439
HRS AMR Cogitive slope	15	SLC27A2	11001
HRS AMR Cogitive slope	15	HDC	3067
HRS AMR Cogitive slope	15	GABPB1	2553
HRS AMR Cogitive slope	15	MAPK6	5597
HRS AMR Cogitive slope	18	POTEC	388468
HRS AMR Cogitive slope	19	AZU1	566
HRS AMR Cogitive slope	19	PRTN3	5657
HRS AMR Cogitive slope	19	ELANE	1991
HRS AMR Cogitive slope	19	CFD	1675
HRS AMR Cogitive slope	19	FZR1	51343
HRS AMR Cogitive slope	20	CPXM1	56265

HRS AMR Cogitive slope	20	GNRH2	2797
HRS AMR Cogitive slope	20	MRPS26	64949
HRS AMR Cogitive slope	22	FLJ41941	1E+08
HRS ALL Dementia Wave 8	1	PEX14	5195
HRS ALL Dementia Wave 8	2	SRSF7	6432
HRS ALL Dementia Wave 8	2	GEMIN6	79833
HRS ALL Dementia Wave 8	2	DHX57	90957
HRS ALL Dementia Wave 8	2	TANK	10010
HRS ALL Dementia Wave 8	2	LASS6	253782
HRS ALL Dementia Wave 8	3	TNK2	10188
HRS ALL Dementia Wave 8	4	D4S234E	27065
HRS ALL Dementia Wave 8	5	TRIP13	9319
HRS ALL Dementia Wave 8	7	SUN1	23353
HRS ALL Dementia Wave 8	7	FSCN1	6624
HRS ALL Dementia Wave 8	7	COBL	23242
HRS ALL Dementia Wave 8	7	MUC17	140453
HRS ALL Dementia Wave 8	7	CTTNBP2	83992
HRS ALL Dementia Wave 8	8	NIPAL2	79815
HRS ALL Dementia Wave 8	8	SQLE	6713
HRS ALL Dementia Wave 8	10	GAD2	2572
HRS ALL Dementia Wave 8	11	YAP1	10413
HRS ALL Dementia Wave 8	11	CDON	50937
HRS ALL Dementia Wave 8	11	GLB1L2	89944
HRS ALL Dementia Wave 8	12	MRPS35	60488
HRS ALL Dementia Wave 8	12	LALBA	3906
HRS ALL Dementia Wave 8	12	COQ10A	93058
HRS ALL Dementia Wave 8	12	CS	1431
HRS ALL Dementia Wave 8	12	CSRP2	1466
HRS ALL Dementia Wave 8	12	WSCD2	9671
HRS ALL Dementia Wave 8	12	CMKLR1	1240
HRS ALL Dementia Wave 8	12	LOC100128554	1E+08
HRS ALL Dementia Wave 8	13	TRPC4	7223
HRS ALL Dementia Wave 8	14	ACTN1	87
HRS ALL Dementia Wave 8	16	LOC652276	652276
HRS ALL Dementia Wave 8	16	FLJ42627	645644
HRS ALL Dementia Wave 8	16	CDH11	1009
HRS ALL Dementia Wave 8	17	PRPF8	10594
HRS ALL Dementia Wave 8	17	C17orf91	84981
HRS ALL Dementia Wave 8	17	WDR81	124997
HRS ALL Dementia Wave 8	17	SERPINF2	5345
HRS ALL Dementia Wave 8	17	FLJ36000	284124

HRS ALL Dementia Wave 8	18	POTEC	388468
HRS ALL Dementia Wave 8	18	LOXHD1	125336
HRS ALL Dementia Wave 8	18	MBP	4155
HRS ALL Dementia Wave 8	19	C19orf77	284422
HRS ALL Dementia Wave 8	20	DEFB119	245932
HRS ALL Dementia Wave 8	20	DEFB121	245934
HRS ALL Dementia Wave 8	20	DEFB123	245936
HRS ALL Dementia Wave 8	20	DEFB124	245937
HRS ALL Dementia Wave 8	20	REM1	28954
HRS ALL Dementia Wave 8	20	SGK2	10110
HRS ALL Dementia Wave 8	20	IFT52	51098
HRS ALL Dementia Wave 8	21	CCT8	10694
HRS ALL Dementia Wave 8	21	PTTG1IP	754
HRS ALL Dementia Wave 8	21	COL18A1	80781
HRS EUR Dementia Wave 8	1	YTHDF2	51441
HRS EUR Dementia Wave 8	1	CLCA4	22802
HRS EUR Dementia Wave 8	2	SRSF7	6432
HRS EUR Dementia Wave 8	2	GEMIN6	79833
HRS EUR Dementia Wave 8	2	DHX57	90957
HRS EUR Dementia Wave 8	2	MORN2	729967
HRS EUR Dementia Wave 8	2	MEIS1	4211
HRS EUR Dementia Wave 8	2	SDPR	8436
HRS EUR Dementia Wave 8	3	BDH1	622
HRS EUR Dementia Wave 8	4	BANK1	55024
HRS EUR Dementia Wave 8	6	ZNF451	26036
HRS EUR Dementia Wave 8	6	BAG2	9532
HRS EUR Dementia Wave 8	6	RAB23	51715
HRS EUR Dementia Wave 8	6	SGK1	6446
HRS EUR Dementia Wave 8	7	SUN1	23353
HRS EUR Dementia Wave 8	7	COBL	23242
HRS EUR Dementia Wave 8	7	GATS	352954
HRS EUR Dementia Wave 8	7	SPDYE3	441272
HRS EUR Dementia Wave 8	7	PMS2P1	5379
HRS EUR Dementia Wave 8	7	PILRB	29990
HRS EUR Dementia Wave 8	7	ZCWPW1	55063
HRS EUR Dementia Wave 8	7	MEPCE	56257
HRS EUR Dementia Wave 8	7	C7orf47	221908
HRS EUR Dementia Wave 8	7	C7orf61	402573
HRS EUR Dementia Wave 8	7	TSC22D4	81628
HRS EUR Dementia Wave 8	7	C7orf51	222950
HRS EUR Dementia Wave 8	8	ARHGEF10	9639

HRS EUR Dementia Wave 8	9	C9orf135	138255
HRS EUR Dementia Wave 8	9	ZBTB34	403341
HRS EUR Dementia Wave 8	10	ZNF438	220929
HRS EUR Dementia Wave 8	11	TRPM5	29850
HRS EUR Dementia Wave 8	11	MADD	8567
HRS EUR Dementia Wave 8	11	MYBPC3	4607
HRS EUR Dementia Wave 8	11	CCDC83	220047
HRS EUR Dementia Wave 8	11	PICALM	8301
HRS EUR Dementia Wave 8	11	DDX10	1662
HRS EUR Dementia Wave 8	12	CSRP2	1466
HRS EUR Dementia Wave 8	12	WSCD2	9671
HRS EUR Dementia Wave 8	12	CMKLR1	1240
HRS EUR Dementia Wave 8	12	LOC100128554	1E+08
HRS EUR Dementia Wave 8	15	ATP8B4	79895
HRS EUR Dementia Wave 8	15	SLC27A2	11001
HRS EUR Dementia Wave 8	15	SCAPER	49855
HRS EUR Dementia Wave 8	16	CDH11	1009
HRS EUR Dementia Wave 8	16	ZCCHC14	23174
HRS EUR Dementia Wave 8	17	PRPF8	10594
HRS EUR Dementia Wave 8	17	C17orf91	84981
HRS EUR Dementia Wave 8	17	WDR81	124997
HRS EUR Dementia Wave 8	17	FLJ45079	400624
HRS EUR Dementia Wave 8	18	LOXHD1	125336
HRS EUR Dementia Wave 8	18	TNFRSF11A	8792
HRS EUR Dementia Wave 8	19	AZU1	566
HRS EUR Dementia Wave 8	19	PRTN3	5657
HRS EUR Dementia Wave 8	19	ELANE	1991
HRS EUR Dementia Wave 8	19	CFD	1675
HRS EUR Dementia Wave 8	20	REM1	28954
HRS EUR Dementia Wave 8	21	CCT8	10694
HRS EUR Dementia Wave 8	21	COL18A1	80781
HRS EUR Dementia Wave 8	21	SLC19A1	6573
HRS EUR Dementia Wave 8	22	WNT7B	7477
HRS AFR Dementia Wave 8	1	FOXD3	27022
HRS AFR Dementia Wave 8	1	S100A2	6273
HRS AFR Dementia Wave 8	1	S100A16	140576
HRS AFR Dementia Wave 8	1	S100A14	57402
HRS AFR Dementia Wave 8	2	MEIS1	4211
HRS AFR Dementia Wave 8	3	GP9	2815
HRS AFR Dementia Wave 8	3	RAB43	339122
HRS AFR Dementia Wave 8	4	SLC10A4	201780

HRS AFR Dementia Wave 8	4	ZAR1	326340
HRS AFR Dementia Wave 8	4	FRYL	285527
HRS AFR Dementia Wave 8	4	KIAA0114	57291
HRS AFR Dementia Wave 8	4	SNORA26	677810
HRS AFR Dementia Wave 8	4	STOX2	56977
HRS AFR Dementia Wave 8	5	TPPP	11076
HRS AFR Dementia Wave 8	6	NCRNA00240	1E+08
HRS AFR Dementia Wave 8	6	LOC100270746	1E+08
HRS AFR Dementia Wave 8	6	SGK1	6446
HRS AFR Dementia Wave 8	6	PDE7B	27115
HRS AFR Dementia Wave 8	7	CTTNBP2	83992
HRS AFR Dementia Wave 8	8	NIPAL2	79815
HRS AFR Dementia Wave 8	8	SQLE	6713
HRS AFR Dementia Wave 8	9	SOHLH1	402381
HRS AFR Dementia Wave 8	9	KCNT1	57582
HRS AFR Dementia Wave 8	10	WDR37	22884
HRS AFR Dementia Wave 8	10	NCRNA00200	399706
HRS AFR Dementia Wave 8	10	DRGX	644168
HRS AFR Dementia Wave 8	11	PATE3	1E+08
HRS AFR Dementia Wave 8	11	VPS26B	112936
HRS AFR Dementia Wave 8	11	THYN1	29087
HRS AFR Dementia Wave 8	11	ACAD8	27034
HRS AFR Dementia Wave 8	11	GLB1L3	112937
HRS AFR Dementia Wave 8	12	DDX11	1663
HRS AFR Dementia Wave 8	12	LALBA	3906
HRS AFR Dementia Wave 8	12	ADCY6	112
HRS AFR Dementia Wave 8	12	CACNB3	784
HRS AFR Dementia Wave 8	12	DDX23	9416
HRS AFR Dementia Wave 8	12	RND1	27289
HRS AFR Dementia Wave 8	12	CSRP2	1466
HRS AFR Dementia Wave 8	12	LOC100128554	1E+08
HRS AFR Dementia Wave 8	14	ACTN1	87
HRS AFR Dementia Wave 8	14	BEGAIN	57596
HRS AFR Dementia Wave 8	15	MEIS2	4212
HRS AFR Dementia Wave 8	17	SLC47A1	55244
HRS AFR Dementia Wave 8	17	SOCS7	30837
HRS AFR Dementia Wave 8	18	MBP	4155
HRS AFR Dementia Wave 8	19	ZNF577	84765
HRS AFR Dementia Wave 8	19	ZNF649	65251
HRS AFR Dementia Wave 8	20	DEFB124	245937
HRS AFR Dementia Wave 8	20	REM1	28954

HRS AMR Dementia Wave 8	1	YTHDF2	51441
HRS AMR Dementia Wave 8	1	HHIPL2	79802
HRS AMR Dementia Wave 8	1	TAF1A	9015
HRS AMR Dementia Wave 8	2	LASS6	253782
HRS AMR Dementia Wave 8	3	UBP1	7342
HRS AMR Dementia Wave 8	3	RAB43	339122
HRS AMR Dementia Wave 8	5	FAM134B	54463
HRS AMR Dementia Wave 8	7	SUN1	23353
HRS AMR Dementia Wave 8	7	MCM7	4176
HRS AMR Dementia Wave 8	7	AP4M1	9179
HRS AMR Dementia Wave 8	7	TAF6	6878
HRS AMR Dementia Wave 8	7	CNPY4	245812
HRS AMR Dementia Wave 8	7	MBLAC1	255374
HRS AMR Dementia Wave 8	7	C7orf59	389541
HRS AMR Dementia Wave 8	7	C7orf43	55262
HRS AMR Dementia Wave 8	7	GAL3ST4	79690
HRS AMR Dementia Wave 8	7	GPC2	221914
HRS AMR Dementia Wave 8	7	STAG3	10734
HRS AMR Dementia Wave 8	7	SLC12A9	56996
HRS AMR Dementia Wave 8	7	TRIP6	7205
HRS AMR Dementia Wave 8	7	NCRNA00244	64433
HRS AMR Dementia Wave 8	9	DNAI1	27019
HRS AMR Dementia Wave 8	9	DBC1	1620
HRS AMR Dementia Wave 8	10	C10orf122	387718
HRS AMR Dementia Wave 8	11	PDE3B	5140
HRS AMR Dementia Wave 8	11	CYP2R1	120227
HRS AMR Dementia Wave 8	11	SLC22A12	116085
HRS AMR Dementia Wave 8	11	DDX10	1662
HRS AMR Dementia Wave 8	11	FAM55B	120406
HRS AMR Dementia Wave 8	12	PLCZ1	89869
HRS AMR Dementia Wave 8	12	LALBA	3906
HRS AMR Dementia Wave 8	12	ANKRD52	283373
HRS AMR Dementia Wave 8	12	COQ10A	93058
HRS AMR Dementia Wave 8	12	CS	1431
HRS AMR Dementia Wave 8	12	WSCD2	9671
HRS AMR Dementia Wave 8	12	ARPC3	10094
HRS AMR Dementia Wave 8	12	GPN3	51184
HRS AMR Dementia Wave 8	12	C12orf24	29902
HRS AMR Dementia Wave 8	12	VPS29	51699
HRS AMR Dementia Wave 8	12	RAD9B	144715
HRS AMR Dementia Wave 8	12	PPTC7	160760

HRS AMR Dementia Wave 8	12	ANKLE2	23141
HRS AMR Dementia Wave 8	12	GOLGA3	2802
HRS AMR Dementia Wave 8	13	NUPL1	9818
HRS AMR Dementia Wave 8	16	NTAN1	123803
HRS AMR Dementia Wave 8	16	RRN3	54700
HRS AMR Dementia Wave 8	16	FBXO31	79791
HRS AMR Dementia Wave 8	17	SMG6	23293
HRS AMR Dementia Wave 8	17	SRR	63826
HRS AMR Dementia Wave 8	19	CIRBP	1153
HRS AMR Dementia Wave 8	19	C19orf24	55009
HRS AMR Dementia Wave 8	19	EFNA2	1943
HRS AMR Dementia Wave 8	19	MUM1	84939
HRS AMR Dementia Wave 8	20	BCL2L1	598
HRS ALL Dementia Wave 9	1	PEX14	5195
HRS ALL Dementia Wave 9	1	ZSCAN20	7579
HRS ALL Dementia Wave 9	1	FOXD3	27022
HRS ALL Dementia Wave 9	1	ALG6	29929
HRS ALL Dementia Wave 9	1	C1orf112	55732
HRS ALL Dementia Wave 9	1	HHIPL2	79802
HRS ALL Dementia Wave 9	2	NDUFA10	4705
HRS ALL Dementia Wave 9	4	SLC7A11	23657
HRS ALL Dementia Wave 9	5	PCDHB1	29930
HRS ALL Dementia Wave 9	7	FSCN1	6624
HRS ALL Dementia Wave 9	7	RNF216	54476
HRS ALL Dementia Wave 9	10	WDR37	22884
HRS ALL Dementia Wave 9	10	NCRNA00200	399706
HRS ALL Dementia Wave 9	10	EIF5AL1	143244
HRS ALL Dementia Wave 9	10	SFTPA2	729238
HRS ALL Dementia Wave 9	10	MAT1A	4143
HRS ALL Dementia Wave 9	10	FAM178A	55719
HRS ALL Dementia Wave 9	11	PATE4	399968
HRS ALL Dementia Wave 9	11	CDON	50937
HRS ALL Dementia Wave 9	12	HMGA2	8091
HRS ALL Dementia Wave 9	12	ZFC3H1	196441
HRS ALL Dementia Wave 9	12	THAP2	83591
HRS ALL Dementia Wave 9	12	TMEM19	55266
HRS ALL Dementia Wave 9	12	LOC100128554	1E+08
HRS ALL Dementia Wave 9	13	PHF2P1	266695
HRS ALL Dementia Wave 9	13	SLITRK1	114798
HRS ALL Dementia Wave 9	15	NRG4	145957
HRS ALL Dementia Wave 9	15	FAM174B	400451

HRS ALL Dementia Wave 9	16	LOC652276	652276
HRS ALL Dementia Wave 9	16	FLJ42627	645644
HRS ALL Dementia Wave 9	16	COX4NB	10328
HRS ALL Dementia Wave 9	17	ALOX15	246
HRS ALL Dementia Wave 9	18	RAB27B	5874
HRS ALL Dementia Wave 9	19	PODNL1	79883
HRS ALL Dementia Wave 9	19	DCAF15	90379
HRS ALL Dementia Wave 9	19	RFX1	5989
HRS ALL Dementia Wave 9	19	LILRB4	11006
HRS ALL Dementia Wave 9	19	LILRP2	79166
HRS EUR Dementia Wave 9	1	PEX14	5195
HRS EUR Dementia Wave 9	1	YTHDF2	51441
HRS EUR Dementia Wave 9	1	ZSCAN20	7579
HRS EUR Dementia Wave 9	1	HHIPL2	79802
HRS EUR Dementia Wave 9	2	DPY30	84661
HRS EUR Dementia Wave 9	2	ATOH8	84913
HRS EUR Dementia Wave 9	2	TANK	10010
HRS EUR Dementia Wave 9	3	CCDC54	84692
HRS EUR Dementia Wave 9	5	ZCCHC10	54819
HRS EUR Dementia Wave 9	5	PCDHB1	29930
HRS EUR Dementia Wave 9	5	GABRG2	2566
HRS EUR Dementia Wave 9	6	EXOC2	55770
HRS EUR Dementia Wave 9	7	FSCN1	6624
HRS EUR Dementia Wave 9	7	RNF216	54476
HRS EUR Dementia Wave 9	7	PMS2P11	441263
HRS EUR Dementia Wave 9	9	C9orf135	138255
HRS EUR Dementia Wave 9	10	EIF5AL1	143244
HRS EUR Dementia Wave 9	10	SFTPA2	729238
HRS EUR Dementia Wave 9	10	MAT1A	4143
HRS EUR Dementia Wave 9	11	BDNF-AS1	497258
HRS EUR Dementia Wave 9	11	WNT11	7481
HRS EUR Dementia Wave 9	11	CCDC83	220047
HRS EUR Dementia Wave 9	12	TMEM19	55266
HRS EUR Dementia Wave 9	12	PTPRQ	374462
HRS EUR Dementia Wave 9	15	SNURF	8926
HRS EUR Dementia Wave 9	15	NRG4	145957
HRS EUR Dementia Wave 9	16	HAGH	3029
HRS EUR Dementia Wave 9	16	FAHD1	81889
HRS EUR Dementia Wave 9	16	C16orf73	254528
HRS EUR Dementia Wave 9	16	PDXDC1	23042
HRS EUR Dementia Wave 9	16	NTAN1	123803

HRS EUR Dementia Wave 9	16	COX4NB	10328
HRS EUR Dementia Wave 9	16	COX4I1	1327
HRS EUR Dementia Wave 9	20	CDH26	60437
HRS EUR Dementia Wave 9	22	RTN4R	65078
HRS EUR Dementia Wave 9	22	WNT7B	7477
HRS AFR Dementia Wave 9	2	NDUFS1	4719
HRS AFR Dementia Wave 9	2	EEF1B2	1933
HRS AFR Dementia Wave 9	2	SNORD51	26798
HRS AFR Dementia Wave 9	2	SNORA41	619569
HRS AFR Dementia Wave 9	2	GPR1	2825
HRS AFR Dementia Wave 9	4	SCFD2	152579
HRS AFR Dementia Wave 9	4	SPATA5	166378
HRS AFR Dementia Wave 9	6	NCRNA00240	1E+08
HRS AFR Dementia Wave 9	6	LOC100270746	1E+08
HRS AFR Dementia Wave 9	6	ZBTB2	57621
HRS AFR Dementia Wave 9	7	GET4	51608
HRS AFR Dementia Wave 9	7	ADAP1	11033
HRS AFR Dementia Wave 9	8	LPL	4023
HRS AFR Dementia Wave 9	8	PIWIL2	55124
HRS AFR Dementia Wave 9	8	PEBP4	157310
HRS AFR Dementia Wave 9	9	SOHLH1	402381
HRS AFR Dementia Wave 9	9	KCNT1	57582
HRS AFR Dementia Wave 9	10	WDR37	22884
HRS AFR Dementia Wave 9	10	NCRNA00200	399706
HRS AFR Dementia Wave 9	11	ANO1	55107
HRS AFR Dementia Wave 9	11	ACRV1	56
HRS AFR Dementia Wave 9	11	PATE1	160065
HRS AFR Dementia Wave 9	11	PATE2	399967
HRS AFR Dementia Wave 9	11	PATE3	1E+08
HRS AFR Dementia Wave 9	11	PATE4	399968
HRS AFR Dementia Wave 9	11	HYLS1	219844
HRS AFR Dementia Wave 9	11	PUS3	83480
HRS AFR Dementia Wave 9	11	DDX25	29118
HRS AFR Dementia Wave 9	11	CDON	50937
HRS AFR Dementia Wave 9	12	LALBA	3906
HRS AFR Dementia Wave 9	12	MFSD5	84975
HRS AFR Dementia Wave 9	12	ESPL1	9700
HRS AFR Dementia Wave 9	12	PFDN5	5204
HRS AFR Dementia Wave 9	12	C12orf10	60314
HRS AFR Dementia Wave 9	12	AAAS	8086
HRS AFR Dementia Wave 9	12	SP7	121340

HRS AFR Dementia Wave 9	12	ZFC3H1	196441
HRS AFR Dementia Wave 9	12	THAP2	83591
HRS AFR Dementia Wave 9	12	TMEM19	55266
HRS AFR Dementia Wave 9	12	CMKLR1	1240
HRS AFR Dementia Wave 9	12	ANKLE2	23141
HRS AFR Dementia Wave 9	12	GOLGA3	2802
HRS AFR Dementia Wave 9	14	COX16	51241
HRS AFR Dementia Wave 9	14	SYNJ2BP	55333
HRS AFR Dementia Wave 9	14	ADAM21	8747
HRS AFR Dementia Wave 9	14	ADAM20	8748
HRS AFR Dementia Wave 9	14	NCRNA00221	1E+08
HRS AFR Dementia Wave 9	17	PRPF8	10594
HRS AFR Dementia Wave 9	17	SLC47A1	55244
HRS AFR Dementia Wave 9	18	RAB27B	5874
HRS AFR Dementia Wave 9	19	ATG4D	84971
HRS AFR Dementia Wave 9	19	KRI1	65095
HRS AFR Dementia Wave 9	19	CDKN2D	1032
HRS AFR Dementia Wave 9	19	PODNL1	79883
HRS AFR Dementia Wave 9	19	DCAF15	90379
HRS AFR Dementia Wave 9	19	RFX1	5989
HRS AFR Dementia Wave 9	19	ZNF577	84765
HRS AFR Dementia Wave 9	19	ZNF649	65251
HRS AFR Dementia Wave 9	20	RNF24	11237
HRS AFR Dementia Wave 9	21	PTTG1IP	754
HRS AFR Dementia Wave 9	21	ITGB2	3689
HRS AFR Dementia Wave 9	21	C21orf67	84536
HRS AFR Dementia Wave 9	21	C21orf70	85395
HRS AFR Dementia Wave 9	21	COL18A1	80781
HRS AFR Dementia Wave 9	21	SLC19A1	6573
HRS AFR Dementia Wave 9	22	FLJ41941	1E+08
HRS AMR Dementia Wave 9	1	MOSC2	54996
HRS AMR Dementia Wave 9	1	MOSC1	64757
HRS AMR Dementia Wave 9	1	HHIPL2	79802
HRS AMR Dementia Wave 9	2	DPY30	84661
HRS AMR Dementia Wave 9	2	EPAS1	2034
HRS AMR Dementia Wave 9	2	GPR1	2825
HRS AMR Dementia Wave 9	2	ZDBF2	57683
HRS AMR Dementia Wave 9	3	KCNH8	131096
HRS AMR Dementia Wave 9	3	PDIA5	10954
HRS AMR Dementia Wave 9	4	SLC26A1	10861
HRS AMR Dementia Wave 9	4	IDUA	3425

HRS AMR Dementia Wave 9	4	FGFRL1	53834
HRS AMR Dementia Wave 9	4	STOX2	56977
HRS AMR Dementia Wave 9	5	NEURL1B	54492
HRS AMR Dementia Wave 9	6	SLC44A4	80736
HRS AMR Dementia Wave 9	6	EHMT2	10919
HRS AMR Dementia Wave 9	6	ZBTB12	221527
HRS AMR Dementia Wave 9	6	C2	717
HRS AMR Dementia Wave 9	6	CFB	629
HRS AMR Dementia Wave 9	6	ZNF292	23036
HRS AMR Dementia Wave 9	6	GJB7	375519
HRS AMR Dementia Wave 9	7	FSCN1	6624
HRS AMR Dementia Wave 9	7	MUC17	140453
HRS AMR Dementia Wave 9	7	IMPDH1	3614
HRS AMR Dementia Wave 9	7	C7orf68	29923
HRS AMR Dementia Wave 9	9	KIAA1161	57462
HRS AMR Dementia Wave 9	9	C9orf24	84688
HRS AMR Dementia Wave 9	9	C9orf25	203259
HRS AMR Dementia Wave 9	9	DNAI1	27019
HRS AMR Dementia Wave 9	10	GAD2	2572
HRS AMR Dementia Wave 9	11	CCDC83	220047
HRS AMR Dementia Wave 9	12	DBX2	440097
HRS AMR Dementia Wave 9	12	RACGAP1P	83956
HRS AMR Dementia Wave 9	12	NR1H4	9971
HRS AMR Dementia Wave 9	12	GAS2L3	283431
HRS AMR Dementia Wave 9	12	WSCD2	9671
HRS AMR Dementia Wave 9	16	CLCN7	1186
HRS AMR Dementia Wave 9	16	PTX4	390667
HRS AMR Dementia Wave 9	16	TELO2	9894
HRS AMR Dementia Wave 9	16	IFT140	9742
HRS AMR Dementia Wave 9	16	CACNG3	10368
HRS AMR Dementia Wave 9	17	NCRNA00188	125144
HRS AMR Dementia Wave 9	17	C17orf76	388341
HRS AMR Dementia Wave 9	17	SOCS7	30837
HRS AMR Dementia Wave 9	17	INTS2	57508
HRS AMR Dementia Wave 9	17	MED13	9969
HRS AMR Dementia Wave 9	17	ABCA8	10351
HRS AMR Dementia Wave 9	22	WNT7B	7477
Longevity > 90	1	FCRL4	83417
Longevity > 90	2	DNMT3A	1788
Longevity > 90	4	DHX15	1665
Longevity > 90	5	HOMER1	9456

Longevity > 90	5	RPL26L1	51121
Longevity > 90	7	CALN1	83698
Longevity > 90	10	DCLRE1C	64421
Longevity > 90	10	MEIG1	644890
Longevity > 90	10	C10orf122	387718
Longevity > 90	10	FANK1	92565
Longevity > 90	11	AMICA1	120425
Longevity > 90	11	HYLS1	219844
Longevity > 90	11	PUS3	83480
Longevity > 90	11	DDX25	29118
Longevity > 90	12	RASSF8	11228
Longevity > 90	12	GOLGA3	2802
Longevity > 90	14	ACTN1	87
Longevity > 90	14	SYNJ2BP	55333
Longevity > 90	14	ADAM21	8747
Longevity > 90	14	ADAM20	8748
Longevity > 90	15	TYRO3	7301
Longevity > 90	15	ATP8B4	79895
Longevity > 90	19	KIR3DL1	3811
Longevity > 90	20	CDH26	60437
Longevity > 90	20	C20orf197	284756
Parkinsons	1	FAM41C	284593
Parkinsons	1	ZSCAN20	7579
Parkinsons	1	PPIE	10450
Parkinsons	1	BMP8B	656
Parkinsons	1	TRIT1	54802
Parkinsons	1	MOSC2	54996
Parkinsons	1	MOSC1	64757
Parkinsons	2	TANK	10010
Parkinsons	3	KCTD6	200845
Parkinsons	3	ACOX2	8309
Parkinsons	3	XRN1	54464
Parkinsons	4	TMEM175	84286
Parkinsons	4	DGKQ	1609
Parkinsons	4	SLC26A1	10861
Parkinsons	4	IDUA	3425
Parkinsons	4	FGFRL1	53834
Parkinsons	5	BTF3	689
Parkinsons	5	ANKRA2	57763
Parkinsons	5	UTP15	84135
Parkinsons	5	KIF3A	11127

Parkinsons	5	CCNI2		645121
Parkinsons	5		8-Sep	23176
Parkinsons	6	HSP90AB1		3326
Parkinsons	7	NXPH1		30010
Parkinsons	7	ZCWPW1		55063
Parkinsons	7	MEPCE		56257
Parkinsons	7	C7orf47		221908
Parkinsons	7	C7orf61		402573
Parkinsons	7	TSC22D4		81628
Parkinsons	7	C7orf51		222950
Parkinsons	9	PRUNE2		158471
Parkinsons	10	CUL2		8453
Parkinsons	10	CREM		1390
Parkinsons	10	CCNY		219771
Parkinsons	10	FAM178A		55719
Parkinsons	11	CASP1		834
Parkinsons	11	CARD16		114769
Parkinsons	12	HIST4H4		121504
Parkinsons	12	H2AFJ		55766
Parkinsons	12	WBP11		51729
Parkinsons	12	C12orf60		144608
Parkinsons	12	C12orf69		440087
Parkinsons	12	ARID2		196528
Parkinsons	13	RCBTB2		1102
Parkinsons	13	UCHL3		7347
Parkinsons	14	GPR137C		283554
Parkinsons	14	ERO1L		30001
Parkinsons	14	ACTN1		87
Parkinsons	14	SYNJ2BP		55333
Parkinsons	14	ADAM21		8747
Parkinsons	14	ADAM20		8748
Parkinsons	14	MED6		10001
Parkinsons	14	TTC9		23508
Parkinsons	14	RBM25		58517
Parkinsons	15	C15orf60		283677
Parkinsons	16	MVP		9961
Parkinsons	16	NDRG4		65009
Parkinsons	16	SETD6		79918
Parkinsons	16	CNOT1		23019
Parkinsons	16	CDH8		1006
Parkinsons	17	RPH3AL		9501

Parkinsons	19	ATG4D	84971
Parkinsons	19	KRI1	65095
Parkinsons	19	CDKN2D	1032
Parkinsons	19	ZNF577	84765
Parkinsons	19	ZNF350	59348
Parkinsons	19	ZNF615	284370
Parkinsons	19	ZNF614	80110
Parkinsons	19	ZNF432	9668
Parkinsons	21	RRP1B	23076
Parkinsons	21	PDXK	8566
Parkinsons	21	CSTB	1476

Supplementary Table 7: Characteristics of the HRS individuals

We retrieved the HRS longitudinal data during 1996 to 2010 (Waves 3 to 10). The characters of study individuals are listed below including the distributions of three cognitive functioning measurements used for GWAS analysis. In particular, the cognitive slope is a measure that assesses the change in cognitive age given the change in chronological age over the fourteen years (1996-2010).

Genetic Ancestry	N	Male	Age at Wave 3	Age at Wave 9	College[a]	Cognitive slope	Dementia Wave 8 (case/control)	Dementia Wave 9 (case/control)
ALL	12452	41%	62 (7.4) [50,90]	70 (9.5) [50,102]	22%	1.04 (0.68) [-7.13, 14]	401 (4.6%)/8396 (95.4%)	490 (5.8%)/7922 (94.2%)
EUR	9564	42%	62 (7.5) [50,90]	71 (9.5) [50,100]	25%	1.05 (0.64) [-6.33, 12.2]	177 (2.5%)/6982 (97.5%)	254 (3.7%)/6580 (96.3%)
AFR	1553	36%	62 (7.1) [50,89]	69 (9.2) [50,102]	12%	1.04 (0.82) [-7.13, 14]	143 (16%)/750 (84%)	149 (18%)/702 (82%)
AMR	1035	40%	61 (6.7) [50,84]	67 (9.2) [52,96]	7%	0.96 (0.73) [-2.67, 5.1]	66 (11%)/518 (89%)	70 (13%)/488 (87%)
ASN	91	32%	61 (6.3) [50,80]	69 (9.6) [54,92]	47%	1.09 (0.92) [-0.65, 6.38]	1 (2%)/48 (98%)	1 (1.9%)/52 (98.1%)
Others	209	32%	61 (7.2) [50,79]	70 (9.2) [54,92]	14%	0.99 (0.81) [-4.98, 3.47]	14 (12%)/98 (88%)	16 (14%)/100 (86%)

Quantitative variables are presented in the format of mean(sd) [range].
Abbreviations: ALL=all genetic ancestry; EUR=Europeans; AFR=Africans; AMR=Americas; ANS=Asians.
[a]College and above.

Supplementary Table 8: Genomic inflation estimates for cognitive traits in the HRS

We report genomic inflation estimates λ_{GC} for cognitive functioning trait in different ethnic strata of the Health and Retirement Study. Column 2 reports the group of subjects with "ALL" denoting all samples combined irrespective of race/ethnicity. The GWAS was conducted using the number of SNPs (column "No. SNPs" with common variants (MAF ≥ 5%)). To protect against spurious association results, the analysis was adjusted for principal components (PCs), gender, and education (college or above).

Trait	Genetic ancestry	λ_{GC}	No. SNPs	No. PCs	Age	Gender	College[a]
**Cognitive slope**	ALL	0.99	4754646	4	N	Y	Y
	EUR	1.00	5798029	2	N	Y	Y
	AFR	1.00	7982837	2	N	Y	Y
	AMR	0.99	5888282	2	N	Y	Y
**Dementia (Wave 8)**	ALL	0.99	5330729	4	Y	Y	Y
	EUR	1.00	5797728	2	Y	Y	Y
	AFR	1.02	7598575	2	Y	Y	Y
	AMR	1.00	5811141	2	Y	Y	Y
**Dementia (Wave 9)**	ALL	1.01	5356866	4	Y	Y	Y
	EUR	1.00	5798029	2	Y	Y	Y
	AFR	1.00	7598997	2	Y	Y	Y
	AMR	0.97	5806311	2	Y	Y	Y

[a]Education for college and above.

Supplementary Notes

Supplementary Note 1: Description of datasets

Individuals of European ancestry from each study were included in our GWA meta-analysis. Genetic ancestry was identified in PLINK or EIGENSTRAT[1] for each study, respectively. Here we describe resources of each study and disease status of study individuals.

Study 1: These brain tissues are part of the study samples used in a study for Alzheimer's disease[2], archived in the MRC London Brain bank for Neurodegenerative Disease. We obtained genotyping and DNA methylation data for 63 individuals, including 38 diagnosed with Alzheimer's disease. DNA methylation data are available for free public download (Supplementary Table 1). Individuals with missing age acceleration estimates were removed from the GWAS, yielding 59 remaining subjects. **Study 2**: All of the 148 individuals were neurologically normal[3]. The SNP data was archived in dbGAP, http://www.ncbi.nlm.nih.gov/gap, with accession: phs000249.v1.p1. The GWAS was conducted in 112 individuals with age acceleration estimates for CRBLM tissues. Gene expression and DNA methylation data are available for free public download (Supplementary Table 1).

Study 3: The 153 individuals were used for a case control (121 cases/32 controls) study for psychiatric disorders[4]. The SNP data was downloaded from Stanley Medical Research Institute https://www.stanleygenomics.org/stanley/standard/studyDetail.jsp?study_id=20. Assays of genotyping and DNA methylation profiled in 147 individuals were used in our study. Gene expression and DNA methylation data are available for free public download (Supplementary Table 1).**Study 4**: We obtained genotyping and DNA methylation data for 44 individuals from a

case control study for Schizophrenia in [5]. Of those, 36 individuals (19 controls/ 17 cases) verified of European ancestry were used in our study. DNA methylation data are available for free public download (Supplementary Table 1).

Study 5: All subjects (n=232) were neurologically normal[6]. The SNP data was archived in dbGAP, http://www.ncbi.nlm.nih.gov/gap, with accession: phs000249.v2.p1. We excluded 3 individuals genotyped in a different platform, 6 identified as genetic outliers, 13 who failed the QC in DNA methylation arrays in terms of ambiguous gender, DNAmAge estimates outliers (deviating from chronological age greater than 40 years). In addition, ambiguous gender was indicated by the predicted gender via the algorithm of DNAm age calculation (see Methods). This yields a total of 201 individuals, with both genotyping and DNA methylation data available, remaining in the GWAS. Gene expression and DNA methylation data are available for free public download (Supplementary Table 1).

Study 6: A total of 88 neuron tissues were collected, with six removed due to lack of mRNA data, and one due to ambiguous gender indicated by the predicted gender via the algorithm of DNAm age calculation (see Methods). Thus, 81 samples (consisting of 26 neurologically normal and 55 subjects diagnosed with psychiatric traits) remained in cis-eQTL analysis. All brain tissues were dissected from the orbital frontal cortex less than 24 hours post-mortem. Although very precise anatomically, the dissected specimens still contained slightly different ratios between grey (mostly neurons) and white (mostly glial cells) matter. However, when we sorted neuronal from glia nuclei by fluorescent-activated cell sorting (FACS), we recorded the numbers of neuronal and glial nuclei in each sample. This measure turned out to be very reproducible. Transcriptional profiling was performed using mRNA-seq. Libraries were prepared from 1 ug of total RNA using Illumina's TruSeq RNA library preparation kit V2, and sequencing was

performed using an Illumina HiSeq2000. After de-multiplexing of each library pool according to its barcode sequence, from 8 to 20 million (M) (mean 15.1 M), reads per sample were available for analysis. Alignment, assembly and quantification of the data were performed using the TopHat 2.0.8b and Cufflinks 2.1.1.

Supplementary Note 2: Age-related studies used in our overlap analysis (hypergeometric test)

Below, we briefly describe a total of 5 large scale GWAS studies that were cross referenced to our GWAS study of epigenetic age acceleration. The GWAS results are corresponding to previously published articles, except those for cognitive functioning traits using the Health and Retirement Study (HRS) data. We performed GWAS for three cognitive functioning traits as described in more details below with the other 4 studies.

Age-related macular degeneration (AMD)

A large-scale GWAS meta-analysis was performed in the study including >17,100 advanced AMD cases and >60,000 controls of European and Asian ancestry in the analysis[7], conducted by AMD Gene Consortium Study of Age Related Macular Degeneration. We downloaded the summary results of ~ 2.4 million markers for studying advanced AMD versus control subjects, from http://www.sph.umich.edu/csg/abecasis/public/amdgene2012/.

Alzheimer's disease

The IGAP consortium performed a GWAS meta-analysis on 74,046 individuals of European ancestry[8]. We downloaded the summary results of GWAS from http://www.pasteur-lille.fr/en/recherche/u744/igap/igap_download.php. Two sets of association results are available.

The first set includes the GWAS results of meta-analysis based upon 17,008 Alzheimer's disease cases and 37,154 controls at stage 1 analysis. A total of 11,632 SNPs exhibited moderate evidence of association ($P < 1.0 \times 10^{-3}$) at stage 1. The second set includes the P values of the 11,632 SNPs from the final meta-analysis that combined stages 1 &2 results. We used the association results that combined two stages analyses results for our overlap analysis.

The Health and Retirement Study (HRS)

The Health and Retirement Study (1992-2012) is a longitudinal panel study of a representative sample of Americans over age 50 (and their spouses), collected every two years (**Supplementary Table 6**). We downloaded study materials, genotype (including imputed markers) and phenotype data of ~ 12,500 individuals from dbGAP with access: phs000428.v1.p1. To evaluate the genetic variants overlapping between epigenetic age acceleration and cognitive functioning, GWAS was performed on genotyped and imputed markers for three relative traits: (1) cognitive aging slope, (2) binary dementia status at wave 8 (diagnosed in year 2006), and (3) binary dementia status at wave 9 (diagnosed in year 2008), respectively. The procedures of GWAS and the definitions of the traits are descried below.

GWAS in the HRS

We performed association analysis on genotyped and imputed SNPs with common variants. Genotyping data were performed on Illumina's Human Omni2.5-Quad (Omni2.5) platform and imputed data were computed with IMPUTE2. Quality control of SNPs was guided by HWE $P > 1.0 \times 10^{-6}$ along with info measure > 0.4 for imputed markers and thresholded genotypes set at 0.9 for imputed genotypes. In addition, we required minimum number of samples at 200 per marker. Since the HRS study is comprised of different racial/ethnic groups, we either restricted the GWAS analysis to a given ethnic group or used principal components (from an identity by state analysis) in multivariate regression models. Post association analysis, we pruned out the SNPs associated with large effect sizes guided by odds ratios (> 3 or < 1/3). More details for model framework and assessments for GWAS results can be found in **Supplementary Table 6**.

Cognitive slope for measuring cognitive decline in the HRS

The cognitive slope defines the change in cognitive age given the change in chronological age over fourteen years (1996-2010). To calculate the slope, we determined cognitive ages for each participant at each wave, based on data from wave three (1996) through wave ten (2010) for four measures of cognitive functioning—delayed recall, immediate recall, serial 7s, and backwards counting. For all waves, the score for immediate and delayed recall were both based on the number of words correctly recalled out of ten possible. The delayed recall was assessed approximately five minutes after the word list was read, between which other tasks were performed. The scores for serial 7s ranged from 0-5. Respondents were asked to continuously subtract by 7, starting from 100. Points were awarded for correct responses for a total of five subtractions. For backwards counting scores, respondents were asked to count backwards, 10 consecutive times, beginning with the number 20. A total of two points were allotted for two attempts at backwards counting—participants received a zero if they were incorrect on both tries; a 1 if they were incorrect on the first try, but correct on the second; and a 2 if they correctly completed the task on the first try.

A cognitive age was calculated for each participant at each wave. The method for calculating cognitive age was based on an algorithm proposed by Klemera & Doubal[9] that is typically used for calculating biological ages from biomarker data[10]. This method has been validated using both real and simulated data. Cognitive age estimates combine information from equations of chronological age regressed on each of the cognitive functioning markers. The equation for calculating cognitive age is:

$$\text{Cognitive age} = \frac{\sum_{j=1}^{m}(x_{ji}-q_j)\frac{k_j}{s_j^2}+\frac{CA_i}{s_{BA}^2}}{\sum_{j=1}^{m}\left(\frac{k_j}{s_j}\right)^2+\frac{1}{s_{BA}^2}}$$

Where, k_j and q_j are the slope and intercept, respectively, for the regression of chronological age and each cognitive measure, x_{ji} is the value of cognitive measure j for participant i, s_j is the root mean squared error of chronological age regressed on the jth cognitive measure, and CA_i is chronological age for participant i. Additionally s_{BA}^2, the variance of the random variable, takes into account the variability in the first half of the equation, the mean variance of the cognitive measures that is explained by chronological age, and the range of chronological age. Overall, the mean cognitive age of a population should equal the mean chronological age of the population[9]. Once cognitive ages were determined for each participant at each wave we calculated the slope for the change in cognitive age given the change in chronological age over eight waves (1996-2010).

Dementia status in the HRS

The HRS participants with ages 70 and over (n=6,412) were classified into normal, mild cognitive impairment or cognitive impairment not demented (CIND), and dementia groups. Only normal and demented participants were used here. Predicted dementia status also relied on the four cognitive functioning variables that were used to estimate cognitive aging—delayed recall, immediate recall, serial 7s, and backwards counting. However, dementia status also took into account proxy responses for participants who were unable to complete the cognitive battery. For those who were able to respond, scores across the four variables were summed. Participants with total score ranging between 12 and 27 were categorized as having normal cognitive functioning;

those with scores between 7 and 11 were categorized as having CIND, and those with scores of six or less were categorized as having dementia.

Participants whose status relied on proxy respondents were categorized in accordance with the method proposed by Langa et al.[11]. For these participants, cognitive status was based on the sum of scores from three measures taken from memory assessments by a proxy (0=excellent, 1=very good, 2=good, 3=fair, 4=poor), the participant's total number of IADL limitations, and the interviewer's assessment of whether the participant had difficulty completing the cognitive battery due to cognitive limitations (0-2 indicating, no limitation, some limitation, and limitation prevents completion, respectively). After these three measures were summed, participants with total scores between 0 and 2 were categorized as having normal cognitive functioning, those with scores between 3 and 5 were categorized as having CIND, and those with scores of 6 or more were categorized as demented.

Longevity study

The GWAS meta-analyses study was performed in 98,066 individuals of European ancestry[12], including discovery, replication and joint analyses. The summary results at discovery phase analysis can be downloaded from http://hmg.oxfordjournals.org/content/23/16/4420/suppl/DC1. The results include two types of association P values with respect to (1) individuals with longevity > 85 versus < 65 and (2) individuals with longevity > 90 versus < 65. We only report the results for comparison (2), i.e. longevity >90 versus <65. The results for the first comparison were similar.

Parkinson's disease study

A two stage genome wide association study for Parkinson's disease was conducted in 13,625 individuals of European ancestry[13]. For our analysis, we utilized the results of 463187 SNPs at stage 1 phase released in dbGAP with accession: pha002868.1 downloaded from, http://www.ncbi.nlm.nih.gov/projects/gap/cgibin/analysis.cgi?study_id=phs000501.v1.p1&pha=2868. The positions of SNPs are aligned with hg19 assembly in all datasets except the longevity study, which used hg18 assembly.

Supplementary Note 3: Decorrelation analysis used in the meta-analysis of transcriptional data

Below, we describe fixed-effects model used for meta-analysis in R *metafor* package and follow most of the notations used in [14]. The model is given by

$$y_i = \mu\,(\theta) + e_i$$

, where y_i is the observed effect size for the *i*-th ($i = 1, \dots, k$) study and e_i is normally distributed with 0 mean and (known) variance v_i, denoted by $N(0, v_i)$. Therefore, the vector $Y = (y_{1,\dots,}y_i, .., y_k)$ is distributed as $N(\mu(\theta), diagS)$ where $\mu(\theta)$ is a k by 1 vector for the true effect size and $diagS$ is a diagonal covariance matrix with entries v_i for k independent studies. In the inverse variance weighting fixed-effect model, θ is estimated via the weighted Gauss-Newton algorithm[15] with scale parameter fixed at one, iteratively reweighted by the inverse of $diagS$.

Transformation of Y to Y*

Here, we assume that there exits correlation among the k studies such that Y~ $N(0, \Sigma)$ and the singular vector decomposition of$\Sigma = \Gamma\,\Lambda\,\Gamma'$. The decorrelation transformation is used to multiply Y by $\Sigma^{-1/2}$ such that

$$Y^{*} = \Sigma^{-1/2}Y$$

$$= \Gamma\Lambda^{-1/2}\Gamma'Y \sim N(0, I)$$

,where I is a k by k identity matrix. Therefore, in performing meta-analysis with the inverse variance weighting, we use Y^{*} to assess the P value that strictly accounts for the intra subject correlation. We applied this transformation to (1) brain eQTL analysis and (2) the robust correlation analysis between expression levels of potential functional genes and chronological age. The covariance matrix Σ can be empirically estimated based on the covariance matrix of

gene expression levels. For illustration, we consider the correlation matrix of $Y = (y_{ijk})$ for gene expression, where i is the index for study 2, 3 5, and 6, j =1, 2,…,5, is the index for brain regions CRBLM, frontal cortex, pons, temporal cortex, and neurons, respectively and k is the index for gene. Without loss of generality, we drop the gene index k and express the covariance matrix Σ of the vector $Y = (y_{21}, y_{22}, y_{23}, y_{24} y_{31}, y_{51}, y_{51}, y_{65})$ as

$$\Sigma = \begin{pmatrix} \Sigma_{22} & 0 & 0 & 0 \\ 0 & \sigma_3^2 & 0 & 0 \\ 0 & 0 & \Sigma_{55} & 0 \\ 0 & 0 & 0 & \sigma_6^2 \end{pmatrix}$$

, where Σ_{22} corresponds to the 4 by 4 covariance matrix of the study 2 samples across the 4 brain regions and Σ_{55} corresponds to the 2 by 2 covariance matrix of study 5 samples across the 2 brain regions estimated via the empirical variance and correlation matrix of $(y_{21}, y_{22}, y_{23}, y_{24})$ and (y_{51}, y_{52}), respectively; σ_3^2 and σ_6^2 are the empirical variance estimates of gene expression in CRBLM for study 3 and in neurons for study 6, respectively. We view the decorrelation analysis as a stringent correction as it entirely removes the dependence of gene expression levels across brain regions.

Supplementary References

1. Price, A.L. *et al.* Principal components analysis corrects for stratification in genome-wide association studies. *Nat Genet* **38**, 904-9 (2006).
2. Lunnon, K. *et al.* Methylomic profiling implicates cortical deregulation of ANK1 in Alzheimer's disease. *Nat Neurosci* **17**, 1164-70 (2014).
3. Gibbs, J.R. *et al.* Abundant quantitative trait loci exist for DNA methylation and gene expression in human brain. *PLoS Genet* **6**, e1000952 (2010).
4. Zhang, D. *et al.* Genetic control of individual differences in gene-specific methylation in human brain. *Am J Hum Genet* **86**, 411-9 (2010).
5. Pidsley, R. *et al.* Methylomic profiling of human brain tissue supports a neurodevelopmental origin for schizophrenia. *Genome Biol* **15**, 483 (2014).
6. Hernandez, D.G. *et al.* Distinct DNA methylation changes highly correlated with chronological age in the human brain. *Hum Mol Genet* **20**, 1164-72 (2011).
7. Fritsche, L.G. *et al.* Seven new loci associated with age-related macular degeneration. *Nat Genet* **45**, 433-9, 439e1-2 (2013).
8. Lambert, J.C. *et al.* Meta-analysis of 74,046 individuals identifies 11 new susceptibility loci for Alzheimer's disease. *Nat Genet* **45**, 1452-8 (2013).
9. Klemera, P. & Doubal, S. A new approach to the concept and computation of biological age. *Mech Ageing Dev* **127**, 240-8 (2006).
10. Levine, M.E. Modeling the rate of senescence: can estimated biological age predict mortality more accurately than chronological age? *J Gerontol A Biol Sci Med Sci* **68**, 667-74 (2013).
11. Langa, K.M. *et al.* The Aging, Demographics, and Memory Study: study design and methods. *Neuroepidemiology* **25**, 181-91 (2005).
12. Deelen, J. *et al.* Genome-wide association meta-analysis of human longevity identifies a novel locus conferring survival beyond 90 years of age. *Hum Mol Genet* **23**, 4420-32 (2014).
13. Simon-Sanchez, J. *et al.* Genome-wide association study reveals genetic risk underlying Parkinson's disease. *Nat Genet* **41**, 1308-12 (2009).
14. Viechtbauer, W. Conducting Meta-Analyses in R with the metafor Package. *Journal of Statistical Software* **36**, 1-48 (2010).
15. Jennrich, R.I. *An introduction to computational statistics : regression analysis*, xvi, 364 p. (Prentice Hall, Englewood Cliffs, N.J., 1995).
16. Langfelder, P. & Horvath, S. WGCNA: an R package for weighted correlation network analysis. *BMC Bioinformatics* **9**, 559 (2008).
17. Ernst, J. & Kellis, M. Large-scale imputation of epigenomic datasets for systematic annotation of diverse human tissues. *Nat Biotechnol* **33**, 364-76 (2015).
18. Ernst, J. *et al.* Mapping and analysis of chromatin state dynamics in nine human cell types. *Nature* **473**, 43-9 (2011).
19. Langfelder, P. & Horvath, S. WGCNA: an R package for weighted correlation network analysis. *BMC Bioinformatics* **9**, 559 (2008).
20. Miller, J.A. *et al.* Strategies for aggregating gene expression data: the collapseRows R function. *BMC Bioinformatics* **12**, 322 (2011).
21. Bult, C.J. *et al.* The Mouse Genome Database (MGD): mouse biology and model systems. *Nucleic Acids Res* **36**, D724-8 (2008).
22. Lee, T.I. *et al.* Control of developmental regulators by Polycomb in human embryonic stem cells. *Cell* **125**, 301-13 (2006).
23. Hawrylycz, M.J. *et al.* An anatomically comprehensive atlas of the adult human brain transcriptome. *Nature* **489**, 391-9 (2012).
24. Cahoy, J.D. *et al.* A transcriptome database for astrocytes, neurons, and oligodendrocytes: a new resource for understanding brain development and function. *J Neurosci* **28**, 264-78 (2008).
25. Cajigas, I.J. *et al.* The local transcriptome in the synaptic neuropil revealed by deep sequencing and high-resolution imaging. *Neuron* **74**, 453-66 (2012).
